# Identification of mosquito proteins that differentially interact with alphavirus nonstructural protein 3, a determinant of vector specificity

**DOI:** 10.1371/journal.pntd.0011028

**Published:** 2023-01-25

**Authors:** Nathaniel M. Byers, Paul L. Burns, Olga Stuchlik, Matthew S. Reed, Jeremy P. Ledermann, Jan Pohl, Ann M. Powers

**Affiliations:** 1 Division of Vector-Borne Diseases, Centers for Disease Control and Prevention, Fort Collins, Colorado, United States of America; 2 Biotechnology Core Facility Branch, Centers for Disease Control and Prevention, Atlanta, Georgia, United States of America; Faculty of Science, Ain Shams University (ASU), EGYPT

## Abstract

Chikungunya virus (CHIKV) and the closely related onyong-nyong virus (ONNV) are arthritogenic arboviruses that have caused significant, often debilitating, disease in millions of people. However, despite their kinship, they are vectored by different mosquito subfamilies that diverged 180 million years ago (anopheline versus culicine subfamilies). Previous work indicated that the nonstructural protein 3 (nsP3) of these alphaviruses was partially responsible for this vector specificity. To better understand the cellular components controlling alphavirus vector specificity, a cell culture model system of the anopheline restriction of CHIKV was developed along with a protein expression strategy. Mosquito proteins that differentially interacted with CHIKV nsP3 or ONNV nsP3 were identified. Six proteins were identified that specifically bound ONNV nsP3, ten that bound CHIKV nsP3 and eight that interacted with both. In addition to identifying novel factors that may play a role in virus/vector processing, these lists included host proteins that have been previously implicated as contributing to alphavirus replication.

## Author summary

Alphaviruses such as CHIKV and ONNV, cause severely painful human illnesses and are capable of producing large outbreaks with endemic persistence. Half of the alphavirus lifecycle is in mosquitoes, making it important to understand the molecular interactions between the virus and the vector that influence the capacity of these viruses to spread. This work identifies 24 vector proteins that may be responsible for restricting CHIKV from infecting the subfamily of mosquitoes that ONNV, CHIKV’s closest relative, uses for transmission. This is the first study to identify *Anopheles* host proteins that interact with alphavirus nonstructural protein 3. Understanding these virus/vector interactions is important for identifying risk factors for viral transmission and potential mechanisms for controlling vector infections by human pathogens.

## Introduction

Chikungunya virus (CHIKV) is most closely related to onyong-nyong virus (ONNV). Both alphaviruses cause an acute febrile illness with associated debilitating arthralgia that can persist for months or years. ONNV, which is endemic in sub-Saharan Africa, caused one of the largest arbovirus outbreaks known in 1959 to 1962, which affected over two million people [1–3]. CHIKV has also infected millions of people during reemergence events which led to the virus spreading globally during the past two decades [4,5].

Despite only diverging a few thousand years ago [6], ONNV and CHIKV are vectored by mosquito species that diverged during the Jurassic period [7], implying that these two alphaviruses did not co-evolve with ancient mosquitoes, but are the result of cross-species transmission. CHIKV is transmitted by *Aedes* species mosquitoes of the culicine subfamily, whereas ONNV, atypically for an alphavirus, utilizes mosquitoes from the anopheline subfamily [8,9]. ONNV has been found to replicate in *Aedes aegypti* in laboratory studies, though it is not thought to be a natural vector [10]. Previous work demonstrated that nonstructural protein 3 (nsP3) is responsible for the ability of ONNV to replicate in *Anopheles* mosquitoes [11]. In that work, a recombinant CHIKV with the nsP3 of ONNV in place of the CHIKV nsP3 was able to replicate in adult anopheline mosquitoes, whereas wild-type CHIKV could not.

Alphavirus nsP3 is less well understood than other products cleaved from the viral polyprotein. It is required for genomic negative strand RNA synthesis [12,13] and it can be found in the cytoplasm and in stress granules, where it interacts with many host proteins including the mosquito stress granule protein Rasputin [14–17]. Alphavirus nsP3 is composed of three domains: the N-terminal macrodomain, the central alphavirus unique domain (AUD), and the C-terminal highly variable domain (HVD). The macrodomain hydrolyses ADP-ribose phosphate and binds polyadenylate and poly ADP-ribose [18–20]. ADP-ribosylation is used as a cellular signal in many processes, including stress granule formation, DNA repair, gene regulation, and apoptosis. The macrodomain of CHIKV nsP3 hydrolyzes mono ADP-ribose from proteins and this catalysis is important for replication in both mammalian and insect cells, as it allows nsP3 to rearrange stress granules [21–24]. The AUD is a zinc-binding domain that has been implicated in RNA binding and replication [25]. The HVD has been most studied as a protein binding domain and interacts with many host proteins [16,17]. Alphavirus nsP3 typically ends with an opal stop codon, which is read-through occasionally to produce reduced amounts of the nsP4 RNA-dependent RNA polymerase compared with the other nonstructural proteins [26]. In some ONNV strains, the opal stop codon is replaced with a codon for arginine, which affects ONNV multiplication [27,28]. The read-through products and the arginine variants add six additional amino acids (LDRAGG) to the C-terminus of nsP3 before the cleavage site that separates it from nsP4. ONNV and CHIKV nsP3 have 67% amino acid identity (75% similarity) with most of the differences falling in the HVD [29]. ONNV nsP3 is 569 amino acids long, from cleavage site to cleavage site. This is longer than the CHIKV nsP3, principally due to a 52 amino acid insertion in the HVD (S1 Fig) [29]. Despite the differences between viruses, nsP3 is a crucial determinant for alphavirus replication and pathogenesis [12,13,30–33].

ONNV is an understudied alphavirus, especially in characterizing the differences in mosquito-virus interactions that control ONNV/CHIKV species-specificity. This study identified and catalogued the anopheline proteins that differentially bound ONNV and CHIKV nsP3s to aid in understanding mosquito host factors that define specific virus infectivity.

## Materials and methods

### Cells and viruses

*Anopheles* cell lines Sua 4.0, 4a-2 [34], and MOS.55 [35] as well as Vero cells were provided by the Diagnostic Laboratory of the Arboviral Diseases Branch, Division of Vector-Borne Diseases, Centers for Disease Control and Prevention. Insect cells were maintained at 28°C in Schneider’s Drosophila Medium + 10% FBS with 100 U/mL penicillin and 100 μg/mL streptomycin (Gibco 21720–024, 15140–122, and VWR 97068–085) in sealed flasks. CHIKV strain 37997 (GenBank AY726732), ONNV strain SG650 (GenBank AF079456), and CHIKV/ONNVnsP3 were derived from plasmids pCHIK.b, pONN.AP3, and pCHIKV/ONNVnsP3 [11, 36]. In short, virus was rescued by T7 polymerase transcription and electrotransfection of Vero cells as previously described [11]. Next generation sequencing (NGS) was performed to produce sequence coverage of the CHIKV 37997, ONNV SG650, and CHIKV/ONNVnsP3 genomes from viral RNA purified from the Vero cell supernatant using the Ion Torrent Personal Genome Machine system (Life Technologies) and associated protocols [37]. In brief, viral RNA was extracted with the QIAamp viral RNA mini kit (Qiagen 52904), DNaseI treated (Invitrogen 18068015) and a cDNA library for each viral isolate was prepared using the Ovation RNA-Seq System V2 (NuGEN) and the IonExpress Plus gDNA and Amplicon Library Preparation Kit (Life Technologies) according to the manufacturer’s recommendations. Prior to shearing using the IONXpress Plus Fragment Library Kit, gDNA quantity was determined using a Qubit 2.0 Fluorometer (ThermoFisher Scientific) to optimize fragment lengths. Constructed libraries were barcoded using the Ion Xpress Barcode Adapter Kit (Life Technologies) to allow for multiplexed analysis on the Ion Torrent PGM. To assess the base-pair size profile as well as the quantity of amplified library, the sheared and purified cDNA library was analyzed on a Bioanalyzer (Agilent Technologies). The cDNA template was then diluted to the appropriate molar concentration in distilled water. To prepare and enrich template-positive particles from the cDNA library, the Ion OneTouch System Template Kit (Life Technologies) was used. The enriched ion spheres were then sequenced using the Ion Torrent Personal Genome Machine (PGM) Sequencer and the Ion Sequencing Kit, with a 318 Chip (Life Technologies). The CLC Genomics Workbench 12.0 (Qiagen) software was used to run a *de novo* assembly of the raw data reads into contigs.

### Experimental infection of cell lines

Wells of 12 well plates were seeded with 3.2 x 10^5^ cells of the three anopheline cell lines, Sua 4.0, 4a-2, or MOS.55. After plating for one hour at 28°C, the medium was removed and replaced with 400 μL medium (Schneider’s Drosophila Medium + 10% FBS with 100 U/mL penicillin and 100 μg/mL streptomycin) with 3.2 x 10^4^ pfu (MOI = 0.1) of ONNV, CHIKV, or CHIKV/ONNVnsP3. After 1h of infection, the cells were washed with Dulbecco’s PBS and 1mL of fresh medium was added. Immediately, a 200 μL aliquot was removed for the 1h timepoint and stored at -80°C. Subsequent timepoints were also 200 μL. Medium was added to replace that removed.

### Titrations

Plaque assays were performed in duplicate on Vero cell monolayers in 6 well plates with 10-fold virus dilutions to determine titer. Plates were overlayed with 0.4% agarose DMEM + 10% FBS with 100 U/mL penicillin and 100 μg/mL streptomycin and incubated for 48h at 37°C with 5% CO_2_. The cells were fixed with a 40% methanol, 0.25% crystal violet solution. Plaques were counted and are reported as pfu/mL.

### Cloning

pIE1^prm^/hr5/PA, which contains the hr5 enhancer, constitutively active strong promoter and polyadenylation signal from *Autographa californica multiple nucleopolyhedrovirus* is suitable for protein expression in many insect cells [38]. A series of PCR primers added ClaI and SalI sites to ONNV nsP3 and CHIKV nsP3 from pONN.AP3 [36] and pCHIK.b [11], as well as adding the start codon, HA tag (YPYDVPDYA) and controlling the stop codon (S1 Table and S2 and S3 Figs). Amplicons were ligated between the ClaI and SalI sites in pIE1^prm^/hr5/PA. To imitate the two C-termini produced by opal stop readthrough/arginine variant and the opal termination, CHIKV and ONNV nsP3s with either an arginine or the opal stop codon were made in case the C-terminus substantially affected binding. To add GFP, a PCR amplicon from pGFP-HA [39] was inserted into the AatII to ClaI site of the HA-nsP3. Plasmid sequences were confirmed by BigDye (ThermoFisher 4458688) Sanger sequencing on an ABI 3500xL or ABI 3130xl genetic analyzer (Applied Biosystems). Plasmids were purified by EndoFree Plasmid Maxi kit (Qiagen 12362).

### Protein expression

For transfections, 2 x 10^5^ cells were plated per cm^2^ of growth area. One day later, 0.52 μg plasmid DNA per cm^2^ and 2.08 μL/cm^2^ of FuGENE HD transfection reagent (Promega E2311) were combined per the manufacturer protocol and added to the plates (see S2 Table for details). GFP expression was verified by a Celigo Image Cytometer (Nexcelom). Two days post transfection, typically ~40% of cells were expressing GFP.

### Western blots

Transfected cells were scraped, washed twice with 0.5 mL Dulbecco’s phosphate-buffered saline (DPBS) and lysed in 1% SDS, 50 mM DTT (ThermoFisher NP0009) at 70°C for 10 min. Loading dye was added and 10 μL of sample was loaded per well. Duplicate 4–12% NuPage Bis-Tris SDS-polyacrylamide gels (ThermoFisher NP0322BOX) were run for actin and HA blots. Western blots were performed as per the manufacturer protocol using MOPS SDS buffer and PVDF (Thermofisher NP0001 and LC2005). IBlock (Thermofisher T2015) + 0.1% Tween20 was used for blocking and antibody dilution. Anti-HA (Biolegend 901513) diluted 1:1000 and anti-actin (BD Biosciences 612656) diluted 1:3333 were used. An alkaline phosphatase goat anti-mouse antibody (Jackson Immuno Research 115-055-003) was diluted 1:5000 as the secondary antibody. Blots were developed with BCIP/NBT Phosphatase Substrate System (SeraCare 5420–0030).

### Immunoprecipitation and mass spectrometry

Immunoprecipitation of GFP-HA-nsP3 proteins was carried out three days post transfection on separate pools of Sua 4.0 cells from T25 flasks expressing one of the following: non-transfected negative control, GFP-HA, GFP-HA-ONNV nsP3 opal, GFP-HA-ONNV nsP3 arg, GFP-HA-CHIKV nsP3 opal, or GFP-HA-CHIKV nsP3 arg. Manufacturer protocols were used for Pierce Crosslink Magnetic IP/Co-IP Kit (ThermoFisher 88805) with anti-GFP 12A6 (DSHB-GFP-12A6) antibody. This monoclonal antibody was developed and distributed by the Developmental Studies Hybridoma Bank, which was created by the NICHD of the NIH and maintained at The University of Iowa, Department of Biology. The lysis and wash buffer contained Halt Protease Inhibitor Cocktail (ThermoFisher 78425). The proteins of interest (GFP-HA-nsP3 variants) and bound host proteins were precipitated from lysate for 1h at room temp. All the samples were washed and were eluted with 100 μL of the HCl-containing Elution Buffer. The HCl was neutralized with 10 mL of tris-containing Neutralization Buffer from the Pierce kit. The proteins were preliminarily separated by SDS-PAGE and stained with silver stain (Pierce Silver Stain for Mass Spectrometry, 24600). Each gel lane was sliced into 12 bands and subjected to in-gel digestion with trypsin following standard protocols. In short, gel bands were destained using the Pierce silver stain kit, samples were reduced and alkylated, and digested overnight with trypsin (Promega V5111) at 37°C. The following day, tryptic peptides were extracted with three washes of 60% acetonitrile, dried in a SpeedVac, and submitted to mass spectrometry. The resulting tryptic peptides were analyzed by mass spectrometry using a Orbitrap Fusion Lumos Tribrid Mass Spectrometer (ThermoFisher Scientific) interfaced with an on-line EASY-Spray nanospray source (ThermoFisher Scientific) and a RSLCnano UPLC (ThermoFisher Scientific) configured for nanoliter per minute flows. The RSLCnano was setup with a desalting trap column (0.3 x 5 mm, 5 μm C18 PepMap 120A, ThermoFisher Scientific) and a C18 EASY-Spray analytical column (0.075 x 250 mm, 2 μm, 120A, ThermoFisher Scientific) with a column temperature of 35°C. The tryptic peptides were separated using a binary gradient of 0.1% formic acid in water (A) and 80% acetonitrile/0.1% formic acid (B). The eluent from the analytical column was introduced into the mass spectrometer using the EASY-Spray source operated at a spray voltage of 1700V with the inlet capillary temperature set to 275°C. The Lumos mass spectrometer was operated in data dependent acquisition mode with a 3 second total cycle time. The parent mass spectrum (MS) was acquired using a m/z range of 300–1500 at a resolution of 120,000 with a maximum ion injection time of 100 ms and an AGC target of 200,000 ions. The fragment mass spectrum (MS/MS) was acquired using an automated m/z range starting at 110 m/z using the “Rapid” scan mode of the ion trap with a maximum ion inject time of 300 ms and an AGC target of 3000 ions using CID fragmentation at 35% normalized collision energy with a quadrupole isolation window of 1.6 Da. The collected data was processed by Proteome Discover (v2.2.0.388, ThermoFisher Scientific) using both the built-in Sequest HT search engine and our in-house MASCOT server (v2.6, Matrix Science). The data was searched against a FASTA formatted protein list downloaded from NCBI which corresponded to the *Anopheles gambiae* species complex (NCBI:txid44542) [40]. To account for common contaminants, like human keratins, a list of common contaminating proteins were included in the search but were filtered out from the final identified protein lists. Putative names were assigned to proteins lacking descriptors using the closest clearly named homologues identified by BLAST search [29]. To better understand the immunoprecipitation results, the accession numbers of interacting proteins were run through STRING and exported to Cytoscape [41]. Protein homology between *Aedes aegypti* and *Anopheles gambiae* was calculated by BLAST when there was ≥98% coverage [29]. For these protein alignments with < 98% coverage the percent identity was manually calculated by summing BLAST-determined identical amino acids divided by the *Aedes* protein amino acid length.

## Results

**Fig 1:**
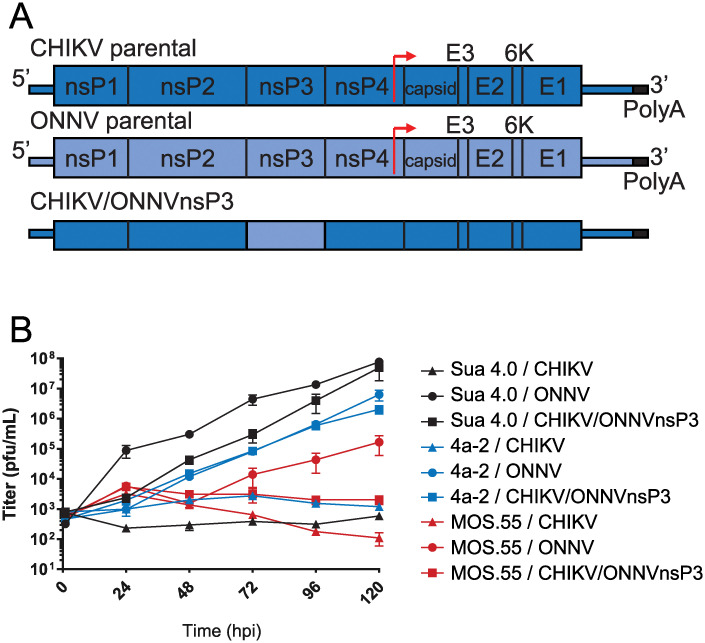
*Anopheles* cell lines infected with CHIKV, ONNV, and CHIKV/ONNVnsP3. (A) Viruses used in this study. The structures of CHIKV, ONNV, and recombinant CHIKV/ONNVnsP3 that has a CHIKV backbone with the nsP3 from ONNV are shown. Adapted from [11]. (B) Growth curves of the above viruses (MOI = 0.1) in three anopheline cell lines; Sua 4.0, 4a-2, and MOS.55. Experiment was performed in triplicate with titers assayed in duplicate for each replicate. The mean and standard error are plotted.

ONNV can be transmitted by *Anopheles* species mosquitoes, but CHIKV, its closest known relative, cannot [10,11]. Previous work had shown that CHIKV containing the nsP3 of ONNV (CHIKV/ONNVnsP3; Fig 1A) had a substantial change in vector competence allowing CHIKV/ONNVnsP3 to replicate in *Anopheles gambiae* mosquitoes, despite CHIKV genomic material comprising the majority [11]. This indicated that nsP3 was primarily responsible for alphavirus vector specificity between these two viruses. Another report identified MOS.55 anopheline cells as supporting ONNV but not CHIKV multiplication [10]. Studies were performed to determine if this difference in replication was due to nsP3, as it was in live mosquitoes. To find the best cell culture model system, two other anopheline cell lines were also tested: Sua 4.0 and 4a-2. The three anopheline cell lines Sua 4.0, MOS.55, and 4a-2, were infected with CHIKV, ONNV, or CHIKV/ONNVnsP3 at an MOI of 0.1 in triplicate with samples collected daily across five days (Fig 1B). As these viruses are non-pathogenic to mosquitoes and generally do not form plaques in insect cell monolayers, no cell death or cytopathic effects from infection were observed. Titrations on Vero cells showed that all three lines restricted CHIKV replication while allowing ONNV and CHIKV/ONNV nsP3 multiplication, though with varying efficacy. Of the three, MOS.55 cells showed the weakest phenotype, with inadequate replication in general, and so were not selected for further experimentation. Sua 4.0 cells were selected for future experiments as they best supported growth for ONNV and CHIKV/ONNVnsP3 and did not allow effective CHIKV multiplication.

**Fig 2:**
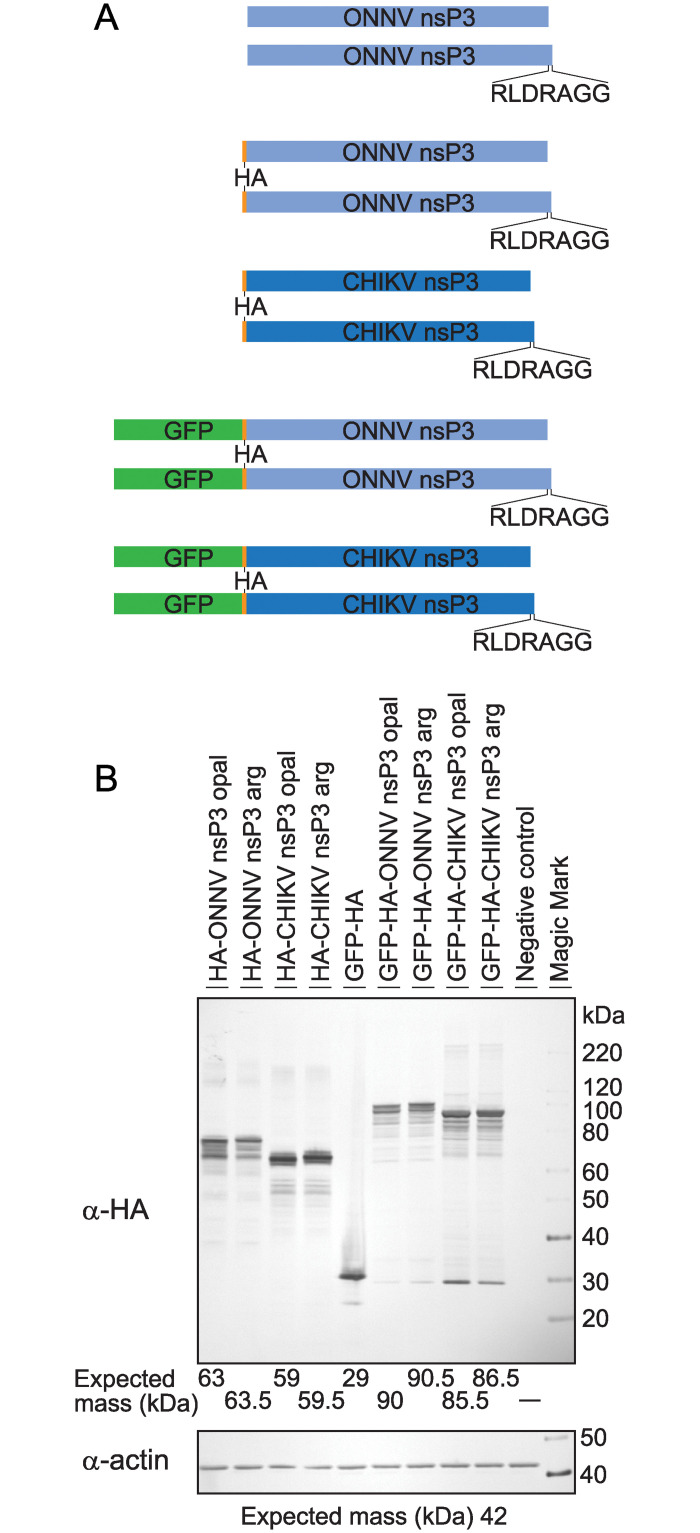
Expression of recombinant nsP3s in Sua 4.0 cells. (A) Diagrams of the protein gene cassettes used in this study that were expressed in anopheline cells. N-terminal variants were made with only the required start methionine, an HA-tag, or a GFP-HA tag. Two different C-termini were made. The shorter variants contain an opal stop codon. The longer variants have the opal codon replaced with arginine and encode seven additional amino acids to the protease cleavage site, where nsP3 is cleaved from nsP4. Recombinant nsP3 has a stop codon at the cleavage site. (B) Western blot against the HA-tag showing the alphavirus nsP3s expressed in Sua 4.0 cells. Actin was used as a loading control. The negative control cells did not have any plasmid DNA transfected.

A series of plasmids were constructed to express CHIKV and ONNV nsP3s in Sua 4.0 cells. The pIE1^prm^/hr5/PA plasmid backbone was selected [38]; this plasmid consists of a baculovirus promoter and polyadenylation signal which are suitable for robust protein expression in many insect cell lines. Three different N-termini were used. The shortest only had an artificial methionine added to start translation. An N-terminally hemagglutinin-tagged (HA; amino acids YPYDVPDYA) construct was made, as well as a N-terminally GFP-HA tagged version. Since ONNV nsP3 can naturally have either an opal stop codon at the end of nsP3 or an arginine codon that allows constitutive nsP4 translation, both versions were constructed (Fig 2A) [27,28]. The nsP3 arg contains seven amino acids (RLDRAGG) before the nsP3-nsP4 cleavage site compared with the opal variant. At the site of cleavage between nsP3 and nsP4, a stop codon was added in the plasmid. Expression was verified by western blot (Fig 2B) and by fluorescence microscopy (S4 Fig).

**Fig 3:**
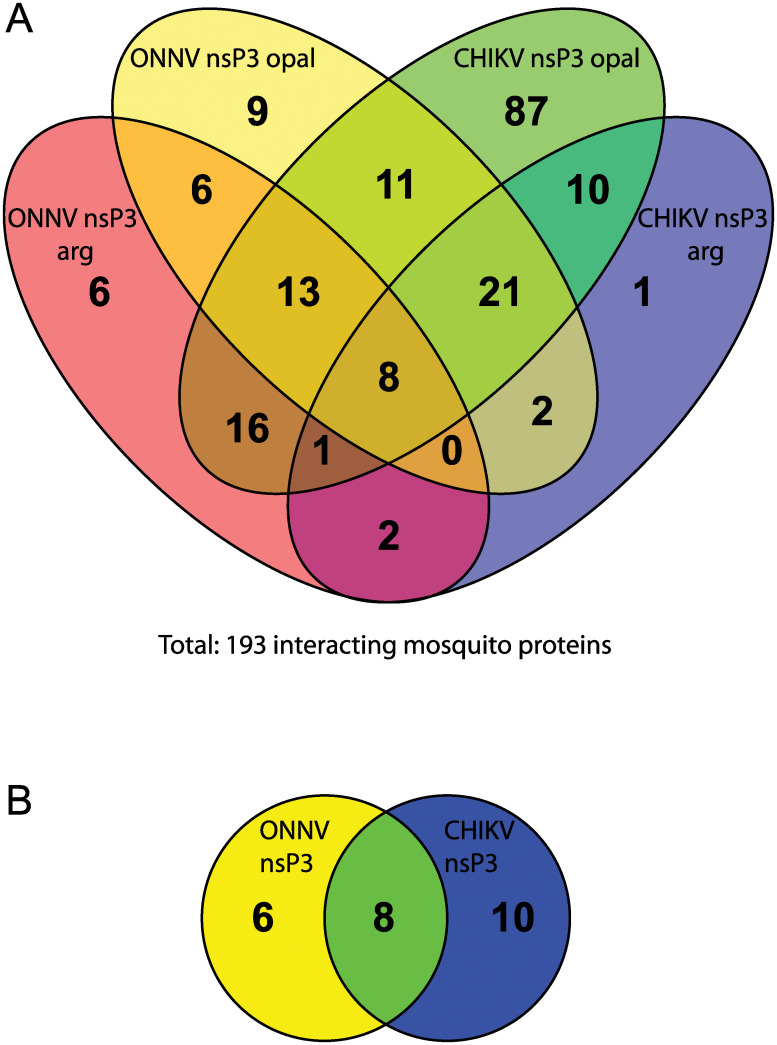
Venn diagrams of anopheline proteins that interact with ONNV nsP3 or CHIKV nsP3. GFP-HA was used to filter out proteins with non-specific binding. (A) Represents the number and overlap of proteins found in each precipitation with the GFP-HA tagged alphavirus nsP3s with either the opal stop codon, or the arginine readthrough. (B) Simplifies (A) by treating the opal and arginine variants as duplicates.

To identify the anopheline proteins that interact with nsP3, nsP3 constructs with N-terminal GFP-HA fusions were transiently expressed, immunoprecipitated with α-GFP antibody, and the proteins precipitated were identified by mass spectrometry. A western blot against the HA-tag provided relative quantitation of the precipitated bait proteins resulting in a score of 1.00 for GFP-HA-ONNV nsP3 opal, 0.74 for GFP-HA-ONNV nsP3 arg, 1.58 for GFP-HA-CHIKV nsP3 opal, and 1.55 for GFP-HA-CHIKV nsP3 arg (S5 Fig). The peptides were compared against the NCBI *gambiae* species complex protein collection (taxid 44542, approximately 53 000 proteins). GFP-HA and non-transfected cells were used as negative controls and any proteins that immunoprecipitated with GFP-HA were excluded from the analysis of the GFP-HA tagged nsP3 proteins. A total of 193 *Anopheles* proteins were identified (Fig 3A and S3 Table). CHIKV nsP3 opal had the most proteins identified, 167, CHIKV nsP3 arg had 45, ONNV nsP3 opal had 70, and ONNV nsP3 arg had 52. To identify the most promising candidates, the opal and arg variants were treated as duplicate runs to select for reliably detectible interactors. Of these, eight precipitated in the duplicate tests of both ONNV nsP3 and CHIKV nsP3 (Fig 3B). Six were consistently in both ONNV nsP3 precipitations but not in CHIKV nsP3 precipitations. Ten mosquito proteins consistently precipitated with CHIKV nsP3 but not ONNV nsP3. Additional combinations were detected (Fig 3A), but interpretation of this complicated data is less certain. In this category, eleven may be opal stop codon specific, appearing for both CHIKV and ONNV nsP3, and two might be RLDRAGG C-terminus specific. Uniprot was used to help describe the identified proteins (Table 1) [42,43]. A couple of the proteins detected, including Rasputin and coatomer subunit β, have been previously reported as interacting with CHIKV nsP3, validating the results of this approach [14,15,44,45]. Several others have been reported with other alphavirus nsP3s, but not necessarily CHIKV or ONNV nsP3 [16,17]. However, no published reports have compared the nsP3 interactomes to characterize vector-specificity of these two alphaviruses.

**Table 1:** Proteins found in duplicate nsP3 precipitations. Percent identity between the anopheline proteins and the *Aedes aegypti* homolog were provided by NCBI’s BLAST [29]. All homologs had 98% or greater query coverage, except for those marked by *. For these protein alignments with < 98% coverage the percent identity was manually calculated by summing BLAST-determined identical amino acids divided by the *Aedes* protein amino acid length.

	Putative name	NCBI Accession	Putative function	Unique peptides Identified	Exp. q-value	Uniprot ID	Identity to *Ae*. *aegypti*
ONNV nsP3 specific	WD repeat-containing protein 48 homolog (WDR48)	XP_321784.3	regulates deubiquitination	2	0	Q7PXD9	84%
	huntingtin-interacting protein (HIP1)	XP_001689077.1	clathrin-mediated endocytosis, pro-apoptotic	2	0	Q7PMU0	76%
	cathepsin L	XP_001689282.1	lysosomal protease	2	0	A7UVG1	87%
	fermitin	XP_320993.2	integrin cell adhesion	1	0.026	Q7PYQ2	87%
	breast cancer metastasis-suppressor 1-like protein	XP_312215.3	HDAC1-dependent transcriptional repression activity	1	0.033	Q7QCM6	77%
	28S ribosomal protein S22	XP_001237070.2	mitochondrial translation	1	0.043	A0NBG6	58%*
CHIKV nsP3 specific	polyadenylate-binding protein (PABP)	XP_309558.3	RNA binding and translation	1	0	Q7QH99	78%
	coatomer subunit β (COPB1)	XP_321735.4	Golgi budding for retrograde transport	4	0	Q7PXG9	89%
	coatomer subunit β’ (COPB2)	XP_318012.4	Golgi budding for retrograde transport	4	0	Q7PMU5	90%
	pyruvate dehydrogenase E1 subunit β	XP_311527.2	catalyzes pyruvate to Acetyl-CoA and CO_2_	1	0.005	Q7QDU3	91%
	40S ribosomal protein S23	XP_003435869.1	translation	1	0.005	F5HMM2	100%
	40S ribosomal protein S26	Q9GT45.2	translation	1	0.013	Q9GT45	97%
	cysteine tRNA ligase	XP_320253.2	attaches cysteine to tRNA	1	0.013	Q7Q069	80%
	replication factor C subunit 3/5	XP_314028.3	DNA repair, DNA replication	1	0.013	Q7Q9N2	85%
	replication factor C subunit 2/4	XP_312782.3	DNA repair, DNA replication	1	0.016	Q7QBM4	84%
	α-1,4 glucan phosphorylase	XP_317541.3	catalyzes phosphorolytic cleavage of glycogen	1	0.043	Q7Q3L6	93%
Common to both nsP3s	actin	XP_315269.4	cytoskeleton	15	0	Q7Q7K6	99%
	Rasputin	XP_001688309.2	stress granules, ras and Rho-mediated signaling	14	0	A7USH0	41%*
	calcium-transporting ATPase sarcoplasmic/endoplasmic reticulum type	Q7PPA5.5	calcium pump	7	0	Q7PPA5	95%
	GTP-binding nuclear protein	XP_001687858.1	nucleocytoplasmic transport	4	0	Q5TX48	98%
	cyclin-dependent kinase 1 (CDK1)	XP_307878.4	cell cycle control	2	0	Q7QKF5	90%
	60S ribosomal protein L28	XP_315433.4	translation	2	0.002	Q7PP83	61%*
	60S ribosomal protein L8	Q9U9L2.2	translation	1	0.005	Q9U9L2	93%*
	U6 snRNA phosphodiesterase	XP_307963.4	U6 snRNA maturation	1	0.012	Q7QK92	48%*

The 24 proteins found in the duplicate precipitations were subjected to STRING analysis and exported to Cytoscape [41,46]. There were no local network clusters larger than four proteins; this four protein group consisted of ribosomal proteins. The replication factor C subunits were listed in the mismatch repair, DNA replication, and nucleotide excision repair by KEGG. Overall, this approach did not reveal any larger functional patterns that would explain why these proteins might interact with nsP3. Other STRING analyses with other combinations of the precipitated proteins did not provide additional insight (S6 Fig).

## Discussion

In this study, the intracellular determinants of vector specificity for alphaviruses were investigated. To evaluate these vector-virus interactions, we utilized the close genetic relationship of ONNV and CHIKV and the wide evolutionary divergence of their mosquito hosts. Previous work indicated that the nsP3 alphavirus protein was responsible for this difference in infectivity in *Anopheles* mosquitoes [11] but the vector proteins involved in this process have not been defined. *Anopheles gambiae* Sua 4.0 cells recapitulated the *in vivo* phenotype that allows ONNV replication but restricts CHIKV. Finding this anopheline cell culture model system suggested that the restriction against CHIKV does not require the complexity of a whole mosquito and is mediated at the cellular or intracellular level. Using these cells, recombinant nsP3 proteins were expressed, immunoprecipitated, and host proteins that interact with nsp3 proteins were identified.

The search for proteins responsible for controlling alphavirus-vector specificity identified many molecules known to bind the nsP3s of other alphaviruses, or in other hosts. Known nsP3 interactors in other host species include Rasputin, polyadenylate-binding protein (PABP), WD repeat-containing protein 48 (WDR 48), and poly(ADP-ribose) polymerase (PARP) [16,17,47]. Finding these known interactors implied that the technique used was effective, and that novel interactors and differentially bound proteins are worth additional investigation. Completely novel interactors included cyclin-dependent kinase 1 (CDK1) and huntingtin-interacting protein 1 (HIP1). Few studies exist on the vector protein interactions with ONNV nsP3, let alone the determinants of vector specificity between ONNV and CHIKV due to nsP3 [15].

### Proteins precipitated by both ONNV and CHIKV nsP3

The proteins found to bind to both ONNV and CHIKV nsP3 may not be bound in the same way, or with the same affinity, and so could be the elements responsible for vector specificity. It is also possible that CHIKV nsP3 binds but fails to relocate, utilize or inactivate these proteins as required for alphavirus replication in *Anopheles* mosquitoes.

Rasputin and its mammalian homologues, Ras-GAP SH3 domain binding proteins (G3BPs), have been well established as binding alphavirus nsP3s [14,15,30,44,45,48–50]. Anopheline Rasputin (814 amino acids) was identified in both the ONNV and CHIKV nsP3 precipitations, consistent with a recent report that both ONNV and CHIKV nsP3s co-localized with *Aedes* Rasputin at similar levels [15]. The TFGDF repeats in nsP3 shown to interact with Rasputin are conserved between ONNV and CHIKV [14, 30]. Interestingly, the amino acid distance between the repeats is also conserved, but the amino acid immediately after the repeat is different and then followed by four conserved amino acids (S1 Fig). Despite the surprisingly low 41% amino acid identity between *Aedes aegypti* and *Anopheles gambiae* Rasputin proteins, the NTF2-like domain (amino acids 8–134) [51–53], which binds nsP3 [14,30], is 95% identical [29]. Additionally, Rasputin is a key RNA-binding factor in stress granules. Stress granules form when translation stalls and preinitiation mRNA-protein complexes accumulate. Stress granules either resume translation or degrade the mRNA [17]. CHIKV nsP3 seems to prevent authentic stress granule formation in mammalian cells, implying that stress granules could be anti-togaviral [23, 49, 54]. It was reported that reducing *Aedes albopictus* Rasputin levels by RNA*i* approximately doubled CHIKV titers without affecting RNA copy number *in vitro*. Curiously, reducing Rasputin levels *in vivo* decreased the CHIKV infection rate, which leaves Rasputin’s role in alphavirus multiplication unclear [14].

Actin was also found in all four nsP3 precipitations, but not in the non-transfected or GFP-HA controls, implying that this may be an authentic interaction, rather than an artifact of the precipitation. When actin has been found interacting with nsP3 in the past, it was also found in negative controls, so was discounted as an important factor [55]. This finding suggests reconsideration may be warranted. Actin is required for endocytosis of Semliki Forest virus replication spherules from the plasma membrane and nsP3 was implicated in this process [56]. However, as only 3 of the 376 amino acids are different between *Aedes* and *Anopheles* actin and it was precipitated by both nsP3s, this interaction is not likely to dictate vector specificity.

Cyclin-dependent kinase 1 (CDK1), a crucial cell cycle regulator, was found in all four precipitations, though not in the negative controls. CDK1 is downregulated by CHIKV infection [57], and nsP3 could be the culprit. The kinases responsible for phosphorylating nsP3 are unknown [58], so CDK1 is a potential candidate. There are no previous reports of nsP3 interactions with CDK1. Despite precipitating with both ONNV and CHIKV nsP3s, it is plausible that CDK1 is being utilized or inactivated by ONNV nsP3 in a way beneficial to alphavirus replication that CHIKV nsP3 cannot manage due to differences in binding orientation or localization.

### CHIKV nsP3 specific proteins

Proteins specifically bound to CHIKV nsP3 may not be relocated and released as they are by ONNV nsP3, keeping them from being utilized by the virus. Another potential mechanism is that these proteins are targeted for degradation by ONNV nsP3, but CHIKV nsP3 is not able to do that. Alternatively, CHIKV nsP3 may be bound by an antiviral anopheline protein that inhibits crucial nsP3 functions.

Polyadenylate-binding protein (PABP), a translation initiation factor commonly targeted by viruses [59], was found in the CHIKV nsP3 precipitation. PABP shares 78% identity between *Aedes aegypti* and *Anopheles gambiae*. PABP binds the polyadenylated 3’ end of mRNA and recruits the eukaryotic initiation factor 4 complex, looping the mRNA and increasing translation, while also preventing RNA degradation. It can also contribute to suppression of gene expression when stalled translation complexes are nucleated by G3BP/Rasputin into stress granules [59]. In complex with other factors, it can increase or decrease translation, protect the mRNA, or increase degradation. Human PABP was identified as binding VEEV nsP3 and is hypothesized to be important in increasing virus translation [60]. Since both ONNV and CHIKV nsP3s precipitated Rasputin, but only CHIKV nsP3 precipitated PABP, it maybe that CHIKV nsP3 is failing to disrupt or redirect constituents of the stress granules as required for alphavirus replication.

Coatomer subunit β (COPB1) and coatomer subunit β’ (COPB2) were found specifically in the CHIKV nsP3 immunoprecipitations. These two have 89% and 90% identity, respectively, between *Aedes aegypti* and *Anopheles gambiae*. These proteins are two of the seven that comprise the coat of COPI vesicles, which transport proteins retrograde from the Golgi to the endoplasmic reticulum [61]. COPB1 was previously reported to bind CHIKV nsP3 HVD in *Aedes albopictus* cells [45]. Membranes, spherules, and cellular trafficking are critical aspects of alphavirus replication and assembly complexes, so alphaviruses must manipulate some of the cellular components involved.

Poly(ADP-ribose) polymerase (PARP; NCBI XP_312938.4; Uniprot Q7QBC7), which has been shown to interact with alphavirus nsP3, signals for DNA repair, translational regulation, and cell death by adding ADP-ribose moieties to proteins [45,47,62]. PARP has been found to inhibit alphavirus replication via translational inhibition and is 70% identical in amino acid sequence between *Anopheles gambiae* and *Aedes aegypti* [63,64]. However, PARP was exclusively found to bind to the opal variant of CHIKV nsP3, and so is not on the list of the 24 most promising proteins. The macrodomain of CHIKV nsP3 may counter the function of PARP in human cells, where PARPs add ADP-ribose, and the macrodomain removes it from G3BP1 [23]. This mono ADP-ribose hydrolase activity is required for CHIKV replication in mosquito and mammalian cells [22–24]. This circumstantial evidence suggests that in anopheline cells, standard CHIKV nsP3 may be failing to counter PARP, either directly or indirectly, which could result in increased CHIKV nsP3-PARP interaction.

### ONNV nsP3 specific proteins

Proteins that coprecipitated only with ONNV nsP3 are the most likely candidates for controlling alphavirus vector specificity for anophelines. Presumably, ONNV nsP3 binds these proteins to utilize them for virus replication, or to inhibit their potential antiviral properties. Since CHIKV nsP3 does not bind these proteins, it cannot reassign them to facilitate virus multiplication.

WDR48 regulates deubiquitination, is involved in DNA damage repair, and can be manipulated by a herpesvirus protein to increase lysosome formation and degrade a cellular kinase [65,66]. It has 84% amino acid identity between the two mosquito species of interest and precipitated solely with ONNV nsP3. This is consistent with the findings of Frolov et al. 2017, in which the HVD of Sindbis virus (SINV) nsP3 precipitated WDR48 from mouse cells, but the HVD of CHIKV nsP3 did not [48]. Kim et al. 2016 similarly found WDR48 interacting with SINV nsP3 HVD in BHK cells [50]. As an important protein involved in ubiquitination-based signaling, there is potentially a wide variety of cellular changes that could be induced by manipulating this single target. Cathepsin L, a lysosomal protease, also coprecipitated with only ONNV nsP3, implicating viral manipulation of lysosomes as a potential mechanism that ONNV alters to benefit itself.

HIP1, which is involved in endocytosis, precipitated only with ONNV nsP3 [67]. During its control of clathrin bud sites, HIP1 is thought to bind actin, potentially creating a link between actin, HIP1, and nsP3 [67,68]. HIP1 has also been implicated in changes to transcription and oncogenesis and may aid coxsackievirus replication [69,70]. HIP1 has 76% identity between *Anopheles gambiae* and *Aedes aegypti*, potentially explaining the differential interaction. The implications for nsP3 manipulation of HIP1 remain unclear, but given its role in membrane rearrangements, cellular trafficking, and apoptosis, there are many possible mechanisms by which alphavirus control over this protein could benefit multiplication. HIP1 may be altering pathways that in other experimental models are influenced by amphiphysins for the benefit of alphaviruses, as both affect endocytosis and membrane trafficking. Previous reports indicate that human amphiphysins and other SH3 domain-containing proteins interact with CHIKV nsP3 via their SH3 domains [71–73]. Interestingly, we did not find anopheline amphiphysin bound to any nsP3 variant, nor any protein with SH3 domains according to the NCBI Batch Web CD-search tool [51–53]. The interaction of only ONNV nsP3 with HIP1 in anopheline cells may allow ONNV to replicate in *Anopheles* and restrict CHIKV.

### nsP3 interaction with translational components

Ribosomal proteins were found in different combinations bound to CHIKV and ONNV nsP3s. Ribosomal proteins and PABP (CHIKV nsP3 specific) imply that nsP3 influences translational regulation, presumably as part of its quest to shut down host gene expression and upregulate its own [74]. Ribosomal proteins have been identified in SINV and VEEV nsP3 precipitations in both mammalian and mosquito cells (reviewed in [16]), implying that nsP3 may recruit ribosomes to improve selective translation of alphavirus RNA. CHIKV nsP3 HVD precipitated a number of human ribosomal proteins from both the mitochondrial and cytoplasmic ribosomes [45]. However, this is unlikely to be the source of vector specificity, as ribosomal proteins were found in each condition. Of curious note, the S22 protein is a structural component of mitochondrial ribosomes, has only 58% identity between *Aedes* and *Anopheles*, and only precipitated with ONNV nsP3. It is unclear if alphaviruses utilize mitochondrial ribosomes [75], though human cytomegalovirus does [76,77].

### Limitations and conclusions

The immunoprecipitation and protein identification technique described above has several limitations. Western blots against the HA-tag common to all nsP3 constructs were performed to ensure that construct concentrations in the cells were not starkly different, but there was no method of normalizing the final vector protein results of this system. The protein identification was not quantitative, and a binary approach was used to categorize the differentially binding proteins. This did not address more subtle differences in binding kinetics, protein-interaction orientation, or binding partner functionality. Another limitation was the relatively low number of peptides identified per protein, though they still reached medium and high confidences (a false discovery rate of < 5% or < 1%, respectively). However, this does not guarantee that every interaction we found is authentic. A third limitation was that interacting proteins were likely missing due to limits of detection, being *in vitro* instead *of in vivo*, and using GFP as a filter. For example, an eIF4A-like protein (NCBI XP_318978.3; Uniprot H2KMF4) was discounted because it was found in all precipitations, including both negative controls. However, eIF4A protein is known to be recruited by VEEV nsP3 along with PABP as part of stress granule take-over, so it is not clear if ONNV and CHIKV nsP3s may have genuinely bound eIF4A [60].

The proteins that differentially interacted with ONNV or CHIKV nsP3 are strong candidates for the cellular components that affect alphavirus vector specificity. Other identified proteins, like actin, are likely not involved in determining vector specificity, but are crucial components of the alphavirus lifecycle that are manipulated by CHIKV even in the absence of successful multiplication. Many of the 24 candidates bind other viral nsP3s in other host contexts, which highlights the conservation of molecular interactions from humans to insects that arboviruses must exploit. The low homology of insect and vertebrate proteins also can stymie the infection, creating a barrier that arboviruses, uniquely, must overcome. Individual investigation of the candidates found will further reveal the intricacies of vector specificity determined by alphavirus nsP3 and lead to potential means to control pathogen transmission. For example, nsP3 interaction data has been used to design drugs that interrupt New World alphavirus nsP3-human cell interactions [47]. Understanding alphavirus-vector dynamics is a critical aspect of limiting transmission to humans.

## Supporting information

S1 Fig Alignment of CHIKV and ONNV nsP3s from BLAST. The highlighted section has been reported to interact with Rasputin/G3BP and is discussed in the text.
(TIF)

S2 Fig Annotated plasmid map of one of the constructs used for GFP-HA-ONNV nsP3 expression in anopheline cells. This is shown to orient the reader to the important components of the plasmids, including the enhancer, insect-functional promoter, poly(A) signal, origin, bacterial promoter, and ampicillin resistance gene.
(PNG)

S3 Fig Full DNA sequences of plasmids used. (DOCX)

S4 Fig Microscopy of live Sua 4.0 cells expressing GFP-HA-ONNV nsP3 opal, 24 hours post transfection. These are representative and there was no visible difference in the distribution of the different nsP3 constructs. Nuclei were stained with Hoechst 33342.
(DOCX)

S5 Fig Western blot against the HA tag of GFP-HA-nsP3s immunoprecipitated from Sua 4.0 cells. Quantitation was performed using a scanned copy of this western blot in ImageJ. The quantified bands are highlighted with a red box.
(EPS)

S6 Fig STRING network of the 193 mosquito proteins that interact with ONNV and CHIKV nsP3s visualized in Cytoscape. Manual annotation was performed to characterized groups. Proteins discussed in the text are labeled.
(PDF)

S1 Table Table of primers used. (DOCX)

S2 Table Transfection details. (DOCX)

S3 Table Table of full mass spec results—Attached as excel file. This file contains the data from mass spectrometry on the immunoprecipitated proteins in each condition (GFP-HA-ONNV nsP3 opal, GFP-HA-ONNV nsP3 arg, GFP-HA-CHIKVnsP3 opal, GFP-HA-CHIKV nsP3 arg).
(XLSX)

S1 Data Excel spreadsheet containing the underlying numerical data for Fig 1B. (XLSX)
