# Supplementary figures and images for: Identification of mosquito proteins that differentially interact with alphavirus nonstructural protein 3, a determinant of vector specificity
Source: PLoS Negl Trop Dis. 2023 Jan 25;17(1):e0011028. doi: 10.1371/journal.pntd.0011028 (PMC9876241; doi:10.1371/journal.pntd.0011028)

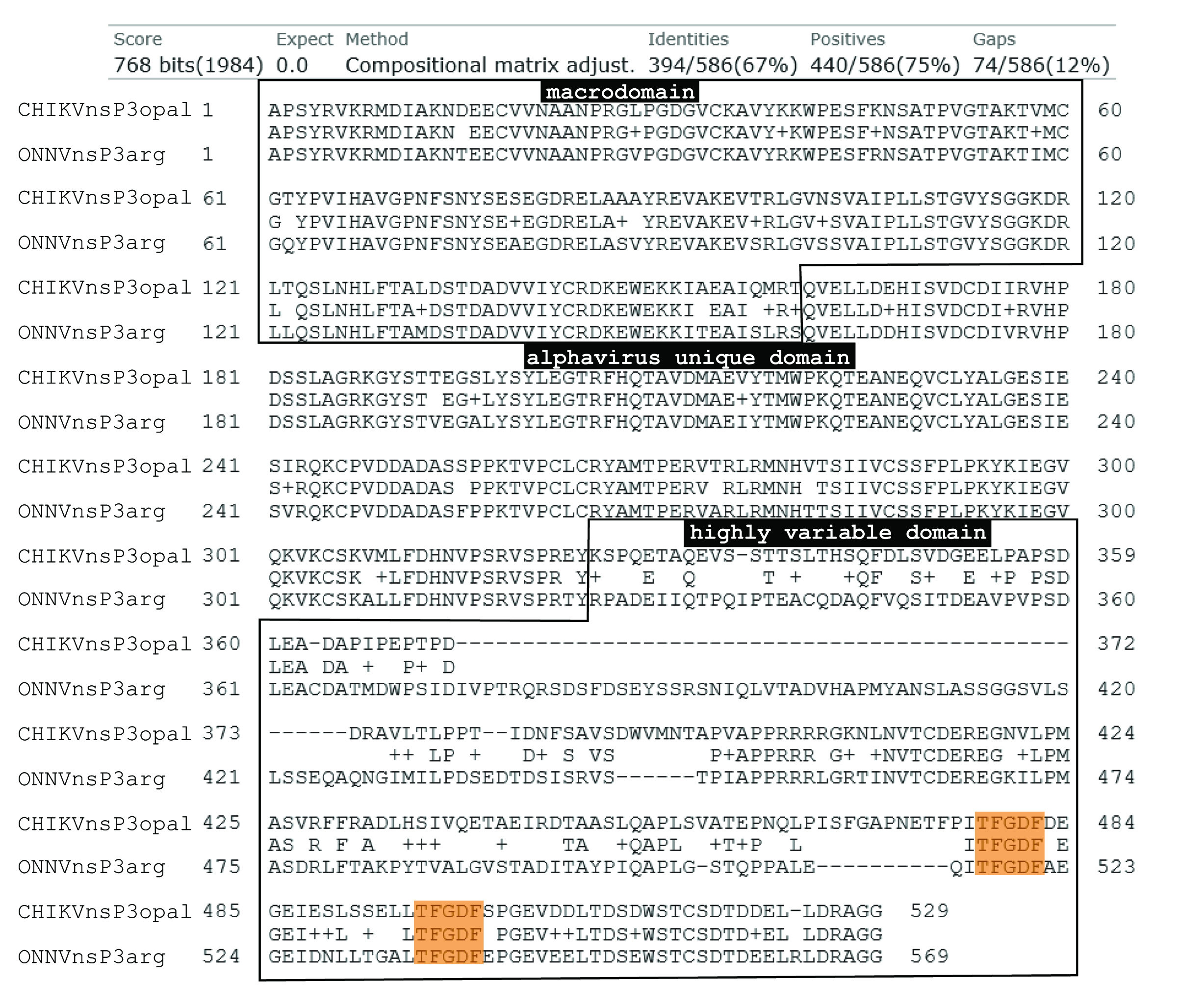

Supplement: S1 Fig — The highlighted section has been reported to interact with Rasputin/G3BP and is discussed in the text. (TIF) [file pntd.0011028.s001.tif]

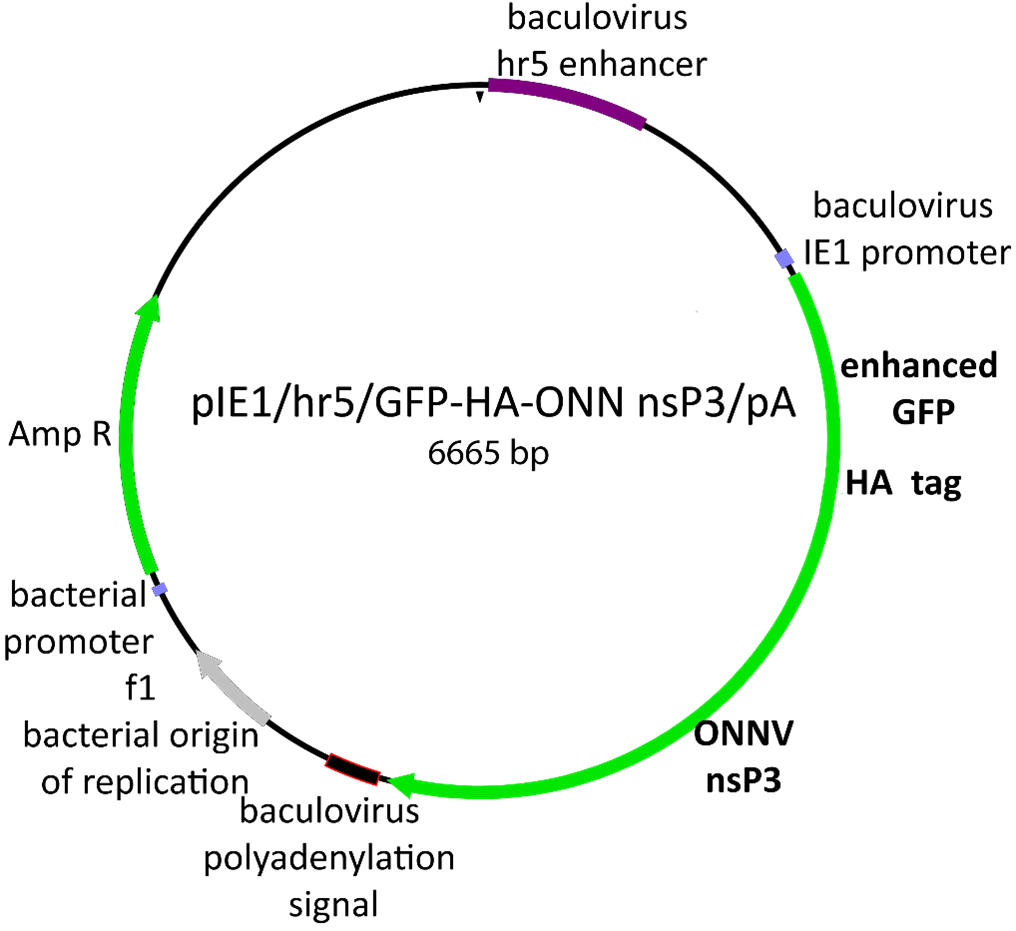

Supplement: S2 Fig — This is shown to orient the reader to the important components of the plasmids, including the enhancer, insect-functional promoter, poly(A) signal, origin, bacterial promoter, and ampicillin resistance gene. (PNG) [file pntd.0011028.s002.png]

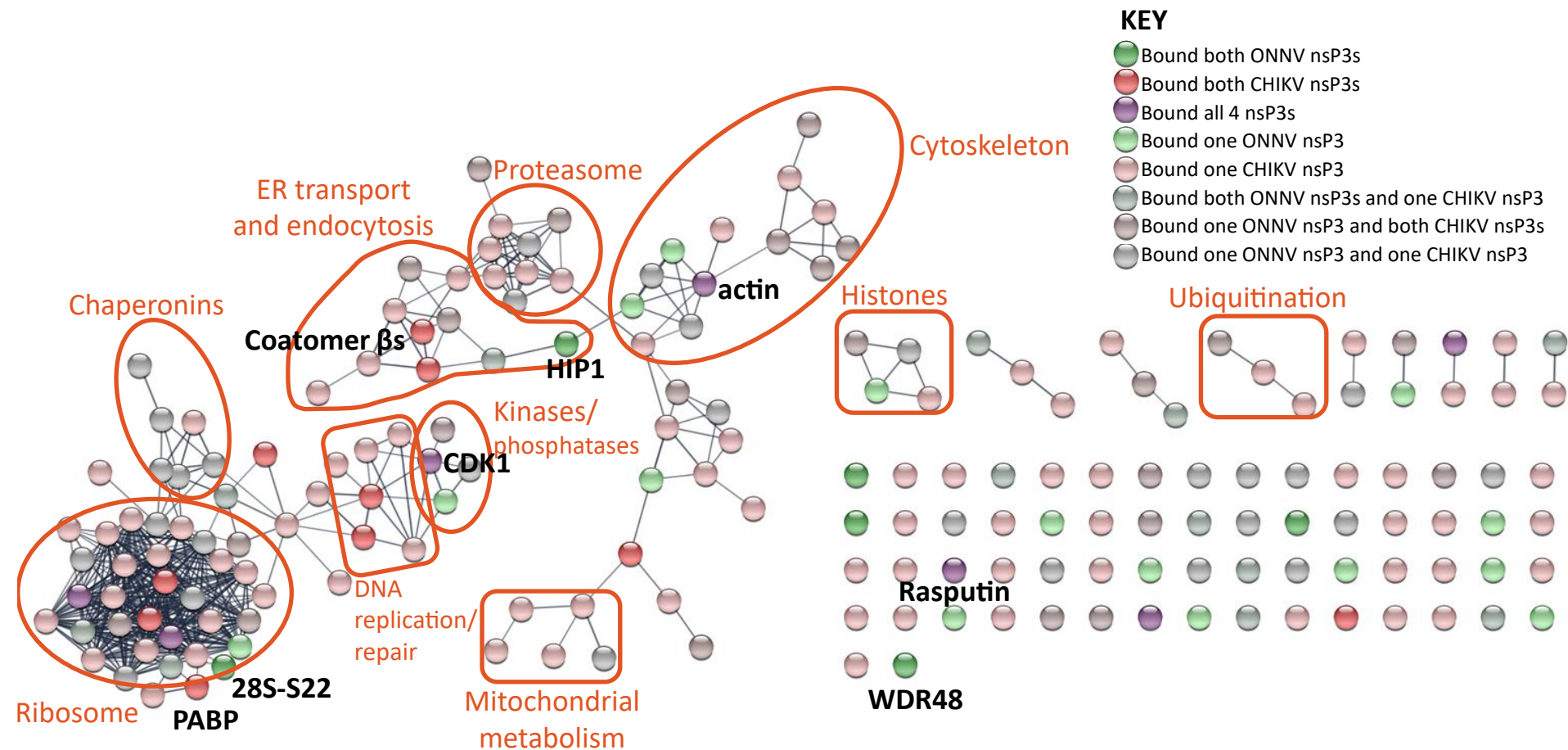

Supplement: S6 Fig — Manual annotation was performed to characterized groups. Proteins discussed in the text are labeled. (PDF) [file pntd.0011028.s006.pdf]
