# Supplementary material for: Identification of mosquito proteins that differentially interact with alphavirus nonstructural protein 3, a determinant of vector specificity
Source: PLoS Negl Trop Dis. 2023 Jan 25;17(1):e0011028. doi: 10.1371/journal.pntd.0011028 (PMC9876241; doi:10.1371/journal.pntd.0011028)
Supplement: S3 Fig — (DOCX) [file pntd.0011028.s003.docx]

S3 Fig. Full DNA sequences of plasmids used.

>pIE1-hr5-ONNnsP3 opal PA

CGGGCCCCCCCTCGAGGGGGATCCGGCGCGTAAAACACAATCAAGTATGAGTCATAAGCTGATGTCATGTTTTGCACACGGCTCATAACCGAACTGGCTTTACGAGTAGAATTCTACTTGTAACGCACGATCAGTGGATGATGGTCATTTGTTTTTCAAATCGAGATGATGTCATGTTTTGCACACGGGCTCATAAACTGCTTTACGAGTAGAATTCTACGTGTAACGCACGATCGAGATGAGTCATTTGTTTTGCAATATGATATCATACAATATGACTCATTTGTTTTTCAAAACCGAACTTGATTTACGGGTAGAATTCTACTCGTAAAGCACAATCAAAAAGATGATGTCATTTGTTTTTCAAAACTGAACTCTCGGCTTTACGAGTAGAATTCTACGTGTAAAACACAATCAAGAAATGATGTCATTTGTTATAAAAATAAAAGCTGATGTCATGTTTTGCACATGGCTCATAACTAAACTCGCTTTACGGGTAGAATTCTACGCGCCGGATCCACTAGCTAGTTCTAGAGTCGCGATGTCTTTGTGATGCGCCGACATTTTTGTAGGTTATTGATAAAATGAACGGATACAGTTGCCCGACATTATCATTAAATCCTTGGCGTAGAATTTGTCGGGTCCATTGTCCGTGTGCGCTAGCATGCCCGCTAACGGACCTCGTACTTTTGGCTTCAAAGGTTTTGCGCACAGACAAAATGTGCCACACTTGCAGCTCTGCATGTGTGCGCGTTACCACAAATCCCAACGGCGCAGTGTACTTGTTGTATGCAAATAAATCTCGATAAAGGCGCGGCGCGCGAATGCAGCTGATCACGTACGCTCCTCGTGTTCCGTTCAAGGACGGTGTTATCGACCTCAGATTAATGTTTATCGGCCGACTGTTTTCGTATCCGCTCACCAAACGCGTTTTTGCATTAACATTGTATGTCGGCGGATGTTCTATATCTAATTTGAATAAATAAACGATAACCGCGTTGGTTTTAGAGGGCATAATAAAAGAAATATTGTTATCGTGTTCGCCATTAGGGCAGTATAAATTGACGTTCATGTTGGATATTGTTTCAGTTGCAAGTTGACACTGGCGGCGACAAGAGATCTGCCGGGCTGCAGGAATTCGATATCAAGCTTATCGATGGCTCCGTCATACCGTGTGAAACGGATGGACATCGCGAAAAACACTGAGGAATGCGTGGTAAACGCCGCCAATCCACGCGGAGTACCAGGCGATGGAGTATGTAAAGCCGTGTATAGAAAATGGCCAGAATCATTCAGAAACAGTGCAACGCCAGTGGGTACTGCAAAGACAATCATGTGCGGTCAATACCCCGTCATCCACGCGGTAGGCCCAAACTTCTCAAACTATTCTGAGGCTGAAGGGGATAGGGAATTGGCTTCAGTGTATAGAGAAGTGGCGAAAGAAGTGTCTAGGCTAGGAGTGAGCAGTGTAGCCATCCCTTTGCTTTCAACCGGTGTGTACTCAGGAGGCAAAGATAGACTGCTGCAATCACTAAACCATCTTTTCACAGCGATGGATTCGACAGATGCAGATGTTGTCATCTATTGCAGGGATAAGGAATGGGAGAAGAAGATCACTGAAGCCATATCATTAAGATCCCAGGTAGAACTACTAGATGATCACATCTCAGTGGATTGCGACATTGTACGCGTTCATCCAGACAGCAGCTTGGCAGGCCGAAAGGGGTACAGCACAGTAGAGGGAGCACTCTACTCGTACCTAGAGGGAACAAGATTCCACCAAACTGCTGTAGATATGGCAGAAATATATACCATGTGGCCAAAACAAACTGAAGCCAATGAACAGGTCTGCCTATATGCTCTGGGGGAGAGCATAGAGTCAGTCAGGCAAAAATGTCCTGTAGACGACGCCGACGCCTCATTCCCTCCGAAAACAGTCCCGTGCCTATGCCGTTATGCCATGACGCCTGAACGAGTTGCACGCCTACGCATGAATCATACTACTAGCATCATAGTGTGCTCGTCTTTTCCACTGCCGAAGTACAAAATCGAGGGCGTGCAAAAAGTAAAATGTTCAAAAGCACTCTTGTTTGATCACAACGTACCGTCTCGAGTGAGCCCGAGAACGTACAGGCCTGCGGACGAAATCATACAGACACCTCAAATACCAACTGAAGCGTGCCAGGACGCACAATTCGTGCAGTCAATAACTGATGAAGCAGTGCCAGTTCCGTCAGACTTAGAGGCTTGTGACGCAACTATGGACTGGCCCTCTATCGACATCGTACCAACAAGACAAAGAAGCGACTCATTTGACAGCGAGTATAGTTCCAGAAGTAACATACAGCTGGTGACAGCGGACGTGCATGCACCAATGTACGCAAATTCGCTGGCGTCCAGCGGAGGTTCAGTGCTGTCGCTGTCCAGTGAACAAGCTCAGAACGGCATAATGATACTACCTGATTCAGAAGACACAGATAGTATAAGCAGAGTAAGCACACCGATCGCCCCACCCAGGAGACGTTTGGGAAGGACTATAAATGTGACTTGTGACGAGCGGGAAGGGAAAATACTCCCTATGGCCAGCGACAGGCTCTTCACTGCTAAGCCATACACTGTCGCACTGGGCGTATCAACAGCAGACATAACTGCGTACCCCATCCAGGCACCGCTAGGATCGACACAACCGCCTGCCCTCGAACAGATCACTTTCGGAGATTTCGCCGAAGGTGAAATAGACAACCTCCTGACAGGGGCATTGACATTTGGAGACTTCGAGCCAGGTGAAGTGGAAGAGCTGACGGATAGCGAGTGGTCAACATGCTCGGACACAGATGAAGAGTTATGAGTCGACCTCGACTAGTGCACTCAACAAAAATGTAATATTAAACACAATTAAATAAATGTTAAAATTTATTGCCTAATATTATTTTGTCATTGCTTGTCATTTATTAATTTGGATGATGTCATTTGTTTTTAAAATTGAACTGGCTTTACGAGTAGAATTCGAGCTCCAATTCGCCCTATAGTGAGTCGTATTACAATTCACTGGCCGTCGTTTTACAACGTCGTGACTGGGAAAACCCTGGCGTTACCCAACTTAATCGCCTTGCAGCACATCCCCCTTTCGCCAGCTGGCGTAATAGCGAAGAGGCCCGCACCGATCGCCCTTCCCAACAGTTGCGCAGCCTGAATGGCGAATGGCGCGACGCGCCCTGTAGCGGCGCATTAAGCGCGGCGGGTGTGGTGGTTACGCGCAGCGTGACCGCTACACTTGCCAGCGCCCTAGCGCCCGCTCCTTTCGCTTTCTTCCCTTCCTTTCTCGCCACGTTCGCCGGCTTTCCCCGTCAAGCTCTAAATCGGGGGCTCCCTTTAGGGTTCCGATTTAGTGCTTTACGGCACCTCGACCCCAAAAAACTTGATTAGGGTGATGGTTCACGTAGTGGGCCATCGCCCTGATAGACGGTTTTTCGCCCTTTGACGTTGGAGTCCACGTTCTTTAATAGTGGACTCTTGTTCCAAACTGGAACAACACTCAACCCTATCTCGGTCTATTCTTTTGATTTATAAGGGATTTTGCCGATTTCGGCCTATTGGTTAAAAAATGAGCTGATTTAACAAAAATTTAACGCGAATTTTAACAAAATATTAACGTTTACAATTTCCCAGGTGGCACTTTTCGGGGAAATGTGCGCGGAACCCCTATTTGTTTATTTTTCTAAATACATTCAAATATGTATCCGCTCATGAGACAATAACCCTGATAAATGCTTCAATAATATTGAAAAAGGAAGAGTATGAGTATTCAACATTTCCGTGTCGCCCTTATTCCCTTTTTTGCGGCATTTTGCCTTCCTGTTTTTGCTCACCCAGAAACGCTGGTGAAAGTAAAAGATGCTGAAGATCAGTTGGGTGCACGAGTGGGTTACATCGAACTGGATCTCAACAGCGGTAAGATCCTTGAGAGTTTTCGCCCCGAAGAACGTTTTCCAATGATGAGCACTTTTAAAGTTCTGCTATGTGGCGCGGTATTATCCCGTATTGACGCCGGGCAAGAGCAACTCGGTCGCCGCATACACTATTCTCAGAATGACTTGGTTGAGTACTCACCAGTCACAGAAAAGCATCTTACGGATGGCATGACAGTAAGAGAATTATGCAGTGCTGCCATAACCATGAGTGATAACACTGCGGCCAACTTACTTCTGACAACGATCGGAGGACCGAAGGAGCTAACCGCTTTTTTGCACAACATGGGGGATCATGTAACTCGCCTTGATCGTTGGGAACCGGAGCTGAATGAAGCCATACCAAACGACGAGCGTGACACCACGATGCCTGTAGCAATGGCAACAACGTTGCGCAAACTATTAACTGGCGAACTACTTACTCTAGCTTCCCGGCAACAATTAATAGACTGGATGGAGGCGGATAAAGTTGCAGGACCACTTCTGCGCTCGGCCCTTCCGGCTGGCTGGTTTATTGCTGATAAATCTGGAGCCGGTGAGCGTGGGTCTCGCGGTATCATTGCAGCACTGGGGCCAGATGGTAAGCCCTCCCGTATCGTAGTTATCTACACGACGGGGAGTCAGGCAACTATGGATGAACGAAATAGACAGATCGCTGAGATAGGTGCCTCACTGATTAAGCATTGGTAACTGTCAGACCAAGTTTACTCATATATACTTTAGATTGATTTAAAACTTCATTTTTAATTTAAAAGGATCTAGGTGAAGATCCTTTTTGATAATCTCATGACCAAAATCCCTTAACGTGAGTTTTCGTTCCACTGAGCGTCAGACCCCGTAGAAAAGATCAAAGGATCTTCTTGAGATCCTTTTTTTCTGCGCGTAATCTGCTGCTTGCAAACAAAAAAACCACCGCTACCAGCGGTGGTTTGTTTGCCGGATCAAGAGCTACCAACTCTTTTTCCGAAGGTAACTGGCTTCAGCAGAGCGCAGATACCAAATACTGTCCTTCTAGTGTAGCCGTAGTTAGGCCACCACTTCAAGAACTCTGTAGCACCGCCTACATACCTCGCTCTGCTAATCCTGTTACCAGTGGCTGCTGCCAGTGGCGATAAGTCGTGTCTTACCGGGTTGGACTCAAGACGATAGTTACCGGATAAGGCGCAGCGGTCGGGCTGAACGGGGGGTTCGTGCACACAGCCCAGCTTGGAGCGAACGACCTACACCGAACTGAGATACCTACAGCGTGAGCTATGAGAAAGCGCCACGCTTCCCGAAGGGAGAAAGGCGGACAGGTATCCGGTAAGCGGCAGGGTCGGAACAGGAGAGCGCACGAGGGAGCTTCCAGGGGGAAACGCCTGGTATCTTTATAGTCCTGTCGGGTTTCGCCACCTCTGACTTGAGCGTCGATTTTTGTGATGCTCGTCAGGGGGGCGGAGCCTATGGAAAAACGCCAGCAACGCGGCCTTTTTACGGTTCCTGGCCTTTTGCTGGCCTTTTGCTCACATGTTCTTTCCTGCGTTATCCCCTGATTCTGTGGATAACCGTATTACCGCCTTTGAGTGAGCTGATACCGCTCGCCGCAGCCGAACGACCGAGCGCAGCGAGTCAGTGAGCGAGGAAGCGGAAGAGCGCCCAATACGCAAACCGCCTCTCCCCGCGCGTTGGCCGATTCATTAATGCAGCTGGCACGACAGGTTTCCCGACTGGAAAGCGGGCAGTGAGCGCAACGCAATTAATGTGAGTTAGCTCACTCATTAGGCACCCCAGGCTTTACACTTTATGCTTCCGGCTCGTATGTTGTGTGGAATTGTGAGCGGATAACAATTTCACACAGGAAACAGCTATGACCATGATTACGCCAAGCTCGGAATTAACCCTCACTAAAGGGAACAAAAGCTGGGTAC

>pIE1-hr5-ONNnsP3 arg PA

CGGGCCCCCCCTCGAGGGGGATCCGGCGCGTAAAACACAATCAAGTATGAGTCATAAGCTGATGTCATGTTTTGCACACGGCTCATAACCGAACTGGCTTTACGAGTAGAATTCTACTTGTAACGCACGATCAGTGGATGATGGTCATTTGTTTTTCAAATCGAGATGATGTCATGTTTTGCACACGGGCTCATAAACTGCTTTACGAGTAGAATTCTACGTGTAACGCACGATCGAGATGAGTCATTTGTTTTGCAATATGATATCATACAATATGACTCATTTGTTTTTCAAAACCGAACTTGATTTACGGGTAGAATTCTACTCGTAAAGCACAATCAAAAAGATGATGTCATTTGTTTTTCAAAACTGAACTCTCGGCTTTACGAGTAGAATTCTACGTGTAAAACACAATCAAGAAATGATGTCATTTGTTATAAAAATAAAAGCTGATGTCATGTTTTGCACATGGCTCATAACTAAACTCGCTTTACGGGTAGAATTCTACGCGCCGGATCCACTAGCTAGTTCTAGAGTCGCGATGTCTTTGTGATGCGCCGACATTTTTGTAGGTTATTGATAAAATGAACGGATACAGTTGCCCGACATTATCATTAAATCCTTGGCGTAGAATTTGTCGGGTCCATTGTCCGTGTGCGCTAGCATGCCCGCTAACGGACCTCGTACTTTTGGCTTCAAAGGTTTTGCGCACAGACAAAATGTGCCACACTTGCAGCTCTGCATGTGTGCGCGTTACCACAAATCCCAACGGCGCAGTGTACTTGTTGTATGCAAATAAATCTCGATAAAGGCGCGGCGCGCGAATGCAGCTGATCACGTACGCTCCTCGTGTTCCGTTCAAGGACGGTGTTATCGACCTCAGATTAATGTTTATCGGCCGACTGTTTTCGTATCCGCTCACCAAACGCGTTTTTGCATTAACATTGTATGTCGGCGGATGTTCTATATCTAATTTGAATAAATAAACGATAACCGCGTTGGTTTTAGAGGGCATAATAAAAGAAATATTGTTATCGTGTTCGCCATTAGGGCAGTATAAATTGACGTTCATGTTGGATATTGTTTCAGTTGCAAGTTGACACTGGCGGCGACAAGAGATCTGCCGGGCTGCAGGAATTCGATATCAAGCTTATCGATGGCTCCGTCATACCGTGTGAAACGGATGGACATCGCGAAAAACACTGAGGAATGCGTGGTAAACGCCGCCAATCCACGCGGAGTACCAGGCGATGGAGTATGTAAAGCCGTGTATAGAAAATGGCCAGAATCATTCAGAAACAGTGCAACGCCAGTGGGTACTGCAAAGACAATCATGTGCGGTCAATACCCCGTCATCCACGCGGTAGGCCCAAACTTCTCAAACTATTCTGAGGCTGAAGGGGATAGGGAATTGGCTTCAGTGTATAGAGAAGTGGCGAAAGAAGTGTCTAGGCTAGGAGTGAGCAGTGTAGCCATCCCTTTGCTTTCAACCGGTGTGTACTCAGGAGGCAAAGATAGACTGCTGCAATCACTAAACCATCTTTTCACAGCGATGGATTCGACAGATGCAGATGTTGTCATCTATTGCAGGGATAAGGAATGGGAGAAGAAGATCACTGAAGCCATATCATTAAGATCCCAGGTAGAACTACTAGATGATCACATCTCAGTGGATTGCGACATTGTACGCGTTCATCCAGACAGCAGCTTGGCAGGCCGAAAGGGGTACAGCACAGTAGAGGGAGCACTCTACTCGTACCTAGAGGGAACAAGATTCCACCAAACTGCTGTAGATATGGCAGAAATATATACCATGTGGCCAAAACAAACTGAAGCCAATGAACAGGTCTGCCTATATGCTCTGGGGGAGAGCATAGAGTCAGTCAGGCAAAAATGTCCTGTAGACGACGCCGACGCCTCATTCCCTCCGAAAACAGTCCCGTGCCTATGCCGTTATGCCATGACGCCTGAACGAGTTGCACGCCTACGCATGAATCATACTACTAGCATCATAGTGTGCTCGTCTTTTCCACTGCCGAAGTACAAAATCGAGGGCGTGCAAAAAGTAAAATGTTCAAAAGCACTCTTGTTTGATCACAACGTACCGTCTCGAGTGAGCCCGAGAACGTACAGGCCTGCGGACGAAATCATACAGACACCTCAAATACCAACTGAAGCGTGCCAGGACGCACAATTCGTGCAGTCAATAACTGATGAAGCAGTGCCAGTTCCGTCAGACTTAGAGGCTTGTGACGCAACTATGGACTGGCCCTCTATCGACATCGTACCAACAAGACAAAGAAGCGACTCATTTGACAGCGAGTATAGTTCCAGAAGTAACATACAGCTGGTGACAGCGGACGTGCATGCACCAATGTACGCAAATTCGCTGGCGTCCAGCGGAGGTTCAGTGCTGTCGCTGTCCAGTGAACAAGCTCAGAACGGCATAATGATACTACCTGATTCAGAAGACACAGATAGTATAAGCAGAGTAAGCACACCGATCGCCCCACCCAGGAGACGTTTGGGAAGGACTATAAATGTGACTTGTGACGAGCGGGAAGGGAAAATACTCCCTATGGCCAGCGACAGGCTCTTCACTGCTAAGCCATACACTGTCGCACTGGGCGTATCAACAGCAGACATAACTGCGTACCCCATCCAGGCACCGCTAGGATCGACACAACCGCCTGCCCTCGAACAGATCACTTTCGGAGATTTCGCCGAAGGTGAAATAGACAACCTCCTGACAGGGGCATTGACATTTGGAGACTTCGAGCCAGGTGAAGTGGAAGAGCTGACGGATAGCGAGTGGTCAACATGCTCGGACACAGATGAAGAGTTACGACTAGACAGAGCAGGGGGTTAGATATTCTCCTCTGACACTGGTCGTCGACCTCGACTAGTGCACTCAACAAAAATGTAATATTAAACACAATTAAATAAATGTTAAAATTTATTGCCTAATATTATTTTGTCATTGCTTGTCATTTATTAATTTGGATGATGTCATTTGTTTTTAAAATTGAACTGGCTTTACGAGTAGAATTCGAGCTCCAATTCGCCCTATAGTGAGTCGTATTACAATTCACTGGCCGTCGTTTTACAACGTCGTGACTGGGAAAACCCTGGCGTTACCCAACTTAATCGCCTTGCAGCACATCCCCCTTTCGCCAGCTGGCGTAATAGCGAAGAGGCCCGCACCGATCGCCCTTCCCAACAGTTGCGCAGCCTGAATGGCGAATGGCGCGACGCGCCCTGTAGCGGCGCATTAAGCGCGGCGGGTGTGGTGGTTACGCGCAGCGTGACCGCTACACTTGCCAGCGCCCTAGCGCCCGCTCCTTTCGCTTTCTTCCCTTCCTTTCTCGCCACGTTCGCCGGCTTTCCCCGTCAAGCTCTAAATCGGGGGCTCCCTTTAGGGTTCCGATTTAGTGCTTTACGGCACCTCGACCCCAAAAAACTTGATTAGGGTGATGGTTCACGTAGTGGGCCATCGCCCTGATAGACGGTTTTTCGCCCTTTGACGTTGGAGTCCACGTTCTTTAATAGTGGACTCTTGTTCCAAACTGGAACAACACTCAACCCTATCTCGGTCTATTCTTTTGATTTATAAGGGATTTTGCCGATTTCGGCCTATTGGTTAAAAAATGAGCTGATTTAACAAAAATTTAACGCGAATTTTAACAAAATATTAACGTTTACAATTTCCCAGGTGGCACTTTTCGGGGAAATGTGCGCGGAACCCCTATTTGTTTATTTTTCTAAATACATTCAAATATGTATCCGCTCATGAGACAATAACCCTGATAAATGCTTCAATAATATTGAAAAAGGAAGAGTATGAGTATTCAACATTTCCGTGTCGCCCTTATTCCCTTTTTTGCGGCATTTTGCCTTCCTGTTTTTGCTCACCCAGAAACGCTGGTGAAAGTAAAAGATGCTGAAGATCAGTTGGGTGCACGAGTGGGTTACATCGAACTGGATCTCAACAGCGGTAAGATCCTTGAGAGTTTTCGCCCCGAAGAACGTTTTCCAATGATGAGCACTTTTAAAGTTCTGCTATGTGGCGCGGTATTATCCCGTATTGACGCCGGGCAAGAGCAACTCGGTCGCCGCATACACTATTCTCAGAATGACTTGGTTGAGTACTCACCAGTCACAGAAAAGCATCTTACGGATGGCATGACAGTAAGAGAATTATGCAGTGCTGCCATAACCATGAGTGATAACACTGCGGCCAACTTACTTCTGACAACGATCGGAGGACCGAAGGAGCTAACCGCTTTTTTGCACAACATGGGGGATCATGTAACTCGCCTTGATCGTTGGGAACCGGAGCTGAATGAAGCCATACCAAACGACGAGCGTGACACCACGATGCCTGTAGCAATGGCAACAACGTTGCGCAAACTATTAACTGGCGAACTACTTACTCTAGCTTCCCGGCAACAATTAATAGACTGGATGGAGGCGGATAAAGTTGCAGGACCACTTCTGCGCTCGGCCCTTCCGGCTGGCTGGTTTATTGCTGATAAATCTGGAGCCGGTGAGCGTGGGTCTCGCGGTATCATTGCAGCACTGGGGCCAGATGGTAAGCCCTCCCGTATCGTAGTTATCTACACGACGGGGAGTCAGGCAACTATGGATGAACGAAATAGACAGATCGCTGAGATAGGTGCCTCACTGATTAAGCATTGGTAACTGTCAGACCAAGTTTACTCATATATACTTTAGATTGATTTAAAACTTCATTTTTAATTTAAAAGGATCTAGGTGAAGATCCTTTTTGATAATCTCATGACCAAAATCCCTTAACGTGAGTTTTCGTTCCACTGAGCGTCAGACCCCGTAGAAAAGATCAAAGGATCTTCTTGAGATCCTTTTTTTCTGCGCGTAATCTGCTGCTTGCAAACAAAAAAACCACCGCTACCAGCGGTGGTTTGTTTGCCGGATCAAGAGCTACCAACTCTTTTTCCGAAGGTAACTGGCTTCAGCAGAGCGCAGATACCAAATACTGTCCTTCTAGTGTAGCCGTAGTTAGGCCACCACTTCAAGAACTCTGTAGCACCGCCTACATACCTCGCTCTGCTAATCCTGTTACCAGTGGCTGCTGCCAGTGGCGATAAGTCGTGTCTTACCGGGTTGGACTCAAGACGATAGTTACCGGATAAGGCGCAGCGGTCGGGCTGAACGGGGGGTTCGTGCACACAGCCCAGCTTGGAGCGAACGACCTACACCGAACTGAGATACCTACAGCGTGAGCTATGAGAAAGCGCCACGCTTCCCGAAGGGAGAAAGGCGGACAGGTATCCGGTAAGCGGCAGGGTCGGAACAGGAGAGCGCACGAGGGAGCTTCCAGGGGGAAACGCCTGGTATCTTTATAGTCCTGTCGGGTTTCGCCACCTCTGACTTGAGCGTCGATTTTTGTGATGCTCGTCAGGGGGGCGGAGCCTATGGAAAAACGCCAGCAACGCGGCCTTTTTACGGTTCCTGGCCTTTTGCTGGCCTTTTGCTCACATGTTCTTTCCTGCGTTATCCCCTGATTCTGTGGATAACCGTATTACCGCCTTTGAGTGAGCTGATACCGCTCGCCGCAGCCGAACGACCGAGCGCAGCGAGTCAGTGAGCGAGGAAGCGGAAGAGCGCCCAATACGCAAACCGCCTCTCCCCGCGCGTTGGCCGATTCATTAATGCAGCTGGCACGACAGGTTTCCCGACTGGAAAGCGGGCAGTGAGCGCAACGCAATTAATGTGAGTTAGCTCACTCATTAGGCACCCCAGGCTTTACACTTTATGCTTCCGGCTCGTATGTTGTGTGGAATTGTGAGCGGATAACAATTTCACACAGGAAACAGCTATGACCATGATTACGCCAAGCTCGGAATTAACCCTCACTAAAGGGAACAAAAGCTGGGTAC

>pIE1-hr5-HA-CHIKnsP3 opal PA

CGGGCCCCCCCTCGAGGGGGATCCGGCGCGTAAAACACAATCAAGTATGAGTCATAAGCTGATGTCATGTTTTGCACACGGCTCATAACCGAACTGGCTTTACGAGTAGAATTCTACTTGTAACGCACGATCAGTGGATGATGGTCATTTGTTTTTCAAATCGAGATGATGTCATGTTTTGCACACGGGCTCATAAACTGCTTTACGAGTAGAATTCTACGTGTAACGCACGATCGAGATGAGTCATTTGTTTTGCAATATGATATCATACAATATGACTCATTTGTTTTTCAAAACCGAACTTGATTTACGGGTAGAATTCTACTCGTAAAGCACAATCAAAAAGATGATGTCATTTGTTTTTCAAAACTGAACTCTCGGCTTTACGAGTAGAATTCTACGTGTAAAACACAATCAAGAAATGATGTCATTTGTTATAAAAATAAAAGCTGATGTCATGTTTTGCACATGGCTCATAACTAAACTCGCTTTACGGGTAGAATTCTACGCGCCGGATCCACTAGCTAGTTCTAGAGTCGCGATGTCTTTGTGATGCGCCGACATTTTTGTAGGTTATTGATAAAATGAACGGATACAGTTGCCCGACATTATCATTAAATCCTTGGCGTAGAATTTGTCGGGTCCATTGTCCGTGTGCGCTAGCATGCCCGCTAACGGACCTCGTACTTTTGGCTTCAAAGGTTTTGCGCACAGACAAAATGTGCCACACTTGCAGCTCTGCATGTGTGCGCGTTACCACAAATCCCAACGGCGCAGTGTACTTGTTGTATGCAAATAAATCTCGATAAAGGCGCGGCGCGCGAATGCAGCTGATCACGTACGCTCCTCGTGTTCCGTTCAAGGACGGTGTTATCGACCTCAGATTAATGTTTATCGGCCGACTGTTTTCGTATCCGCTCACCAAACGCGTTTTTGCATTAACATTGTATGTCGGCGGATGTTCTATATCTAATTTGAATAAATAAACGATAACCGCGTTGGTTTTAGAGGGCATAATAAAAGAAATATTGTTATCGTGTTCGCCATTAGGGCAGTATAAATTGACGTTCATGTTGGATATTGTTTCAGTTGCAAGTTGACACTGGCGGCGACAAGAGATCTGCCGGGCTGCAGGAATTCGATATCAAGCTTATCGATGTACCCATACGACGTCCCAGACTACGCACCGTCGTACCGGGTTAAACGCATGGACATCGCAAAGAACGATGAAGAGTGTGTAGTCAACGCCGCCAACCCTCGTGGGCTACCAGGCGATGGCGTCTGTAAAGCAGTATACAAAAAATGGCCGGAGTCCTTCAAGAACAGTGCAACACCAGTGGGAACCGCAAAGACAGTCATGTGCGGTACATACCCGGTAATCCATGCAGTAGGACCTAATTTCTCAAATTACTCTGAGTCCGAAGGAGACCGGGAATTGGCAGCTGCTTACCGAGAAGTCGCTAAGGAGGTGACTAGACTAGGAGTAAACAGCGTAGCTATACCGCTCCTTTCCACCGGTGTGTACTCTGGAGGGAAAGACAGGCTGACTCAGTCACTAAACCACCTTTTTACAGCATTAGACTCAACTGATGCAGATGTGGTTATCTACTGCCGCGACAAGGAGTGGGAGAAGAAAATAGCTGAGGCCATACAAATGAGGACCCAAGTGGAATTACTAGACGAACACATCTCTGTAGACTGCGATATCATCCGAGTGCACCCTGACAGCAGTTTGGCAGGTAGAAAAGGGTACAGCACTACAGAAGGTTCACTGTACTCCTACTTGGAAGGGACACGGTTCCATCAGACGGCAGTGGACATGGCAGAAGTATACACCATGTGGCCAAAGCAGACGGAGGCTAATGAACAAGTTTGCTTGTACGCATTGGGGGAAAGTATAGAATCAATCAGGCAAAAGTGCCCAGTGGATGACGCAGATGCATCGTCGCCCCCAAAAACCGTCCCGTGCCTCTGCCGTTATGCCATGACACCCGAACGAGTCACCAGGCTTCGTATGAACCATGTCACAAGCATAATAGTATGCTCATCATTCCCCCTTCCAAAGTATAAAATAGAAGGAGTGCAGAAAGTCAAGTGTTCTAAAGTGATGCTGTTCGACCATAACGTGCCATCACGCGTTAGTCCAAGGGAATATAAATCGCCTCAGGAGACCGCACAAGAAGTAAGTTCGACCACGTCACTGACGCACAGCCAATTCGACCTTAGCGTTGACGGTGAGGAACTGCCCGCTCCGTCTGACTTGGAAGCTGACGCTCCGATTCCGGAACCAACACCAGACGACAGAGCGGTACTTACTTTGCCTCCCACGATTGATAATTTTTCGGCTGTGTCAGACTGGGTAATGAATACCGCGCCAGTCGCACCACCCAGAAGAAGACGTGGGAAAAACTTGAATGTCACCTGCGACGAGAGAGAAGGGAACGTACTTCCCATGGCTAGCGTTCGGTTCTTCAGAGCGGATCTGCACTCCATCGTACAGGAAACGGCAGAGATACGCGATACGGCCGCGTCCCTCCAGGCGCCCCTGAGTGTCGCTACAGAACCGAATCAACTGCCGATCTCATTTGGAGCACCAAACGAGACTTTCCCCATAACGTTCGGGGATTTTGATGAAGGGGAGATTGAAAGCTTGTCCTCTGAGTTACTGACCTTTGGGGACTTCTCGCCGGGCGAAGTGGATGACCTGACAGACAGCGACTGGTCCACGTGTTCAGACACGGACGACGAATTATGACTAGATAGGGCAGGTGGGTAGGTCGACCTCGACTAGTGCACTCAACAAAAATGTAATATTAAACACAATTAAATAAATGTTAAAATTTATTGCCTAATATTATTTTGTCATTGCTTGTCATTTATTAATTTGGATGATGTCATTTGTTTTTAAAATTGAACTGGCTTTACGAGTAGAATTCGAGCTCCAATTCGCCCTATAGTGAGTCGTATTACAATTCACTGGCCGTCGTTTTACAACGTCGTGACTGGGAAAACCCTGGCGTTACCCAACTTAATCGCCTTGCAGCACATCCCCCTTTCGCCAGCTGGCGTAATAGCGAAGAGGCCCGCACCGATCGCCCTTCCCAACAGTTGCGCAGCCTGAATGGCGAATGGCGCGACGCGCCCTGTAGCGGCGCATTAAGCGCGGCGGGTGTGGTGGTTACGCGCAGCGTGACCGCTACACTTGCCAGCGCCCTAGCGCCCGCTCCTTTCGCTTTCTTCCCTTCCTTTCTCGCCACGTTCGCCGGCTTTCCCCGTCAAGCTCTAAATCGGGGGCTCCCTTTAGGGTTCCGATTTAGTGCTTTACGGCACCTCGACCCCAAAAAACTTGATTAGGGTGATGGTTCACGTAGTGGGCCATCGCCCTGATAGACGGTTTTTCGCCCTTTGACGTTGGAGTCCACGTTCTTTAATAGTGGACTCTTGTTCCAAACTGGAACAACACTCAACCCTATCTCGGTCTATTCTTTTGATTTATAAGGGATTTTGCCGATTTCGGCCTATTGGTTAAAAAATGAGCTGATTTAACAAAAATTTAACGCGAATTTTAACAAAATATTAACGTTTACAATTTCCCAGGTGGCACTTTTCGGGGAAATGTGCGCGGAACCCCTATTTGTTTATTTTTCTAAATACATTCAAATATGTATCCGCTCATGAGACAATAACCCTGATAAATGCTTCAATAATATTGAAAAAGGAAGAGTATGAGTATTCAACATTTCCGTGTCGCCCTTATTCCCTTTTTTGCGGCATTTTGCCTTCCTGTTTTTGCTCACCCAGAAACGCTGGTGAAAGTAAAAGATGCTGAAGATCAGTTGGGTGCACGAGTGGGTTACATCGAACTGGATCTCAACAGCGGTAAGATCCTTGAGAGTTTTCGCCCCGAAGAACGTTTTCCAATGATGAGCACTTTTAAAGTTCTGCTATGTGGCGCGGTATTATCCCGTATTGACGCCGGGCAAGAGCAACTCGGTCGCCGCATACACTATTCTCAGAATGACTTGGTTGAGTACTCACCAGTCACAGAAAAGCATCTTACGGATGGCATGACAGTAAGAGAATTATGCAGTGCTGCCATAACCATGAGTGATAACACTGCGGCCAACTTACTTCTGACAACGATCGGAGGACCGAAGGAGCTAACCGCTTTTTTGCACAACATGGGGGATCATGTAACTCGCCTTGATCGTTGGGAACCGGAGCTGAATGAAGCCATACCAAACGACGAGCGTGACACCACGATGCCTGTAGCAATGGCAACAACGTTGCGCAAACTATTAACTGGCGAACTACTTACTCTAGCTTCCCGGCAACAATTAATAGACTGGATGGAGGCGGATAAAGTTGCAGGACCACTTCTGCGCTCGGCCCTTCCGGCTGGCTGGTTTATTGCTGATAAATCTGGAGCCGGTGAGCGTGGGTCTCGCGGTATCATTGCAGCACTGGGGCCAGATGGTAAGCCCTCCCGTATCGTAGTTATCTACACGACGGGGAGTCAGGCAACTATGGATGAACGAAATAGACAGATCGCTGAGATAGGTGCCTCACTGATTAAGCATTGGTAACTGTCAGACCAAGTTTACTCATATATACTTTAGATTGATTTAAAACTTCATTTTTAATTTAAAAGGATCTAGGTGAAGATCCTTTTTGATAATCTCATGACCAAAATCCCTTAACGTGAGTTTTCGTTCCACTGAGCGTCAGACCCCGTAGAAAAGATCAAAGGATCTTCTTGAGATCCTTTTTTTCTGCGCGTAATCTGCTGCTTGCAAACAAAAAAACCACCGCTACCAGCGGTGGTTTGTTTGCCGGATCAAGAGCTACCAACTCTTTTTCCGAAGGTAACTGGCTTCAGCAGAGCGCAGATACCAAATACTGTCCTTCTAGTGTAGCCGTAGTTAGGCCACCACTTCAAGAACTCTGTAGCACCGCCTACATACCTCGCTCTGCTAATCCTGTTACCAGTGGCTGCTGCCAGTGGCGATAAGTCGTGTCTTACCGGGTTGGACTCAAGACGATAGTTACCGGATAAGGCGCAGCGGTCGGGCTGAACGGGGGGTTCGTGCACACAGCCCAGCTTGGAGCGAACGACCTACACCGAACTGAGATACCTACAGCGTGAGCTATGAGAAAGCGCCACGCTTCCCGAAGGGAGAAAGGCGGACAGGTATCCGGTAAGCGGCAGGGTCGGAACAGGAGAGCGCACGAGGGAGCTTCCAGGGGGAAACGCCTGGTATCTTTATAGTCCTGTCGGGTTTCGCCACCTCTGACTTGAGCGTCGATTTTTGTGATGCTCGTCAGGGGGGCGGAGCCTATGGAAAAACGCCAGCAACGCGGCCTTTTTACGGTTCCTGGCCTTTTGCTGGCCTTTTGCTCACATGTTCTTTCCTGCGTTATCCCCTGATTCTGTGGATAACCGTATTACCGCCTTTGAGTGAGCTGATACCGCTCGCCGCAGCCGAACGACCGAGCGCAGCGAGTCAGTGAGCGAGGAAGCGGAAGAGCGCCCAATACGCAAACCGCCTCTCCCCGCGCGTTGGCCGATTCATTAATGCAGCTGGCACGACAGGTTTCCCGACTGGAAAGCGGGCAGTGAGCGCAACGCAATTAATGTGAGTTAGCTCACTCATTAGGCACCCCAGGCTTTACACTTTATGCTTCCGGCTCGTATGTTGTGTGGAATTGTGAGCGGATAACAATTTCACACAGGAAACAGCTATGACCATGATTACGCCAAGCTCGGAATTAACCCTCACTAAAGGGAACAAAAGCTGGGTAC

>pIE1-hr5-HA-CHIKnsP3 arg PA

CGGGCCCCCCCTCGAGGGGGATCCGGCGCGTAAAACACAATCAAGTATGAGTCATAAGCTGATGTCATGTTTTGCACACGGCTCATAACCGAACTGGCTTTACGAGTAGAATTCTACTTGTAACGCACGATCAGTGGATGATGGTCATTTGTTTTTCAAATCGAGATGATGTCATGTTTTGCACACGGGCTCATAAACTGCTTTACGAGTAGAATTCTACGTGTAACGCACGATCGAGATGAGTCATTTGTTTTGCAATATGATATCATACAATATGACTCATTTGTTTTTCAAAACCGAACTTGATTTACGGGTAGAATTCTACTCGTAAAGCACAATCAAAAAGATGATGTCATTTGTTTTTCAAAACTGAACTCTCGGCTTTACGAGTAGAATTCTACGTGTAAAACACAATCAAGAAATGATGTCATTTGTTATAAAAATAAAAGCTGATGTCATGTTTTGCACATGGCTCATAACTAAACTCGCTTTACGGGTAGAATTCTACGCGCCGGATCCACTAGCTAGTTCTAGAGTCGCGATGTCTTTGTGATGCGCCGACATTTTTGTAGGTTATTGATAAAATGAACGGATACAGTTGCCCGACATTATCATTAAATCCTTGGCGTAGAATTTGTCGGGTCCATTGTCCGTGTGCGCTAGCATGCCCGCTAACGGACCTCGTACTTTTGGCTTCAAAGGTTTTGCGCACAGACAAAATGTGCCACACTTGCAGCTCTGCATGTGTGCGCGTTACCACAAATCCCAACGGCGCAGTGTACTTGTTGTATGCAAATAAATCTCGATAAAGGCGCGGCGCGCGAATGCAGCTGATCACGTACGCTCCTCGTGTTCCGTTCAAGGACGGTGTTATCGACCTCAGATTAATGTTTATCGGCCGACTGTTTTCGTATCCGCTCACCAAACGCGTTTTTGCATTAACATTGTATGTCGGCGGATGTTCTATATCTAATTTGAATAAATAAACGATAACCGCGTTGGTTTTAGAGGGCATAATAAAAGAAATATTGTTATCGTGTTCGCCATTAGGGCAGTATAAATTGACGTTCATGTTGGATATTGTTTCAGTTGCAAGTTGACACTGGCGGCGACAAGAGATCTGCCGGGCTGCAGGAATTCGATATCAAGCTTATCGATGTACCCATACGACGTCCCAGACTACGCACCGTCGTACCGGGTTAAACGCATGGACATCGCAAAGAACGATGAAGAGTGTGTAGTCAACGCCGCCAACCCTCGTGGGCTACCAGGCGATGGCGTCTGTAAAGCAGTATACAAAAAATGGCCGGAGTCCTTCAAGAACAGTGCAACACCAGTGGGAACCGCAAAGACAGTCATGTGCGGTACATACCCGGTAATCCATGCAGTAGGACCTAATTTCTCAAATTACTCTGAGTCCGAAGGAGACCGGGAATTGGCAGCTGCTTACCGAGAAGTCGCTAAGGAGGTGACTAGACTAGGAGTAAACAGCGTAGCTATACCGCTCCTTTCCACCGGTGTGTACTCTGGAGGGAAAGACAGGCTGACTCAGTCACTAAACCACCTTTTTACAGCATTAGACTCAACTGATGCAGATGTGGTTATCTACTGCCGCGACAAGGAGTGGGAGAAGAAAATAGCTGAGGCCATACAAATGAGGACCCAAGTGGAATTACTAGACGAACACATCTCTGTAGACTGCGATATCATCCGAGTGCACCCTGACAGCAGTTTGGCAGGTAGAAAAGGGTACAGCACTACAGAAGGTTCACTGTACTCCTACTTGGAAGGGACACGGTTCCATCAGACGGCAGTGGACATGGCAGAAGTATACACCATGTGGCCAAAGCAGACGGAGGCTAATGAACAAGTTTGCTTGTACGCATTGGGGGAAAGTATAGAATCAATCAGGCAAAAGTGCCCAGTGGATGACGCAGATGCATCGTCGCCCCCAAAAACCGTCCCGTGCCTCTGCCGTTATGCCATGACACCCGAACGAGTCACCAGGCTTCGTATGAACCATGTCACAAGCATAATAGTATGCTCATCATTCCCCCTTCCAAAGTATAAAATAGAAGGAGTGCAGAAAGTCAAGTGTTCTAAAGTGATGCTGTTCGACCATAACGTGCCATCACGCGTTAGTCCAAGGGAATATAAATCGCCTCAGGAGACCGCACAAGAAGTAAGTTCGACCACGTCACTGACGCACAGCCAATTCGACCTTAGCGTTGACGGTGAGGAACTGCCCGCTCCGTCTGACTTGGAAGCTGACGCTCCGATTCCGGAACCAACACCAGACGACAGAGCGGTACTTACTTTGCCTCCCACGATTGATAATTTTTCGGCTGTGTCAGACTGGGTAATGAATACCGCGCCAGTCGCACCACCCAGAAGAAGACGTGGGAAAAACTTGAATGTCACCTGCGACGAGAGAGAAGGGAACGTACTTCCCATGGCTAGCGTTCGGTTCTTCAGAGCGGATCTGCACTCCATCGTACAGGAAACGGCAGAGATACGCGATACGGCCGCGTCCCTCCAGGCGCCCCTGAGTGTCGCTACAGAACCGAATCAACTGCCGATCTCATTTGGAGCACCAAACGAGACTTTCCCCATAACGTTCGGGGATTTTGATGAAGGGGAGATTGAAAGCTTGTCCTCTGAGTTACTGACCTTTGGGGACTTCTCGCCGGGCGAAGTGGATGACCTGACAGACAGCGACTGGTCCACGTGTTCAGACACGGACGACGAATTACGACTAGATAGGGCAGGTGGGTAGGTCGACCTCGACTAGTGCACTCAACAAAAATGTAATATTAAACACAATTAAATAAATGTTAAAATTTATTGCCTAATATTATTTTGTCATTGCTTGTCATTTATTAATTTGGATGATGTCATTTGTTTTTAAAATTGAACTGGCTTTACGAGTAGAATTCGAGCTCCAATTCGCCCTATAGTGAGTCGTATTACAATTCACTGGCCGTCGTTTTACAACGTCGTGACTGGGAAAACCCTGGCGTTACCCAACTTAATCGCCTTGCAGCACATCCCCCTTTCGCCAGCTGGCGTAATAGCGAAGAGGCCCGCACCGATCGCCCTTCCCAACAGTTGCGCAGCCTGAATGGCGAATGGCGCGACGCGCCCTGTAGCGGCGCATTAAGCGCGGCGGGTGTGGTGGTTACGCGCAGCGTGACCGCTACACTTGCCAGCGCCCTAGCGCCCGCTCCTTTCGCTTTCTTCCCTTCCTTTCTCGCCACGTTCGCCGGCTTTCCCCGTCAAGCTCTAAATCGGGGGCTCCCTTTAGGGTTCCGATTTAGTGCTTTACGGCACCTCGACCCCAAAAAACTTGATTAGGGTGATGGTTCACGTAGTGGGCCATCGCCCTGATAGACGGTTTTTCGCCCTTTGACGTTGGAGTCCACGTTCTTTAATAGTGGACTCTTGTTCCAAACTGGAACAACACTCAACCCTATCTCGGTCTATTCTTTTGATTTATAAGGGATTTTGCCGATTTCGGCCTATTGGTTAAAAAATGAGCTGATTTAACAAAAATTTAACGCGAATTTTAACAAAATATTAACGTTTACAATTTCCCAGGTGGCACTTTTCGGGGAAATGTGCGCGGAACCCCTATTTGTTTATTTTTCTAAATACATTCAAATATGTATCCGCTCATGAGACAATAACCCTGATAAATGCTTCAATAATATTGAAAAAGGAAGAGTATGAGTATTCAACATTTCCGTGTCGCCCTTATTCCCTTTTTTGCGGCATTTTGCCTTCCTGTTTTTGCTCACCCAGAAACGCTGGTGAAAGTAAAAGATGCTGAAGATCAGTTGGGTGCACGAGTGGGTTACATCGAACTGGATCTCAACAGCGGTAAGATCCTTGAGAGTTTTCGCCCCGAAGAACGTTTTCCAATGATGAGCACTTTTAAAGTTCTGCTATGTGGCGCGGTATTATCCCGTATTGACGCCGGGCAAGAGCAACTCGGTCGCCGCATACACTATTCTCAGAATGACTTGGTTGAGTACTCACCAGTCACAGAAAAGCATCTTACGGATGGCATGACAGTAAGAGAATTATGCAGTGCTGCCATAACCATGAGTGATAACACTGCGGCCAACTTACTTCTGACAACGATCGGAGGACCGAAGGAGCTAACCGCTTTTTTGCACAACATGGGGGATCATGTAACTCGCCTTGATCGTTGGGAACCGGAGCTGAATGAAGCCATACCAAACGACGAGCGTGACACCACGATGCCTGTAGCAATGGCAACAACGTTGCGCAAACTATTAACTGGCGAACTACTTACTCTAGCTTCCCGGCAACAATTAATAGACTGGATGGAGGCGGATAAAGTTGCAGGACCACTTCTGCGCTCGGCCCTTCCGGCTGGCTGGTTTATTGCTGATAAATCTGGAGCCGGTGAGCGTGGGTCTCGCGGTATCATTGCAGCACTGGGGCCAGATGGTAAGCCCTCCCGTATCGTAGTTATCTACACGACGGGGAGTCAGGCAACTATGGATGAACGAAATAGACAGATCGCTGAGATAGGTGCCTCACTGATTAAGCATTGGTAACTGTCAGACCAAGTTTACTCATATATACTTTAGATTGATTTAAAACTTCATTTTTAATTTAAAAGGATCTAGGTGAAGATCCTTTTTGATAATCTCATGACCAAAATCCCTTAACGTGAGTTTTCGTTCCACTGAGCGTCAGACCCCGTAGAAAAGATCAAAGGATCTTCTTGAGATCCTTTTTTTCTGCGCGTAATCTGCTGCTTGCAAACAAAAAAACCACCGCTACCAGCGGTGGTTTGTTTGCCGGATCAAGAGCTACCAACTCTTTTTCCGAAGGTAACTGGCTTCAGCAGAGCGCAGATACCAAATACTGTCCTTCTAGTGTAGCCGTAGTTAGGCCACCACTTCAAGAACTCTGTAGCACCGCCTACATACCTCGCTCTGCTAATCCTGTTACCAGTGGCTGCTGCCAGTGGCGATAAGTCGTGTCTTACCGGGTTGGACTCAAGACGATAGTTACCGGATAAGGCGCAGCGGTCGGGCTGAACGGGGGGTTCGTGCACACAGCCCAGCTTGGAGCGAACGACCTACACCGAACTGAGATACCTACAGCGTGAGCTATGAGAAAGCGCCACGCTTCCCGAAGGGAGAAAGGCGGACAGGTATCCGGTAAGCGGCAGGGTCGGAACAGGAGAGCGCACGAGGGAGCTTCCAGGGGGAAACGCCTGGTATCTTTATAGTCCTGTCGGGTTTCGCCACCTCTGACTTGAGCGTCGATTTTTGTGATGCTCGTCAGGGGGGCGGAGCCTATGGAAAAACGCCAGCAACGCGGCCTTTTTACGGTTCCTGGCCTTTTGCTGGCCTTTTGCTCACATGTTCTTTCCTGCGTTATCCCCTGATTCTGTGGATAACCGTATTACCGCCTTTGAGTGAGCTGATACCGCTCGCCGCAGCCGAACGACCGAGCGCAGCGAGTCAGTGAGCGAGGAAGCGGAAGAGCGCCCAATACGCAAACCGCCTCTCCCCGCGCGTTGGCCGATTCATTAATGCAGCTGGCACGACAGGTTTCCCGACTGGAAAGCGGGCAGTGAGCGCAACGCAATTAATGTGAGTTAGCTCACTCATTAGGCACCCCAGGCTTTACACTTTATGCTTCCGGCTCGTATGTTGTGTGGAATTGTGAGCGGATAACAATTTCACACAGGAAACAGCTATGACCATGATTACGCCAAGCTCGGAATTAACCCTCACTAAAGGGAACAAAAGCTGGGTAC

>pIE1-hr5-HA-ONNnsP3 opal PA

CGGGCCCCCCCTCGAGGGGGATCCGGCGCGTAAAACACAATCAAGTATGAGTCATAAGCTGATGTCATGTTTTGCACACGGCTCATAACCGAACTGGCTTTACGAGTAGAATTCTACTTGTAACGCACGATCAGTGGATGATGGTCATTTGTTTTTCAAATCGAGATGATGTCATGTTTTGCACACGGGCTCATAAACTGCTTTACGAGTAGAATTCTACGTGTAACGCACGATCGAGATGAGTCATTTGTTTTGCAATATGATATCATACAATATGACTCATTTGTTTTTCAAAACCGAACTTGATTTACGGGTAGAATTCTACTCGTAAAGCACAATCAAAAAGATGATGTCATTTGTTTTTCAAAACTGAACTCTCGGCTTTACGAGTAGAATTCTACGTGTAAAACACAATCAAGAAATGATGTCATTTGTTATAAAAATAAAAGCTGATGTCATGTTTTGCACATGGCTCATAACTAAACTCGCTTTACGGGTAGAATTCTACGCGCCGGATCCACTAGCTAGTTCTAGAGTCGCGATGTCTTTGTGATGCGCCGACATTTTTGTAGGTTATTGATAAAATGAACGGATACAGTTGCCCGACATTATCATTAAATCCTTGGCGTAGAATTTGTCGGGTCCATTGTCCGTGTGCGCTAGCATGCCCGCTAACGGACCTCGTACTTTTGGCTTCAAAGGTTTTGCGCACAGACAAAATGTGCCACACTTGCAGCTCTGCATGTGTGCGCGTTACCACAAATCCCAACGGCGCAGTGTACTTGTTGTATGCAAATAAATCTCGATAAAGGCGCGGCGCGCGAATGCAGCTGATCACGTACGCTCCTCGTGTTCCGTTCAAGGACGGTGTTATCGACCTCAGATTAATGTTTATCGGCCGACTGTTTTCGTATCCGCTCACCAAACGCGTTTTTGCATTAACATTGTATGTCGGCGGATGTTCTATATCTAATTTGAATAAATAAACGATAACCGCGTTGGTTTTAGAGGGCATAATAAAAGAAATATTGTTATCGTGTTCGCCATTAGGGCAGTATAAATTGACGTTCATGTTGGATATTGTTTCAGTTGCAAGTTGACACTGGCGGCGACAAGAGATCTGCCGGGCTGCAGGAATTCGATATCAAGCTTATCGATGTACCCATACGACGTCCCAGACTACGCTCCGTCATACCGTGTGAAACGGATGGACATCGCGAAAAACACTGAGGAATGCGTGGTAAACGCCGCCAATCCACGCGGAGTACCAGGCGATGGAGTATGTAAAGCCGTGTATAGAAAATGGCCAGAATCATTCAGAAACAGTGCAACGCCAGTGGGTACTGCAAAGACAATCATGTGCGGTCAATACCCCGTCATCCACGCGGTAGGCCCAAACTTCTCAAACTATTCTGAGGCTGAAGGGGATAGGGAATTGGCTTCAGTGTATAGAGAAGTGGCGAAAGAAGTGTCTAGGCTAGGAGTGAGCAGTGTAGCCATCCCTTTGCTTTCAACCGGTGTGTACTCAGGAGGCAAAGATAGACTGCTGCAATCACTAAACCATCTTTTCACAGCGATGGATTCGACAGATGCAGATGTTGTCATCTATTGCAGGGATAAGGAATGGGAGAAGAAGATCACTGAAGCCATATCATTAAGATCCCAGGTAGAACTACTAGATGATCACATCTCAGTGGATTGCGACATTGTACGCGTTCATCCAGACAGCAGCTTGGCAGGCCGAAAGGGGTACAGCACAGTAGAGGGAGCACTCTACTCGTACCTAGAGGGAACAAGATTCCACCAAACTGCTGTAGATATGGCAGAAATATATACCATGTGGCCAAAACAAACTGAAGCCAATGAACAGGTCTGCCTATATGCTCTGGGGGAGAGCATAGAGTCAGTCAGGCAAAAATGTCCTGTAGACGACGCCGACGCCTCATTCCCTCCGAAAACAGTCCCGTGCCTATGCCGTTATGCCATGACGCCTGAACGAGTTGCACGCCTACGCATGAATCATACTACTAGCATCATAGTGTGCTCGTCTTTTCCACTGCCGAAGTACAAAATCGAGGGCGTGCAAAAAGTAAAATGTTCAAAAGCACTCTTGTTTGATCACAACGTACCGTCTCGAGTGAGCCCGAGAACGTACAGGCCTGCGGACGAAATCATACAGACACCTCAAATACCAACTGAAGCGTGCCAGGACGCACAATTCGTGCAGTCAATAACTGATGAAGCAGTGCCAGTTCCGTCAGACTTAGAGGCTTGTGACGCAACTATGGACTGGCCCTCTATCGACATCGTACCAACAAGACAAAGAAGCGACTCATTTGACAGCGAGTATAGTTCCAGAAGTAACATACAGCTGGTGACAGCGGACGTGCATGCACCAATGTACGCAAATTCGCTGGCGTCCAGCGGAGGTTCAGTGCTGTCGCTGTCCAGTGAACAAGCTCAGAACGGCATAATGATACTACCTGATTCAGAAGACACAGATAGTATAAGCAGAGTAAGCACACCGATCGCCCCACCCAGGAGACGTTTGGGAAGGACTATAAATGTGACTTGTGACGAGCGGGAAGGGAAAATACTCCCTATGGCCAGCGACAGGCTCTTCACTGCTAAGCCATACACTGTCGCACTGGGCGTATCAACAGCAGACATAACTGCGTACCCCATCCAGGCACCGCTAGGATCGACACAACCGCCTGCCCTCGAACAGATCACTTTCGGAGATTTCGCCGAAGGTGAAATAGACAACCTCCTGACAGGGGCATTGACATTTGGAGACTTCGAGCCAGGTGAAGTGGAAGAGCTGACGGATAGCGAGTGGTCAACATGCTCGGACACAGATGAAGAGTTATGAGTCGACCTCGACTAGTGCACTCAACAAAAATGTAATATTAAACACAATTAAATAAATGTTAAAATTTATTGCCTAATATTATTTTGTCATTGCTTGTCATTTATTAATTTGGATGATGTCATTTGTTTTTAAAATTGAACTGGCTTTACGAGTAGAATTCGAGCTCCAATTCGCCCTATAGTGAGTCGTATTACAATTCACTGGCCGTCGTTTTACAACGTCGTGACTGGGAAAACCCTGGCGTTACCCAACTTAATCGCCTTGCAGCACATCCCCCTTTCGCCAGCTGGCGTAATAGCGAAGAGGCCCGCACCGATCGCCCTTCCCAACAGTTGCGCAGCCTGAATGGCGAATGGCGCGACGCGCCCTGTAGCGGCGCATTAAGCGCGGCGGGTGTGGTGGTTACGCGCAGCGTGACCGCTACACTTGCCAGCGCCCTAGCGCCCGCTCCTTTCGCTTTCTTCCCTTCCTTTCTCGCCACGTTCGCCGGCTTTCCCCGTCAAGCTCTAAATCGGGGGCTCCCTTTAGGGTTCCGATTTAGTGCTTTACGGCACCTCGACCCCAAAAAACTTGATTAGGGTGATGGTTCACGTAGTGGGCCATCGCCCTGATAGACGGTTTTTCGCCCTTTGACGTTGGAGTCCACGTTCTTTAATAGTGGACTCTTGTTCCAAACTGGAACAACACTCAACCCTATCTCGGTCTATTCTTTTGATTTATAAGGGATTTTGCCGATTTCGGCCTATTGGTTAAAAAATGAGCTGATTTAACAAAAATTTAACGCGAATTTTAACAAAATATTAACGTTTACAATTTCCCAGGTGGCACTTTTCGGGGAAATGTGCGCGGAACCCCTATTTGTTTATTTTTCTAAATACATTCAAATATGTATCCGCTCATGAGACAATAACCCTGATAAATGCTTCAATAATATTGAAAAAGGAAGAGTATGAGTATTCAACATTTCCGTGTCGCCCTTATTCCCTTTTTTGCGGCATTTTGCCTTCCTGTTTTTGCTCACCCAGAAACGCTGGTGAAAGTAAAAGATGCTGAAGATCAGTTGGGTGCACGAGTGGGTTACATCGAACTGGATCTCAACAGCGGTAAGATCCTTGAGAGTTTTCGCCCCGAAGAACGTTTTCCAATGATGAGCACTTTTAAAGTTCTGCTATGTGGCGCGGTATTATCCCGTATTGACGCCGGGCAAGAGCAACTCGGTCGCCGCATACACTATTCTCAGAATGACTTGGTTGAGTACTCACCAGTCACAGAAAAGCATCTTACGGATGGCATGACAGTAAGAGAATTATGCAGTGCTGCCATAACCATGAGTGATAACACTGCGGCCAACTTACTTCTGACAACGATCGGAGGACCGAAGGAGCTAACCGCTTTTTTGCACAACATGGGGGATCATGTAACTCGCCTTGATCGTTGGGAACCGGAGCTGAATGAAGCCATACCAAACGACGAGCGTGACACCACGATGCCTGTAGCAATGGCAACAACGTTGCGCAAACTATTAACTGGCGAACTACTTACTCTAGCTTCCCGGCAACAATTAATAGACTGGATGGAGGCGGATAAAGTTGCAGGACCACTTCTGCGCTCGGCCCTTCCGGCTGGCTGGTTTATTGCTGATAAATCTGGAGCCGGTGAGCGTGGGTCTCGCGGTATCATTGCAGCACTGGGGCCAGATGGTAAGCCCTCCCGTATCGTAGTTATCTACACGACGGGGAGTCAGGCAACTATGGATGAACGAAATAGACAGATCGCTGAGATAGGTGCCTCACTGATTAAGCATTGGTAACTGTCAGACCAAGTTTACTCATATATACTTTAGATTGATTTAAAACTTCATTTTTAATTTAAAAGGATCTAGGTGAAGATCCTTTTTGATAATCTCATGACCAAAATCCCTTAACGTGAGTTTTCGTTCCACTGAGCGTCAGACCCCGTAGAAAAGATCAAAGGATCTTCTTGAGATCCTTTTTTTCTGCGCGTAATCTGCTGCTTGCAAACAAAAAAACCACCGCTACCAGCGGTGGTTTGTTTGCCGGATCAAGAGCTACCAACTCTTTTTCCGAAGGTAACTGGCTTCAGCAGAGCGCAGATACCAAATACTGTCCTTCTAGTGTAGCCGTAGTTAGGCCACCACTTCAAGAACTCTGTAGCACCGCCTACATACCTCGCTCTGCTAATCCTGTTACCAGTGGCTGCTGCCAGTGGCGATAAGTCGTGTCTTACCGGGTTGGACTCAAGACGATAGTTACCGGATAAGGCGCAGCGGTCGGGCTGAACGGGGGGTTCGTGCACACAGCCCAGCTTGGAGCGAACGACCTACACCGAACTGAGATACCTACAGCGTGAGCTATGAGAAAGCGCCACGCTTCCCGAAGGGAGAAAGGCGGACAGGTATCCGGTAAGCGGCAGGGTCGGAACAGGAGAGCGCACGAGGGAGCTTCCAGGGGGAAACGCCTGGTATCTTTATAGTCCTGTCGGGTTTCGCCACCTCTGACTTGAGCGTCGATTTTTGTGATGCTCGTCAGGGGGGCGGAGCCTATGGAAAAACGCCAGCAACGCGGCCTTTTTACGGTTCCTGGCCTTTTGCTGGCCTTTTGCTCACATGTTCTTTCCTGCGTTATCCCCTGATTCTGTGGATAACCGTATTACCGCCTTTGAGTGAGCTGATACCGCTCGCCGCAGCCGAACGACCGAGCGCAGCGAGTCAGTGAGCGAGGAAGCGGAAGAGCGCCCAATACGCAAACCGCCTCTCCCCGCGCGTTGGCCGATTCATTAATGCAGCTGGCACGACAGGTTTCCCGACTGGAAAGCGGGCAGTGAGCGCAACGCAATTAATGTGAGTTAGCTCACTCATTAGGCACCCCAGGCTTTACACTTTATGCTTCCGGCTCGTATGTTGTGTGGAATTGTGAGCGGATAACAATTTCACACAGGAAACAGCTATGACCATGATTACGCCAAGCTCGGAATTAACCCTCACTAAAGGGAACAAAAGCTGGGTAC

>pIE1-hr5-HA-ONNnsP3 arg PA

CGGGCCCCCCCTCGAGGGGGATCCGGCGCGTAAAACACAATCAAGTATGAGTCATAAGCTGATGTCATGTTTTGCACACGGCTCATAACCGAACTGGCTTTACGAGTAGAATTCTACTTGTAACGCACGATCAGTGGATGATGGTCATTTGTTTTTCAAATCGAGATGATGTCATGTTTTGCACACGGGCTCATAAACTGCTTTACGAGTAGAATTCTACGTGTAACGCACGATCGAGATGAGTCATTTGTTTTGCAATATGATATCATACAATATGACTCATTTGTTTTTCAAAACCGAACTTGATTTACGGGTAGAATTCTACTCGTAAAGCACAATCAAAAAGATGATGTCATTTGTTTTTCAAAACTGAACTCTCGGCTTTACGAGTAGAATTCTACGTGTAAAACACAATCAAGAAATGATGTCATTTGTTATAAAAATAAAAGCTGATGTCATGTTTTGCACATGGCTCATAACTAAACTCGCTTTACGGGTAGAATTCTACGCGCCGGATCCACTAGCTAGTTCTAGAGTCGCGATGTCTTTGTGATGCGCCGACATTTTTGTAGGTTATTGATAAAATGAACGGATACAGTTGCCCGACATTATCATTAAATCCTTGGCGTAGAATTTGTCGGGTCCATTGTCCGTGTGCGCTAGCATGCCCGCTAACGGACCTCGTACTTTTGGCTTCAAAGGTTTTGCGCACAGACAAAATGTGCCACACTTGCAGCTCTGCATGTGTGCGCGTTACCACAAATCCCAACGGCGCAGTGTACTTGTTGTATGCAAATAAATCTCGATAAAGGCGCGGCGCGCGAATGCAGCTGATCACGTACGCTCCTCGTGTTCCGTTCAAGGACGGTGTTATCGACCTCAGATTAATGTTTATCGGCCGACTGTTTTCGTATCCGCTCACCAAACGCGTTTTTGCATTAACATTGTATGTCGGCGGATGTTCTATATCTAATTTGAATAAATAAACGATAACCGCGTTGGTTTTAGAGGGCATAATAAAAGAAATATTGTTATCGTGTTCGCCATTAGGGCAGTATAAATTGACGTTCATGTTGGATATTGTTTCAGTTGCAAGTTGACACTGGCGGCGACAAGAGATCTGCCGGGCTGCAGGAATTCGATATCAAGCTTATCGATGTACCCATACGACGTCCCAGACTACGCTCCGTCATACCGTGTGAAACGGATGGACATCGCGAAAAACACTGAGGAATGCGTGGTAAACGCCGCCAATCCACGCGGAGTACCAGGCGATGGAGTATGTAAAGCCGTGTATAGAAAATGGCCAGAATCATTCAGAAACAGTGCAACGCCAGTGGGTACTGCAAAGACAATCATGTGCGGTCAATACCCCGTCATCCACGCGGTAGGCCCAAACTTCTCAAACTATTCTGAGGCTGAAGGGGATAGGGAATTGGCTTCAGTGTATAGAGAAGTGGCGAAAGAAGTGTCTAGGCTAGGAGTGAGCAGTGTAGCCATCCCTTTGCTTTCAACCGGTGTGTACTCAGGAGGCAAAGATAGACTGCTGCAATCACTAAACCATCTTTTCACAGCGATGGATTCGACAGATGCAGATGTTGTCATCTATTGCAGGGATAAGGAATGGGAGAAGAAGATCACTGAAGCCATATCATTAAGATCCCAGGTAGAACTACTAGATGATCACATCTCAGTGGATTGCGACATTGTACGCGTTCATCCAGACAGCAGCTTGGCAGGCCGAAAGGGGTACAGCACAGTAGAGGGAGCACTCTACTCGTACCTAGAGGGAACAAGATTCCACCAAACTGCTGTAGATATGGCAGAAATATATACCATGTGGCCAAAACAAACTGAAGCCAATGAACAGGTCTGCCTATATGCTCTGGGGGAGAGCATAGAGTCAGTCAGGCAAAAATGTCCTGTAGACGACGCCGACGCCTCATTCCCTCCGAAAACAGTCCCGTGCCTATGCCGTTATGCCATGACGCCTGAACGAGTTGCACGCCTACGCATGAATCATACTACTAGCATCATAGTGTGCTCGTCTTTTCCACTGCCGAAGTACAAAATCGAGGGCGTGCAAAAAGTAAAATGTTCAAAAGCACTCTTGTTTGATCACAACGTACCGTCTCGAGTGAGCCCGAGAACGTACAGGCCTGCGGACGAAATCATACAGACACCTCAAATACCAACTGAAGCGTGCCAGGACGCACAATTCGTGCAGTCAATAACTGATGAAGCAGTGCCAGTTCCGTCAGACTTAGAGGCTTGTGACGCAACTATGGACTGGCCCTCTATCGACATCGTACCAACAAGACAAAGAAGCGACTCATTTGACAGCGAGTATAGTTCCAGAAGTAACATACAGCTGGTGACAGCGGACGTGCATGCACCAATGTACGCAAATTCGCTGGCGTCCAGCGGAGGTTCAGTGCTGTCGCTGTCCAGTGAACAAGCTCAGAACGGCATAATGATACTACCTGATTCAGAAGACACAGATAGTATAAGCAGAGTAAGCACACCGATCGCCCCACCCAGGAGACGTTTGGGAAGGACTATAAATGTGACTTGTGACGAGCGGGAAGGGAAAATACTCCCTATGGCCAGCGACAGGCTCTTCACTGCTAAGCCATACACTGTCGCACTGGGCGTATCAACAGCAGACATAACTGCGTACCCCATCCAGGCACCGCTAGGATCGACACAACCGCCTGCCCTCGAACAGATCACTTTCGGAGATTTCGCCGAAGGTGAAATAGACAACCTCCTGACAGGGGCATTGACATTTGGAGACTTCGAGCCAGGTGAAGTGGAAGAGCTGACGGATAGCGAGTGGTCAACATGCTCGGACACAGATGAAGAGTTACGACTAGACAGAGCAGGGGGTTAGATATTCTCCTCTGACACTGGTCGTCGACCTCGACTAGTGCACTCAACAAAAATGTAATATTAAACACAATTAAATAAATGTTAAAATTTATTGCCTAATATTATTTTGTCATTGCTTGTCATTTATTAATTTGGATGATGTCATTTGTTTTTAAAATTGAACTGGCTTTACGAGTAGAATTCGAGCTCCAATTCGCCCTATAGTGAGTCGTATTACAATTCACTGGCCGTCGTTTTACAACGTCGTGACTGGGAAAACCCTGGCGTTACCCAACTTAATCGCCTTGCAGCACATCCCCCTTTCGCCAGCTGGCGTAATAGCGAAGAGGCCCGCACCGATCGCCCTTCCCAACAGTTGCGCAGCCTGAATGGCGAATGGCGCGACGCGCCCTGTAGCGGCGCATTAAGCGCGGCGGGTGTGGTGGTTACGCGCAGCGTGACCGCTACACTTGCCAGCGCCCTAGCGCCCGCTCCTTTCGCTTTCTTCCCTTCCTTTCTCGCCACGTTCGCCGGCTTTCCCCGTCAAGCTCTAAATCGGGGGCTCCCTTTAGGGTTCCGATTTAGTGCTTTACGGCACCTCGACCCCAAAAAACTTGATTAGGGTGATGGTTCACGTAGTGGGCCATCGCCCTGATAGACGGTTTTTCGCCCTTTGACGTTGGAGTCCACGTTCTTTAATAGTGGACTCTTGTTCCAAACTGGAACAACACTCAACCCTATCTCGGTCTATTCTTTTGATTTATAAGGGATTTTGCCGATTTCGGCCTATTGGTTAAAAAATGAGCTGATTTAACAAAAATTTAACGCGAATTTTAACAAAATATTAACGTTTACAATTTCCCAGGTGGCACTTTTCGGGGAAATGTGCGCGGAACCCCTATTTGTTTATTTTTCTAAATACATTCAAATATGTATCCGCTCATGAGACAATAACCCTGATAAATGCTTCAATAATATTGAAAAAGGAAGAGTATGAGTATTCAACATTTCCGTGTCGCCCTTATTCCCTTTTTTGCGGCATTTTGCCTTCCTGTTTTTGCTCACCCAGAAACGCTGGTGAAAGTAAAAGATGCTGAAGATCAGTTGGGTGCACGAGTGGGTTACATCGAACTGGATCTCAACAGCGGTAAGATCCTTGAGAGTTTTCGCCCCGAAGAACGTTTTCCAATGATGAGCACTTTTAAAGTTCTGCTATGTGGCGCGGTATTATCCCGTATTGACGCCGGGCAAGAGCAACTCGGTCGCCGCATACACTATTCTCAGAATGACTTGGTTGAGTACTCACCAGTCACAGAAAAGCATCTTACGGATGGCATGACAGTAAGAGAATTATGCAGTGCTGCCATAACCATGAGTGATAACACTGCGGCCAACTTACTTCTGACAACGATCGGAGGACCGAAGGAGCTAACCGCTTTTTTGCACAACATGGGGGATCATGTAACTCGCCTTGATCGTTGGGAACCGGAGCTGAATGAAGCCATACCAAACGACGAGCGTGACACCACGATGCCTGTAGCAATGGCAACAACGTTGCGCAAACTATTAACTGGCGAACTACTTACTCTAGCTTCCCGGCAACAATTAATAGACTGGATGGAGGCGGATAAAGTTGCAGGACCACTTCTGCGCTCGGCCCTTCCGGCTGGCTGGTTTATTGCTGATAAATCTGGAGCCGGTGAGCGTGGGTCTCGCGGTATCATTGCAGCACTGGGGCCAGATGGTAAGCCCTCCCGTATCGTAGTTATCTACACGACGGGGAGTCAGGCAACTATGGATGAACGAAATAGACAGATCGCTGAGATAGGTGCCTCACTGATTAAGCATTGGTAACTGTCAGACCAAGTTTACTCATATATACTTTAGATTGATTTAAAACTTCATTTTTAATTTAAAAGGATCTAGGTGAAGATCCTTTTTGATAATCTCATGACCAAAATCCCTTAACGTGAGTTTTCGTTCCACTGAGCGTCAGACCCCGTAGAAAAGATCAAAGGATCTTCTTGAGATCCTTTTTTTCTGCGCGTAATCTGCTGCTTGCAAACAAAAAAACCACCGCTACCAGCGGTGGTTTGTTTGCCGGATCAAGAGCTACCAACTCTTTTTCCGAAGGTAACTGGCTTCAGCAGAGCGCAGATACCAAATACTGTCCTTCTAGTGTAGCCGTAGTTAGGCCACCACTTCAAGAACTCTGTAGCACCGCCTACATACCTCGCTCTGCTAATCCTGTTACCAGTGGCTGCTGCCAGTGGCGATAAGTCGTGTCTTACCGGGTTGGACTCAAGACGATAGTTACCGGATAAGGCGCAGCGGTCGGGCTGAACGGGGGGTTCGTGCACACAGCCCAGCTTGGAGCGAACGACCTACACCGAACTGAGATACCTACAGCGTGAGCTATGAGAAAGCGCCACGCTTCCCGAAGGGAGAAAGGCGGACAGGTATCCGGTAAGCGGCAGGGTCGGAACAGGAGAGCGCACGAGGGAGCTTCCAGGGGGAAACGCCTGGTATCTTTATAGTCCTGTCGGGTTTCGCCACCTCTGACTTGAGCGTCGATTTTTGTGATGCTCGTCAGGGGGGCGGAGCCTATGGAAAAACGCCAGCAACGCGGCCTTTTTACGGTTCCTGGCCTTTTGCTGGCCTTTTGCTCACATGTTCTTTCCTGCGTTATCCCCTGATTCTGTGGATAACCGTATTACCGCCTTTGAGTGAGCTGATACCGCTCGCCGCAGCCGAACGACCGAGCGCAGCGAGTCAGTGAGCGAGGAAGCGGAAGAGCGCCCAATACGCAAACCGCCTCTCCCCGCGCGTTGGCCGATTCATTAATGCAGCTGGCACGACAGGTTTCCCGACTGGAAAGCGGGCAGTGAGCGCAACGCAATTAATGTGAGTTAGCTCACTCATTAGGCACCCCAGGCTTTACACTTTATGCTTCCGGCTCGTATGTTGTGTGGAATTGTGAGCGGATAACAATTTCACACAGGAAACAGCTATGACCATGATTACGCCAAGCTCGGAATTAACCCTCACTAAAGGGAACAAAAGCTGGGTAC

>pIE1-hr5-GFP-HA-CHIKnsP3 opal PA

CGGGCCCCCCCTCGAGGGGGATCCGGCGCGTAAAACACAATCAAGTATGAGTCATAAGCTGATGTCATGTTTTGCACACGGCTCATAACCGAACTGGCTTTACGAGTAGAATTCTACTTGTAACGCACGATCAGTGGATGATGGTCATTTGTTTTTCAAATCGAGATGATGTCATGTTTTGCACACGGGCTCATAAACTGCTTTACGAGTAGAATTCTACGTGTAACGCACGATCGAGATGAGTCATTTGTTTTGCAATATGATATCATACAATATGACTCATTTGTTTTTCAAAACCGAACTTGATTTACGGGTAGAATTCTACTCGTAAAGCACAATCAAAAAGATGATGTCATTTGTTTTTCAAAACTGAACTCTCGGCTTTACGAGTAGAATTCTACGTGTAAAACACAATCAAGAAATGATGTCATTTGTTATAAAAATAAAAGCTGATGTCATGTTTTGCACATGGCTCATAACTAAACTCGCTTTACGGGTAGAATTCTACGCGCCGGATCCACTAGCTAGTTCTAGAGTCGCGATGTCTTTGTGATGCGCCGACATTTTTGTAGGTTATTGATAAAATGAACGGATACAGTTGCCCGACATTATCATTAAATCCTTGGCGTAGAATTTGTCGGGTCCATTGTCCGTGTGCGCTAGCATGCCCGCTAACGGACCTCGTACTTTTGGCTTCAAAGGTTTTGCGCACAGACAAAATGTGCCACACTTGCAGCTCTGCATGTGTGCGCGTTACCACAAATCCCAACGGCGCAGTGTACTTGTTGTATGCAAATAAATCTCGATAAAGGCGCGGCGCGCGAATGCAGCTGATCACGTACGCTCCTCGTGTTCCGTTCAAGGACGGTGTTATCGACCTCAGATTAATGTTTATCGGCCGACTGTTTTCGTATCCGCTCACCAAACGCGTTTTTGCATTAACATTGTATGTCGGCGGATGTTCTATATCTAATTTGAATAAATAAACGATAACCGCGTTGGTTTTAGAGGGCATAATAAAAGAAATATTGTTATCGTGTTCGCCATTAGGGCAGTATAAATTGACGTTCATGTTGGATATTGTTTCAGTTGCAAGTTGACACTGGCGGCGACAAGAGATCTGCCGGGCTGCAGGAATTCGATATCAAGCTTATCGATGGGATCCGTGAGCAAGGGCGAGGAGCTGTTCACCGGGGTGGTGCCCATCCTGGTCGAGCTGGACGGCGACGTAAACGGCCACAAGTTCAGCGTGTCCGGCGAGGGCGAGGGCGATGCCACCTACGGCAAGCTGACCCTGAAGTTCATCTGCACCACCGGCAAGCTGCCCGTGCCCTGGCCCACCCTCGTGACCACCCTGACCTACGGCGTGCAGTGCTTCAGCCGCTACCCCGACCACATGAAGCAGCACGACTTCTTCAAGTCCGCCATGCCCGAAGGCTACGTCCAGGAGCGCACCATCTTCTTCAAGGACGACGGCAACTACAAGACCCGCGCCGAGGTGAAGTTCGAGGGCGACACCCTGGTGAACCGCATCGAGCTGAAGGGCATCGACTTCAAGGAGGACGGCAACATCCTGGGGCACAAGCTGGAGTACAACTACAACAGCCACAACGTCTATATCATGGCCGACAAGCAGAAGAACGGCATCAAGGTGAACTTCAAGATCCGCCACAACATCGAGGACGGCAGCGTGCAGCTCGCCGACCACTACCAGCAGAACACCCCCATCGGCGACGGCCCCGTGCTGCTGCCCGACAACCACTACCTGAGCACCCAGTCCGCCCTGAGCAAAGACCCCAACGAGAAGCGCGATCACATGGTCCTGCTGGAGTTCGTGACCGCCGCCGGGATCACTCTCGGCATGGACGAGCTGTACAAGTCCTACCCATACGACGTCCCAGACTACGCACCGTCGTACCGGGTTAAACGCATGGACATCGCAAAGAACGATGAAGAGTGTGTAGTCAACGCCGCCAACCCTCGTGGGCTACCAGGCGATGGCGTCTGTAAAGCAGTATACAAAAAATGGCCGGAGTCCTTCAAGAACAGTGCAACACCAGTGGGAACCGCAAAGACAGTCATGTGCGGTACATACCCGGTAATCCATGCAGTAGGACCTAATTTCTCAAATTACTCTGAGTCCGAAGGAGACCGGGAATTGGCAGCTGCTTACCGAGAAGTCGCTAAGGAGGTGACTAGACTAGGAGTAAACAGCGTAGCTATACCGCTCCTTTCCACCGGTGTGTACTCTGGAGGGAAAGACAGGCTGACTCAGTCACTAAACCACCTTTTTACAGCATTAGACTCAACTGATGCAGATGTGGTTATCTACTGCCGCGACAAGGAGTGGGAGAAGAAAATAGCTGAGGCCATACAAATGAGGACCCAAGTGGAATTACTAGACGAACACATCTCTGTAGACTGCGATATCATCCGAGTGCACCCTGACAGCAGTTTGGCAGGTAGAAAAGGGTACAGCACTACAGAAGGTTCACTGTACTCCTACTTGGAAGGGACACGGTTCCATCAGACGGCAGTGGACATGGCAGAAGTATACACCATGTGGCCAAAGCAGACGGAGGCTAATGAACAAGTTTGCTTGTACGCATTGGGGGAAAGTATAGAATCAATCAGGCAAAAGTGCCCAGTGGATGACGCAGATGCATCGTCGCCCCCAAAAACCGTCCCGTGCCTCTGCCGTTATGCCATGACACCCGAACGAGTCACCAGGCTTCGTATGAACCATGTCACAAGCATAATAGTATGCTCATCATTCCCCCTTCCAAAGTATAAAATAGAAGGAGTGCAGAAAGTCAAGTGTTCTAAAGTGATGCTGTTCGACCATAACGTGCCATCACGCGTTAGTCCAAGGGAATATAAATCGCCTCAGGAGACCGCACAAGAAGTAAGTTCGACCACGTCACTGACGCACAGCCAATTCGACCTTAGCGTTGACGGTGAGGAACTGCCCGCTCCGTCTGACTTGGAAGCTGACGCTCCGATTCCGGAACCAACACCAGACGACAGAGCGGTACTTACTTTGCCTCCCACGATTGATAATTTTTCGGCTGTGTCAGACTGGGTAATGAATACCGCGCCAGTCGCACCACCCAGAAGAAGACGTGGGAAAAACTTGAATGTCACCTGCGACGAGAGAGAAGGGAACGTACTTCCCATGGCTAGCGTTCGGTTCTTCAGAGCGGATCTGCACTCCATCGTACAGGAAACGGCAGAGATACGCGATACGGCCGCGTCCCTCCAGGCGCCCCTGAGTGTCGCTACAGAACCGAATCAACTGCCGATCTCATTTGGAGCACCAAACGAGACTTTCCCCATAACGTTCGGGGATTTTGATGAAGGGGAGATTGAAAGCTTGTCCTCTGAGTTACTGACCTTTGGGGACTTCTCGCCGGGCGAAGTGGATGACCTGACAGACAGCGACTGGTCCACGTGTTCAGACACGGACGACGAATTATGACTAGATAGGGCAGGTGGGTAGGTCGACCTCGACTAGTGCACTCAACAAAAATGTAATATTAAACACAATTAAATAAATGTTAAAATTTATTGCCTAATATTATTTTGTCATTGCTTGTCATTTATTAATTTGGATGATGTCATTTGTTTTTAAAATTGAACTGGCTTTACGAGTAGAATTCGAGCTCCAATTCGCCCTATAGTGAGTCGTATTACAATTCACTGGCCGTCGTTTTACAACGTCGTGACTGGGAAAACCCTGGCGTTACCCAACTTAATCGCCTTGCAGCACATCCCCCTTTCGCCAGCTGGCGTAATAGCGAAGAGGCCCGCACCGATCGCCCTTCCCAACAGTTGCGCAGCCTGAATGGCGAATGGCGCGACGCGCCCTGTAGCGGCGCATTAAGCGCGGCGGGTGTGGTGGTTACGCGCAGCGTGACCGCTACACTTGCCAGCGCCCTAGCGCCCGCTCCTTTCGCTTTCTTCCCTTCCTTTCTCGCCACGTTCGCCGGCTTTCCCCGTCAAGCTCTAAATCGGGGGCTCCCTTTAGGGTTCCGATTTAGTGCTTTACGGCACCTCGACCCCAAAAAACTTGATTAGGGTGATGGTTCACGTAGTGGGCCATCGCCCTGATAGACGGTTTTTCGCCCTTTGACGTTGGAGTCCACGTTCTTTAATAGTGGACTCTTGTTCCAAACTGGAACAACACTCAACCCTATCTCGGTCTATTCTTTTGATTTATAAGGGATTTTGCCGATTTCGGCCTATTGGTTAAAAAATGAGCTGATTTAACAAAAATTTAACGCGAATTTTAACAAAATATTAACGTTTACAATTTCCCAGGTGGCACTTTTCGGGGAAATGTGCGCGGAACCCCTATTTGTTTATTTTTCTAAATACATTCAAATATGTATCCGCTCATGAGACAATAACCCTGATAAATGCTTCAATAATATTGAAAAAGGAAGAGTATGAGTATTCAACATTTCCGTGTCGCCCTTATTCCCTTTTTTGCGGCATTTTGCCTTCCTGTTTTTGCTCACCCAGAAACGCTGGTGAAAGTAAAAGATGCTGAAGATCAGTTGGGTGCACGAGTGGGTTACATCGAACTGGATCTCAACAGCGGTAAGATCCTTGAGAGTTTTCGCCCCGAAGAACGTTTTCCAATGATGAGCACTTTTAAAGTTCTGCTATGTGGCGCGGTATTATCCCGTATTGACGCCGGGCAAGAGCAACTCGGTCGCCGCATACACTATTCTCAGAATGACTTGGTTGAGTACTCACCAGTCACAGAAAAGCATCTTACGGATGGCATGACAGTAAGAGAATTATGCAGTGCTGCCATAACCATGAGTGATAACACTGCGGCCAACTTACTTCTGACAACGATCGGAGGACCGAAGGAGCTAACCGCTTTTTTGCACAACATGGGGGATCATGTAACTCGCCTTGATCGTTGGGAACCGGAGCTGAATGAAGCCATACCAAACGACGAGCGTGACACCACGATGCCTGTAGCAATGGCAACAACGTTGCGCAAACTATTAACTGGCGAACTACTTACTCTAGCTTCCCGGCAACAATTAATAGACTGGATGGAGGCGGATAAAGTTGCAGGACCACTTCTGCGCTCGGCCCTTCCGGCTGGCTGGTTTATTGCTGATAAATCTGGAGCCGGTGAGCGTGGGTCTCGCGGTATCATTGCAGCACTGGGGCCAGATGGTAAGCCCTCCCGTATCGTAGTTATCTACACGACGGGGAGTCAGGCAACTATGGATGAACGAAATAGACAGATCGCTGAGATAGGTGCCTCACTGATTAAGCATTGGTAACTGTCAGACCAAGTTTACTCATATATACTTTAGATTGATTTAAAACTTCATTTTTAATTTAAAAGGATCTAGGTGAAGATCCTTTTTGATAATCTCATGACCAAAATCCCTTAACGTGAGTTTTCGTTCCACTGAGCGTCAGACCCCGTAGAAAAGATCAAAGGATCTTCTTGAGATCCTTTTTTTCTGCGCGTAATCTGCTGCTTGCAAACAAAAAAACCACCGCTACCAGCGGTGGTTTGTTTGCCGGATCAAGAGCTACCAACTCTTTTTCCGAAGGTAACTGGCTTCAGCAGAGCGCAGATACCAAATACTGTCCTTCTAGTGTAGCCGTAGTTAGGCCACCACTTCAAGAACTCTGTAGCACCGCCTACATACCTCGCTCTGCTAATCCTGTTACCAGTGGCTGCTGCCAGTGGCGATAAGTCGTGTCTTACCGGGTTGGACTCAAGACGATAGTTACCGGATAAGGCGCAGCGGTCGGGCTGAACGGGGGGTTCGTGCACACAGCCCAGCTTGGAGCGAACGACCTACACCGAACTGAGATACCTACAGCGTGAGCTATGAGAAAGCGCCACGCTTCCCGAAGGGAGAAAGGCGGACAGGTATCCGGTAAGCGGCAGGGTCGGAACAGGAGAGCGCACGAGGGAGCTTCCAGGGGGAAACGCCTGGTATCTTTATAGTCCTGTCGGGTTTCGCCACCTCTGACTTGAGCGTCGATTTTTGTGATGCTCGTCAGGGGGGCGGAGCCTATGGAAAAACGCCAGCAACGCGGCCTTTTTACGGTTCCTGGCCTTTTGCTGGCCTTTTGCTCACATGTTCTTTCCTGCGTTATCCCCTGATTCTGTGGATAACCGTATTACCGCCTTTGAGTGAGCTGATACCGCTCGCCGCAGCCGAACGACCGAGCGCAGCGAGTCAGTGAGCGAGGAAGCGGAAGAGCGCCCAATACGCAAACCGCCTCTCCCCGCGCGTTGGCCGATTCATTAATGCAGCTGGCACGACAGGTTTCCCGACTGGAAAGCGGGCAGTGAGCGCAACGCAATTAATGTGAGTTAGCTCACTCATTAGGCACCCCAGGCTTTACACTTTATGCTTCCGGCTCGTATGTTGTGTGGAATTGTGAGCGGATAACAATTTCACACAGGAAACAGCTATGACCATGATTACGCCAAGCTCGGAATTAACCCTCACTAAAGGGAACAAAAGCTGGGTAC

>pIE1-hr5-GFP-HA-CHIKnsP3 arg PA

CGGGCCCCCCCTCGAGGGGGATCCGGCGCGTAAAACACAATCAAGTATGAGTCATAAGCTGATGTCATGTTTTGCACACGGCTCATAACCGAACTGGCTTTACGAGTAGAATTCTACTTGTAACGCACGATCAGTGGATGATGGTCATTTGTTTTTCAAATCGAGATGATGTCATGTTTTGCACACGGGCTCATAAACTGCTTTACGAGTAGAATTCTACGTGTAACGCACGATCGAGATGAGTCATTTGTTTTGCAATATGATATCATACAATATGACTCATTTGTTTTTCAAAACCGAACTTGATTTACGGGTAGAATTCTACTCGTAAAGCACAATCAAAAAGATGATGTCATTTGTTTTTCAAAACTGAACTCTCGGCTTTACGAGTAGAATTCTACGTGTAAAACACAATCAAGAAATGATGTCATTTGTTATAAAAATAAAAGCTGATGTCATGTTTTGCACATGGCTCATAACTAAACTCGCTTTACGGGTAGAATTCTACGCGCCGGATCCACTAGCTAGTTCTAGAGTCGCGATGTCTTTGTGATGCGCCGACATTTTTGTAGGTTATTGATAAAATGAACGGATACAGTTGCCCGACATTATCATTAAATCCTTGGCGTAGAATTTGTCGGGTCCATTGTCCGTGTGCGCTAGCATGCCCGCTAACGGACCTCGTACTTTTGGCTTCAAAGGTTTTGCGCACAGACAAAATGTGCCACACTTGCAGCTCTGCATGTGTGCGCGTTACCACAAATCCCAACGGCGCAGTGTACTTGTTGTATGCAAATAAATCTCGATAAAGGCGCGGCGCGCGAATGCAGCTGATCACGTACGCTCCTCGTGTTCCGTTCAAGGACGGTGTTATCGACCTCAGATTAATGTTTATCGGCCGACTGTTTTCGTATCCGCTCACCAAACGCGTTTTTGCATTAACATTGTATGTCGGCGGATGTTCTATATCTAATTTGAATAAATAAACGATAACCGCGTTGGTTTTAGAGGGCATAATAAAAGAAATATTGTTATCGTGTTCGCCATTAGGGCAGTATAAATTGACGTTCATGTTGGATATTGTTTCAGTTGCAAGTTGACACTGGCGGCGACAAGAGATCTGCCGGGCTGCAGGAATTCGATATCAAGCTTATCGATGGGATCCGTGAGCAAGGGCGAGGAGCTGTTCACCGGGGTGGTGCCCATCCTGGTCGAGCTGGACGGCGACGTAAACGGCCACAAGTTCAGCGTGTCCGGCGAGGGCGAGGGCGATGCCACCTACGGCAAGCTGACCCTGAAGTTCATCTGCACCACCGGCAAGCTGCCCGTGCCCTGGCCCACCCTCGTGACCACCCTGACCTACGGCGTGCAGTGCTTCAGCCGCTACCCCGACCACATGAAGCAGCACGACTTCTTCAAGTCCGCCATGCCCGAAGGCTACGTCCAGGAGCGCACCATCTTCTTCAAGGACGACGGCAACTACAAGACCCGCGCCGAGGTGAAGTTCGAGGGCGACACCCTGGTGAACCGCATCGAGCTGAAGGGCATCGACTTCAAGGAGGACGGCAACATCCTGGGGCACAAGCTGGAGTACAACTACAACAGCCACAACGTCTATATCATGGCCGACAAGCAGAAGAACGGCATCAAGGTGAACTTCAAGATCCGCCACAACATCGAGGACGGCAGCGTGCAGCTCGCCGACCACTACCAGCAGAACACCCCCATCGGCGACGGCCCCGTGCTGCTGCCCGACAACCACTACCTGAGCACCCAGTCCGCCCTGAGCAAAGACCCCAACGAGAAGCGCGATCACATGGTCCTGCTGGAGTTCGTGACCGCCGCCGGGATCACTCTCGGCATGGACGAGCTGTACAAGTCCTACCCATACGACGTCCCAGACTACGCACCGTCGTACCGGGTTAAACGCATGGACATCGCAAAGAACGATGAAGAGTGTGTAGTCAACGCCGCCAACCCTCGTGGGCTACCAGGCGATGGCGTCTGTAAAGCAGTATACAAAAAATGGCCGGAGTCCTTCAAGAACAGTGCAACACCAGTGGGAACCGCAAAGACAGTCATGTGCGGTACATACCCGGTAATCCATGCAGTAGGACCTAATTTCTCAAATTACTCTGAGTCCGAAGGAGACCGGGAATTGGCAGCTGCTTACCGAGAAGTCGCTAAGGAGGTGACTAGACTAGGAGTAAACAGCGTAGCTATACCGCTCCTTTCCACCGGTGTGTACTCTGGAGGGAAAGACAGGCTGACTCAGTCACTAAACCACCTTTTTACAGCATTAGACTCAACTGATGCAGATGTGGTTATCTACTGCCGCGACAAGGAGTGGGAGAAGAAAATAGCTGAGGCCATACAAATGAGGACCCAAGTGGAATTACTAGACGAACACATCTCTGTAGACTGCGATATCATCCGAGTGCACCCTGACAGCAGTTTGGCAGGTAGAAAAGGGTACAGCACTACAGAAGGTTCACTGTACTCCTACTTGGAAGGGACACGGTTCCATCAGACGGCAGTGGACATGGCAGAAGTATACACCATGTGGCCAAAGCAGACGGAGGCTAATGAACAAGTTTGCTTGTACGCATTGGGGGAAAGTATAGAATCAATCAGGCAAAAGTGCCCAGTGGATGACGCAGATGCATCGTCGCCCCCAAAAACCGTCCCGTGCCTCTGCCGTTATGCCATGACACCCGAACGAGTCACCAGGCTTCGTATGAACCATGTCACAAGCATAATAGTATGCTCATCATTCCCCCTTCCAAAGTATAAAATAGAAGGAGTGCAGAAAGTCAAGTGTTCTAAAGTGATGCTGTTCGACCATAACGTGCCATCACGCGTTAGTCCAAGGGAATATAAATCGCCTCAGGAGACCGCACAAGAAGTAAGTTCGACCACGTCACTGACGCACAGCCAATTCGACCTTAGCGTTGACGGTGAGGAACTGCCCGCTCCGTCTGACTTGGAAGCTGACGCTCCGATTCCGGAACCAACACCAGACGACAGAGCGGTACTTACTTTGCCTCCCACGATTGATAATTTTTCGGCTGTGTCAGACTGGGTAATGAATACCGCGCCAGTCGCACCACCCAGAAGAAGACGTGGGAAAAACTTGAATGTCACCTGCGACGAGAGAGAAGGGAACGTACTTCCCATGGCTAGCGTTCGGTTCTTCAGAGCGGATCTGCACTCCATCGTACAGGAAACGGCAGAGATACGCGATACGGCCGCGTCCCTCCAGGCGCCCCTGAGTGTCGCTACAGAACCGAATCAACTGCCGATCTCATTTGGAGCACCAAACGAGACTTTCCCCATAACGTTCGGGGATTTTGATGAAGGGGAGATTGAAAGCTTGTCCTCTGAGTTACTGACCTTTGGGGACTTCTCGCCGGGCGAAGTGGATGACCTGACAGACAGCGACTGGTCCACGTGTTCAGACACGGACGACGAATTACGACTAGATAGGGCAGGTGGGTAGGTCGACCTCGACTAGTGCACTCAACAAAAATGTAATATTAAACACAATTAAATAAATGTTAAAATTTATTGCCTAATATTATTTTGTCATTGCTTGTCATTTATTAATTTGGATGATGTCATTTGTTTTTAAAATTGAACTGGCTTTACGAGTAGAATTCGAGCTCCAATTCGCCCTATAGTGAGTCGTATTACAATTCACTGGCCGTCGTTTTACAACGTCGTGACTGGGAAAACCCTGGCGTTACCCAACTTAATCGCCTTGCAGCACATCCCCCTTTCGCCAGCTGGCGTAATAGCGAAGAGGCCCGCACCGATCGCCCTTCCCAACAGTTGCGCAGCCTGAATGGCGAATGGCGCGACGCGCCCTGTAGCGGCGCATTAAGCGCGGCGGGTGTGGTGGTTACGCGCAGCGTGACCGCTACACTTGCCAGCGCCCTAGCGCCCGCTCCTTTCGCTTTCTTCCCTTCCTTTCTCGCCACGTTCGCCGGCTTTCCCCGTCAAGCTCTAAATCGGGGGCTCCCTTTAGGGTTCCGATTTAGTGCTTTACGGCACCTCGACCCCAAAAAACTTGATTAGGGTGATGGTTCACGTAGTGGGCCATCGCCCTGATAGACGGTTTTTCGCCCTTTGACGTTGGAGTCCACGTTCTTTAATAGTGGACTCTTGTTCCAAACTGGAACAACACTCAACCCTATCTCGGTCTATTCTTTTGATTTATAAGGGATTTTGCCGATTTCGGCCTATTGGTTAAAAAATGAGCTGATTTAACAAAAATTTAACGCGAATTTTAACAAAATATTAACGTTTACAATTTCCCAGGTGGCACTTTTCGGGGAAATGTGCGCGGAACCCCTATTTGTTTATTTTTCTAAATACATTCAAATATGTATCCGCTCATGAGACAATAACCCTGATAAATGCTTCAATAATATTGAAAAAGGAAGAGTATGAGTATTCAACATTTCCGTGTCGCCCTTATTCCCTTTTTTGCGGCATTTTGCCTTCCTGTTTTTGCTCACCCAGAAACGCTGGTGAAAGTAAAAGATGCTGAAGATCAGTTGGGTGCACGAGTGGGTTACATCGAACTGGATCTCAACAGCGGTAAGATCCTTGAGAGTTTTCGCCCCGAAGAACGTTTTCCAATGATGAGCACTTTTAAAGTTCTGCTATGTGGCGCGGTATTATCCCGTATTGACGCCGGGCAAGAGCAACTCGGTCGCCGCATACACTATTCTCAGAATGACTTGGTTGAGTACTCACCAGTCACAGAAAAGCATCTTACGGATGGCATGACAGTAAGAGAATTATGCAGTGCTGCCATAACCATGAGTGATAACACTGCGGCCAACTTACTTCTGACAACGATCGGAGGACCGAAGGAGCTAACCGCTTTTTTGCACAACATGGGGGATCATGTAACTCGCCTTGATCGTTGGGAACCGGAGCTGAATGAAGCCATACCAAACGACGAGCGTGACACCACGATGCCTGTAGCAATGGCAACAACGTTGCGCAAACTATTAACTGGCGAACTACTTACTCTAGCTTCCCGGCAACAATTAATAGACTGGATGGAGGCGGATAAAGTTGCAGGACCACTTCTGCGCTCGGCCCTTCCGGCTGGCTGGTTTATTGCTGATAAATCTGGAGCCGGTGAGCGTGGGTCTCGCGGTATCATTGCAGCACTGGGGCCAGATGGTAAGCCCTCCCGTATCGTAGTTATCTACACGACGGGGAGTCAGGCAACTATGGATGAACGAAATAGACAGATCGCTGAGATAGGTGCCTCACTGATTAAGCATTGGTAACTGTCAGACCAAGTTTACTCATATATACTTTAGATTGATTTAAAACTTCATTTTTAATTTAAAAGGATCTAGGTGAAGATCCTTTTTGATAATCTCATGACCAAAATCCCTTAACGTGAGTTTTCGTTCCACTGAGCGTCAGACCCCGTAGAAAAGATCAAAGGATCTTCTTGAGATCCTTTTTTTCTGCGCGTAATCTGCTGCTTGCAAACAAAAAAACCACCGCTACCAGCGGTGGTTTGTTTGCCGGATCAAGAGCTACCAACTCTTTTTCCGAAGGTAACTGGCTTCAGCAGAGCGCAGATACCAAATACTGTCCTTCTAGTGTAGCCGTAGTTAGGCCACCACTTCAAGAACTCTGTAGCACCGCCTACATACCTCGCTCTGCTAATCCTGTTACCAGTGGCTGCTGCCAGTGGCGATAAGTCGTGTCTTACCGGGTTGGACTCAAGACGATAGTTACCGGATAAGGCGCAGCGGTCGGGCTGAACGGGGGGTTCGTGCACACAGCCCAGCTTGGAGCGAACGACCTACACCGAACTGAGATACCTACAGCGTGAGCTATGAGAAAGCGCCACGCTTCCCGAAGGGAGAAAGGCGGACAGGTATCCGGTAAGCGGCAGGGTCGGAACAGGAGAGCGCACGAGGGAGCTTCCAGGGGGAAACGCCTGGTATCTTTATAGTCCTGTCGGGTTTCGCCACCTCTGACTTGAGCGTCGATTTTTGTGATGCTCGTCAGGGGGGCGGAGCCTATGGAAAAACGCCAGCAACGCGGCCTTTTTACGGTTCCTGGCCTTTTGCTGGCCTTTTGCTCACATGTTCTTTCCTGCGTTATCCCCTGATTCTGTGGATAACCGTATTACCGCCTTTGAGTGAGCTGATACCGCTCGCCGCAGCCGAACGACCGAGCGCAGCGAGTCAGTGAGCGAGGAAGCGGAAGAGCGCCCAATACGCAAACCGCCTCTCCCCGCGCGTTGGCCGATTCATTAATGCAGCTGGCACGACAGGTTTCCCGACTGGAAAGCGGGCAGTGAGCGCAACGCAATTAATGTGAGTTAGCTCACTCATTAGGCACCCCAGGCTTTACACTTTATGCTTCCGGCTCGTATGTTGTGTGGAATTGTGAGCGGATAACAATTTCACACAGGAAACAGCTATGACCATGATTACGCCAAGCTCGGAATTAACCCTCACTAAAGGGAACAAAAGCTGGGTAC

>pIE1-hr5-GFP-HA-ONNnsP3 opal PA

CGGGCCCCCCCTCGAGGGGGATCCGGCGCGTAAAACACAATCAAGTATGAGTCATAAGCTGATGTCATGTTTTGCACACGGCTCATAACCGAACTGGCTTTACGAGTAGAATTCTACTTGTAACGCACGATCAGTGGATGATGGTCATTTGTTTTTCAAATCGAGATGATGTCATGTTTTGCACACGGGCTCATAAACTGCTTTACGAGTAGAATTCTACGTGTAACGCACGATCGAGATGAGTCATTTGTTTTGCAATATGATATCATACAATATGACTCATTTGTTTTTCAAAACCGAACTTGATTTACGGGTAGAATTCTACTCGTAAAGCACAATCAAAAAGATGATGTCATTTGTTTTTCAAAACTGAACTCTCGGCTTTACGAGTAGAATTCTACGTGTAAAACACAATCAAGAAATGATGTCATTTGTTATAAAAATAAAAGCTGATGTCATGTTTTGCACATGGCTCATAACTAAACTCGCTTTACGGGTAGAATTCTACGCGCCGGATCCACTAGCTAGTTCTAGAGTCGCGATGTCTTTGTGATGCGCCGACATTTTTGTAGGTTATTGATAAAATGAACGGATACAGTTGCCCGACATTATCATTAAATCCTTGGCGTAGAATTTGTCGGGTCCATTGTCCGTGTGCGCTAGCATGCCCGCTAACGGACCTCGTACTTTTGGCTTCAAAGGTTTTGCGCACAGACAAAATGTGCCACACTTGCAGCTCTGCATGTGTGCGCGTTACCACAAATCCCAACGGCGCAGTGTACTTGTTGTATGCAAATAAATCTCGATAAAGGCGCGGCGCGCGAATGCAGCTGATCACGTACGCTCCTCGTGTTCCGTTCAAGGACGGTGTTATCGACCTCAGATTAATGTTTATCGGCCGACTGTTTTCGTATCCGCTCACCAAACGCGTTTTTGCATTAACATTGTATGTCGGCGGATGTTCTATATCTAATTTGAATAAATAAACGATAACCGCGTTGGTTTTAGAGGGCATAATAAAAGAAATATTGTTATCGTGTTCGCCATTAGGGCAGTATAAATTGACGTTCATGTTGGATATTGTTTCAGTTGCAAGTTGACACTGGCGGCGACAAGAGATCTGCCGGGCTGCAGGAATTCGATATCAAGCTTATCGATGGGATCCGTGAGCAAGGGCGAGGAGCTGTTCACCGGGGTGGTGCCCATCCTGGTCGAGCTGGACGGCGACGTAAACGGCCACAAGTTCAGCGTGTCCGGCGAGGGCGAGGGCGATGCCACCTACGGCAAGCTGACCCTGAAGTTCATCTGCACCACCGGCAAGCTGCCCGTGCCCTGGCCCACCCTCGTGACCACCCTGACCTACGGCGTGCAGTGCTTCAGCCGCTACCCCGACCACATGAAGCAGCACGACTTCTTCAAGTCCGCCATGCCCGAAGGCTACGTCCAGGAGCGCACCATCTTCTTCAAGGACGACGGCAACTACAAGACCCGCGCCGAGGTGAAGTTCGAGGGCGACACCCTGGTGAACCGCATCGAGCTGAAGGGCATCGACTTCAAGGAGGACGGCAACATCCTGGGGCACAAGCTGGAGTACAACTACAACAGCCACAACGTCTATATCATGGCCGACAAGCAGAAGAACGGCATCAAGGTGAACTTCAAGATCCGCCACAACATCGAGGACGGCAGCGTGCAGCTCGCCGACCACTACCAGCAGAACACCCCCATCGGCGACGGCCCCGTGCTGCTGCCCGACAACCACTACCTGAGCACCCAGTCCGCCCTGAGCAAAGACCCCAACGAGAAGCGCGATCACATGGTCCTGCTGGAGTTCGTGACCGCCGCCGGGATCACTCTCGGCATGGACGAGCTGTACAAGTCCTACCCATACGACGTCCCAGACTACGCTCCGTCATACCGTGTGAAACGGATGGACATCGCGAAAAACACTGAGGAATGCGTGGTAAACGCCGCCAATCCACGCGGAGTACCAGGCGATGGAGTATGTAAAGCCGTGTATAGAAAATGGCCAGAATCATTCAGAAACAGTGCAACGCCAGTGGGTACTGCAAAGACAATCATGTGCGGTCAATACCCCGTCATCCACGCGGTAGGCCCAAACTTCTCAAACTATTCTGAGGCTGAAGGGGATAGGGAATTGGCTTCAGTGTATAGAGAAGTGGCGAAAGAAGTGTCTAGGCTAGGAGTGAGCAGTGTAGCCATCCCTTTGCTTTCAACCGGTGTGTACTCAGGAGGCAAAGATAGACTGCTGCAATCACTAAACCATCTTTTCACAGCGATGGATTCGACAGATGCAGATGTTGTCATCTATTGCAGGGATAAGGAATGGGAGAAGAAGATCACTGAAGCCATATCATTAAGATCCCAGGTAGAACTACTAGATGATCACATCTCAGTGGATTGCGACATTGTACGCGTTCATCCAGACAGCAGCTTGGCAGGCCGAAAGGGGTACAGCACAGTAGAGGGAGCACTCTACTCGTACCTAGAGGGAACAAGATTCCACCAAACTGCTGTAGATATGGCAGAAATATATACCATGTGGCCAAAACAAACTGAAGCCAATGAACAGGTCTGCCTATATGCTCTGGGGGAGAGCATAGAGTCAGTCAGGCAAAAATGTCCTGTAGACGACGCCGACGCCTCATTCCCTCCGAAAACAGTCCCGTGCCTATGCCGTTATGCCATGACGCCTGAACGAGTTGCACGCCTACGCATGAATCATACTACTAGCATCATAGTGTGCTCGTCTTTTCCACTGCCGAAGTACAAAATCGAGGGCGTGCAAAAAGTAAAATGTTCAAAAGCACTCTTGTTTGATCACAACGTACCGTCTCGAGTGAGCCCGAGAACGTACAGGCCTGCGGACGAAATCATACAGACACCTCAAATACCAACTGAAGCGTGCCAGGACGCACAATTCGTGCAGTCAATAACTGATGAAGCAGTGCCAGTTCCGTCAGACTTAGAGGCTTGTGACGCAACTATGGACTGGCCCTCTATCGACATCGTACCAACAAGACAAAGAAGCGACTCATTTGACAGCGAGTATAGTTCCAGAAGTAACATACAGCTGGTGACAGCGGACGTGCATGCACCAATGTACGCAAATTCGCTGGCGTCCAGCGGAGGTTCAGTGCTGTCGCTGTCCAGTGAACAAGCTCAGAACGGCATAATGATACTACCTGATTCAGAAGACACAGATAGTATAAGCAGAGTAAGCACACCGATCGCCCCACCCAGGAGACGTTTGGGAAGGACTATAAATGTGACTTGTGACGAGCGGGAAGGGAAAATACTCCCTATGGCCAGCGACAGGCTCTTCACTGCTAAGCCATACACTGTCGCACTGGGCGTATCAACAGCAGACATAACTGCGTACCCCATCCAGGCACCGCTAGGATCGACACAACCGCCTGCCCTCGAACAGATCACTTTCGGAGATTTCGCCGAAGGTGAAATAGACAACCTCCTGACAGGGGCATTGACATTTGGAGACTTCGAGCCAGGTGAAGTGGAAGAGCTGACGGATAGCGAGTGGTCAACATGCTCGGACACAGATGAAGAGTTATGAGTCGACCTCGACTAGTGCACTCAACAAAAATGTAATATTAAACACAATTAAATAAATGTTAAAATTTATTGCCTAATATTATTTTGTCATTGCTTGTCATTTATTAATTTGGATGATGTCATTTGTTTTTAAAATTGAACTGGCTTTACGAGTAGAATTCGAGCTCCAATTCGCCCTATAGTGAGTCGTATTACAATTCACTGGCCGTCGTTTTACAACGTCGTGACTGGGAAAACCCTGGCGTTACCCAACTTAATCGCCTTGCAGCACATCCCCCTTTCGCCAGCTGGCGTAATAGCGAAGAGGCCCGCACCGATCGCCCTTCCCAACAGTTGCGCAGCCTGAATGGCGAATGGCGCGACGCGCCCTGTAGCGGCGCATTAAGCGCGGCGGGTGTGGTGGTTACGCGCAGCGTGACCGCTACACTTGCCAGCGCCCTAGCGCCCGCTCCTTTCGCTTTCTTCCCTTCCTTTCTCGCCACGTTCGCCGGCTTTCCCCGTCAAGCTCTAAATCGGGGGCTCCCTTTAGGGTTCCGATTTAGTGCTTTACGGCACCTCGACCCCAAAAAACTTGATTAGGGTGATGGTTCACGTAGTGGGCCATCGCCCTGATAGACGGTTTTTCGCCCTTTGACGTTGGAGTCCACGTTCTTTAATAGTGGACTCTTGTTCCAAACTGGAACAACACTCAACCCTATCTCGGTCTATTCTTTTGATTTATAAGGGATTTTGCCGATTTCGGCCTATTGGTTAAAAAATGAGCTGATTTAACAAAAATTTAACGCGAATTTTAACAAAATATTAACGTTTACAATTTCCCAGGTGGCACTTTTCGGGGAAATGTGCGCGGAACCCCTATTTGTTTATTTTTCTAAATACATTCAAATATGTATCCGCTCATGAGACAATAACCCTGATAAATGCTTCAATAATATTGAAAAAGGAAGAGTATGAGTATTCAACATTTCCGTGTCGCCCTTATTCCCTTTTTTGCGGCATTTTGCCTTCCTGTTTTTGCTCACCCAGAAACGCTGGTGAAAGTAAAAGATGCTGAAGATCAGTTGGGTGCACGAGTGGGTTACATCGAACTGGATCTCAACAGCGGTAAGATCCTTGAGAGTTTTCGCCCCGAAGAACGTTTTCCAATGATGAGCACTTTTAAAGTTCTGCTATGTGGCGCGGTATTATCCCGTATTGACGCCGGGCAAGAGCAACTCGGTCGCCGCATACACTATTCTCAGAATGACTTGGTTGAGTACTCACCAGTCACAGAAAAGCATCTTACGGATGGCATGACAGTAAGAGAATTATGCAGTGCTGCCATAACCATGAGTGATAACACTGCGGCCAACTTACTTCTGACAACGATCGGAGGACCGAAGGAGCTAACCGCTTTTTTGCACAACATGGGGGATCATGTAACTCGCCTTGATCGTTGGGAACCGGAGCTGAATGAAGCCATACCAAACGACGAGCGTGACACCACGATGCCTGTAGCAATGGCAACAACGTTGCGCAAACTATTAACTGGCGAACTACTTACTCTAGCTTCCCGGCAACAATTAATAGACTGGATGGAGGCGGATAAAGTTGCAGGACCACTTCTGCGCTCGGCCCTTCCGGCTGGCTGGTTTATTGCTGATAAATCTGGAGCCGGTGAGCGTGGGTCTCGCGGTATCATTGCAGCACTGGGGCCAGATGGTAAGCCCTCCCGTATCGTAGTTATCTACACGACGGGGAGTCAGGCAACTATGGATGAACGAAATAGACAGATCGCTGAGATAGGTGCCTCACTGATTAAGCATTGGTAACTGTCAGACCAAGTTTACTCATATATACTTTAGATTGATTTAAAACTTCATTTTTAATTTAAAAGGATCTAGGTGAAGATCCTTTTTGATAATCTCATGACCAAAATCCCTTAACGTGAGTTTTCGTTCCACTGAGCGTCAGACCCCGTAGAAAAGATCAAAGGATCTTCTTGAGATCCTTTTTTTCTGCGCGTAATCTGCTGCTTGCAAACAAAAAAACCACCGCTACCAGCGGTGGTTTGTTTGCCGGATCAAGAGCTACCAACTCTTTTTCCGAAGGTAACTGGCTTCAGCAGAGCGCAGATACCAAATACTGTCCTTCTAGTGTAGCCGTAGTTAGGCCACCACTTCAAGAACTCTGTAGCACCGCCTACATACCTCGCTCTGCTAATCCTGTTACCAGTGGCTGCTGCCAGTGGCGATAAGTCGTGTCTTACCGGGTTGGACTCAAGACGATAGTTACCGGATAAGGCGCAGCGGTCGGGCTGAACGGGGGGTTCGTGCACACAGCCCAGCTTGGAGCGAACGACCTACACCGAACTGAGATACCTACAGCGTGAGCTATGAGAAAGCGCCACGCTTCCCGAAGGGAGAAAGGCGGACAGGTATCCGGTAAGCGGCAGGGTCGGAACAGGAGAGCGCACGAGGGAGCTTCCAGGGGGAAACGCCTGGTATCTTTATAGTCCTGTCGGGTTTCGCCACCTCTGACTTGAGCGTCGATTTTTGTGATGCTCGTCAGGGGGGCGGAGCCTATGGAAAAACGCCAGCAACGCGGCCTTTTTACGGTTCCTGGCCTTTTGCTGGCCTTTTGCTCACATGTTCTTTCCTGCGTTATCCCCTGATTCTGTGGATAACCGTATTACCGCCTTTGAGTGAGCTGATACCGCTCGCCGCAGCCGAACGACCGAGCGCAGCGAGTCAGTGAGCGAGGAAGCGGAAGAGCGCCCAATACGCAAACCGCCTCTCCCCGCGCGTTGGCCGATTCATTAATGCAGCTGGCACGACAGGTTTCCCGACTGGAAAGCGGGCAGTGAGCGCAACGCAATTAATGTGAGTTAGCTCACTCATTAGGCACCCCAGGCTTTACACTTTATGCTTCCGGCTCGTATGTTGTGTGGAATTGTGAGCGGATAACAATTTCACACAGGAAACAGCTATGACCATGATTACGCCAAGCTCGGAATTAACCCTCACTAAAGGGAACAAAAGCTGGGTAC

>pIE1-hr5-GFP-HA-ONNnsP3 arg PA

CGGGCCCCCCCTCGAGGGGGATCCGGCGCGTAAAACACAATCAAGTATGAGTCATAAGCTGATGTCATGTTTTGCACACGGCTCATAACCGAACTGGCTTTACGAGTAGAATTCTACTTGTAACGCACGATCAGTGGATGATGGTCATTTGTTTTTCAAATCGAGATGATGTCATGTTTTGCACACGGGCTCATAAACTGCTTTACGAGTAGAATTCTACGTGTAACGCACGATCGAGATGAGTCATTTGTTTTGCAATATGATATCATACAATATGACTCATTTGTTTTTCAAAACCGAACTTGATTTACGGGTAGAATTCTACTCGTAAAGCACAATCAAAAAGATGATGTCATTTGTTTTTCAAAACTGAACTCTCGGCTTTACGAGTAGAATTCTACGTGTAAAACACAATCAAGAAATGATGTCATTTGTTATAAAAATAAAAGCTGATGTCATGTTTTGCACATGGCTCATAACTAAACTCGCTTTACGGGTAGAATTCTACGCGCCGGATCCACTAGCTAGTTCTAGAGTCGCGATGTCTTTGTGATGCGCCGACATTTTTGTAGGTTATTGATAAAATGAACGGATACAGTTGCCCGACATTATCATTAAATCCTTGGCGTAGAATTTGTCGGGTCCATTGTCCGTGTGCGCTAGCATGCCCGCTAACGGACCTCGTACTTTTGGCTTCAAAGGTTTTGCGCACAGACAAAATGTGCCACACTTGCAGCTCTGCATGTGTGCGCGTTACCACAAATCCCAACGGCGCAGTGTACTTGTTGTATGCAAATAAATCTCGATAAAGGCGCGGCGCGCGAATGCAGCTGATCACGTACGCTCCTCGTGTTCCGTTCAAGGACGGTGTTATCGACCTCAGATTAATGTTTATCGGCCGACTGTTTTCGTATCCGCTCACCAAACGCGTTTTTGCATTAACATTGTATGTCGGCGGATGTTCTATATCTAATTTGAATAAATAAACGATAACCGCGTTGGTTTTAGAGGGCATAATAAAAGAAATATTGTTATCGTGTTCGCCATTAGGGCAGTATAAATTGACGTTCATGTTGGATATTGTTTCAGTTGCAAGTTGACACTGGCGGCGACAAGAGATCTGCCGGGCTGCAGGAATTCGATATCAAGCTTATCGATGGGATCCGTGAGCAAGGGCGAGGAGCTGTTCACCGGGGTGGTGCCCATCCTGGTCGAGCTGGACGGCGACGTAAACGGCCACAAGTTCAGCGTGTCCGGCGAGGGCGAGGGCGATGCCACCTACGGCAAGCTGACCCTGAAGTTCATCTGCACCACCGGCAAGCTGCCCGTGCCCTGGCCCACCCTCGTGACCACCCTGACCTACGGCGTGCAGTGCTTCAGCCGCTACCCCGACCACATGAAGCAGCACGACTTCTTCAAGTCCGCCATGCCCGAAGGCTACGTCCAGGAGCGCACCATCTTCTTCAAGGACGACGGCAACTACAAGACCCGCGCCGAGGTGAAGTTCGAGGGCGACACCCTGGTGAACCGCATCGAGCTGAAGGGCATCGACTTCAAGGAGGACGGCAACATCCTGGGGCACAAGCTGGAGTACAACTACAACAGCCACAACGTCTATATCATGGCCGACAAGCAGAAGAACGGCATCAAGGTGAACTTCAAGATCCGCCACAACATCGAGGACGGCAGCGTGCAGCTCGCCGACCACTACCAGCAGAACACCCCCATCGGCGACGGCCCCGTGCTGCTGCCCGACAACCACTACCTGAGCACCCAGTCCGCCCTGAGCAAAGACCCCAACGAGAAGCGCGATCACATGGTCCTGCTGGAGTTCGTGACCGCCGCCGGGATCACTCTCGGCATGGACGAGCTGTACAAGTCCTACCCATACGACGTCCCAGACTACGCTCCGTCATACCGTGTGAAACGGATGGACATCGCGAAAAACACTGAGGAATGCGTGGTAAACGCCGCCAATCCACGCGGAGTACCAGGCGATGGAGTATGTAAAGCCGTGTATAGAAAATGGCCAGAATCATTCAGAAACAGTGCAACGCCAGTGGGTACTGCAAAGACAATCATGTGCGGTCAATACCCCGTCATCCACGCGGTAGGCCCAAACTTCTCAAACTATTCTGAGGCTGAAGGGGATAGGGAATTGGCTTCAGTGTATAGAGAAGTGGCGAAAGAAGTGTCTAGGCTAGGAGTGAGCAGTGTAGCCATCCCTTTGCTTTCAACCGGTGTGTACTCAGGAGGCAAAGATAGACTGCTGCAATCACTAAACCATCTTTTCACAGCGATGGATTCGACAGATGCAGATGTTGTCATCTATTGCAGGGATAAGGAATGGGAGAAGAAGATCACTGAAGCCATATCATTAAGATCCCAGGTAGAACTACTAGATGATCACATCTCAGTGGATTGCGACATTGTACGCGTTCATCCAGACAGCAGCTTGGCAGGCCGAAAGGGGTACAGCACAGTAGAGGGAGCACTCTACTCGTACCTAGAGGGAACAAGATTCCACCAAACTGCTGTAGATATGGCAGAAATATATACCATGTGGCCAAAACAAACTGAAGCCAATGAACAGGTCTGCCTATATGCTCTGGGGGAGAGCATAGAGTCAGTCAGGCAAAAATGTCCTGTAGACGACGCCGACGCCTCATTCCCTCCGAAAACAGTCCCGTGCCTATGCCGTTATGCCATGACGCCTGAACGAGTTGCACGCCTACGCATGAATCATACTACTAGCATCATAGTGTGCTCGTCTTTTCCACTGCCGAAGTACAAAATCGAGGGCGTGCAAAAAGTAAAATGTTCAAAAGCACTCTTGTTTGATCACAACGTACCGTCTCGAGTGAGCCCGAGAACGTACAGGCCTGCGGACGAAATCATACAGACACCTCAAATACCAACTGAAGCGTGCCAGGACGCACAATTCGTGCAGTCAATAACTGATGAAGCAGTGCCAGTTCCGTCAGACTTAGAGGCTTGTGACGCAACTATGGACTGGCCCTCTATCGACATCGTACCAACAAGACAAAGAAGCGACTCATTTGACAGCGAGTATAGTTCCAGAAGTAACATACAGCTGGTGACAGCGGACGTGCATGCACCAATGTACGCAAATTCGCTGGCGTCCAGCGGAGGTTCAGTGCTGTCGCTGTCCAGTGAACAAGCTCAGAACGGCATAATGATACTACCTGATTCAGAAGACACAGATAGTATAAGCAGAGTAAGCACACCGATCGCCCCACCCAGGAGACGTTTGGGAAGGACTATAAATGTGACTTGTGACGAGCGGGAAGGGAAAATACTCCCTATGGCCAGCGACAGGCTCTTCACTGCTAAGCCATACACTGTCGCACTGGGCGTATCAACAGCAGACATAACTGCGTACCCCATCCAGGCACCGCTAGGATCGACACAACCGCCTGCCCTCGAACAGATCACTTTCGGAGATTTCGCCGAAGGTGAAATAGACAACCTCCTGACAGGGGCATTGACATTTGGAGACTTCGAGCCAGGTGAAGTGGAAGAGCTGACGGATAGCGAGTGGTCAACATGCTCGGACACAGATGAAGAGTTACGACTAGACAGAGCAGGGGGTTAGATATTCTCCTCTGACACTGGTCGTCGACCTCGACTAGTGCACTCAACAAAAATGTAATATTAAACACAATTAAATAAATGTTAAAATTTATTGCCTAATATTATTTTGTCATTGCTTGTCATTTATTAATTTGGATGATGTCATTTGTTTTTAAAATTGAACTGGCTTTACGAGTAGAATTCGAGCTCCAATTCGCCCTATAGTGAGTCGTATTACAATTCACTGGCCGTCGTTTTACAACGTCGTGACTGGGAAAACCCTGGCGTTACCCAACTTAATCGCCTTGCAGCACATCCCCCTTTCGCCAGCTGGCGTAATAGCGAAGAGGCCCGCACCGATCGCCCTTCCCAACAGTTGCGCAGCCTGAATGGCGAATGGCGCGACGCGCCCTGTAGCGGCGCATTAAGCGCGGCGGGTGTGGTGGTTACGCGCAGCGTGACCGCTACACTTGCCAGCGCCCTAGCGCCCGCTCCTTTCGCTTTCTTCCCTTCCTTTCTCGCCACGTTCGCCGGCTTTCCCCGTCAAGCTCTAAATCGGGGGCTCCCTTTAGGGTTCCGATTTAGTGCTTTACGGCACCTCGACCCCAAAAAACTTGATTAGGGTGATGGTTCACGTAGTGGGCCATCGCCCTGATAGACGGTTTTTCGCCCTTTGACGTTGGAGTCCACGTTCTTTAATAGTGGACTCTTGTTCCAAACTGGAACAACACTCAACCCTATCTCGGTCTATTCTTTTGATTTATAAGGGATTTTGCCGATTTCGGCCTATTGGTTAAAAAATGAGCTGATTTAACAAAAATTTAACGCGAATTTTAACAAAATATTAACGTTTACAATTTCCCAGGTGGCACTTTTCGGGGAAATGTGCGCGGAACCCCTATTTGTTTATTTTTCTAAATACATTCAAATATGTATCCGCTCATGAGACAATAACCCTGATAAATGCTTCAATAATATTGAAAAAGGAAGAGTATGAGTATTCAACATTTCCGTGTCGCCCTTATTCCCTTTTTTGCGGCATTTTGCCTTCCTGTTTTTGCTCACCCAGAAACGCTGGTGAAAGTAAAAGATGCTGAAGATCAGTTGGGTGCACGAGTGGGTTACATCGAACTGGATCTCAACAGCGGTAAGATCCTTGAGAGTTTTCGCCCCGAAGAACGTTTTCCAATGATGAGCACTTTTAAAGTTCTGCTATGTGGCGCGGTATTATCCCGTATTGACGCCGGGCAAGAGCAACTCGGTCGCCGCATACACTATTCTCAGAATGACTTGGTTGAGTACTCACCAGTCACAGAAAAGCATCTTACGGATGGCATGACAGTAAGAGAATTATGCAGTGCTGCCATAACCATGAGTGATAACACTGCGGCCAACTTACTTCTGACAACGATCGGAGGACCGAAGGAGCTAACCGCTTTTTTGCACAACATGGGGGATCATGTAACTCGCCTTGATCGTTGGGAACCGGAGCTGAATGAAGCCATACCAAACGACGAGCGTGACACCACGATGCCTGTAGCAATGGCAACAACGTTGCGCAAACTATTAACTGGCGAACTACTTACTCTAGCTTCCCGGCAACAATTAATAGACTGGATGGAGGCGGATAAAGTTGCAGGACCACTTCTGCGCTCGGCCCTTCCGGCTGGCTGGTTTATTGCTGATAAATCTGGAGCCGGTGAGCGTGGGTCTCGCGGTATCATTGCAGCACTGGGGCCAGATGGTAAGCCCTCCCGTATCGTAGTTATCTACACGACGGGGAGTCAGGCAACTATGGATGAACGAAATAGACAGATCGCTGAGATAGGTGCCTCACTGATTAAGCATTGGTAACTGTCAGACCAAGTTTACTCATATATACTTTAGATTGATTTAAAACTTCATTTTTAATTTAAAAGGATCTAGGTGAAGATCCTTTTTGATAATCTCATGACCAAAATCCCTTAACGTGAGTTTTCGTTCCACTGAGCGTCAGACCCCGTAGAAAAGATCAAAGGATCTTCTTGAGATCCTTTTTTTCTGCGCGTAATCTGCTGCTTGCAAACAAAAAAACCACCGCTACCAGCGGTGGTTTGTTTGCCGGATCAAGAGCTACCAACTCTTTTTCCGAAGGTAACTGGCTTCAGCAGAGCGCAGATACCAAATACTGTCCTTCTAGTGTAGCCGTAGTTAGGCCACCACTTCAAGAACTCTGTAGCACCGCCTACATACCTCGCTCTGCTAATCCTGTTACCAGTGGCTGCTGCCAGTGGCGATAAGTCGTGTCTTACCGGGTTGGACTCAAGACGATAGTTACCGGATAAGGCGCAGCGGTCGGGCTGAACGGGGGGTTCGTGCACACAGCCCAGCTTGGAGCGAACGACCTACACCGAACTGAGATACCTACAGCGTGAGCTATGAGAAAGCGCCACGCTTCCCGAAGGGAGAAAGGCGGACAGGTATCCGGTAAGCGGCAGGGTCGGAACAGGAGAGCGCACGAGGGAGCTTCCAGGGGGAAACGCCTGGTATCTTTATAGTCCTGTCGGGTTTCGCCACCTCTGACTTGAGCGTCGATTTTTGTGATGCTCGTCAGGGGGGCGGAGCCTATGGAAAAACGCCAGCAACGCGGCCTTTTTACGGTTCCTGGCCTTTTGCTGGCCTTTTGCTCACATGTTCTTTCCTGCGTTATCCCCTGATTCTGTGGATAACCGTATTACCGCCTTTGAGTGAGCTGATACCGCTCGCCGCAGCCGAACGACCGAGCGCAGCGAGTCAGTGAGCGAGGAAGCGGAAGAGCGCCCAATACGCAAACCGCCTCTCCCCGCGCGTTGGCCGATTCATTAATGCAGCTGGCACGACAGGTTTCCCGACTGGAAAGCGGGCAGTGAGCGCAACGCAATTAATGTGAGTTAGCTCACTCATTAGGCACCCCAGGCTTTACACTTTATGCTTCCGGCTCGTATGTTGTGTGGAATTGTGAGCGGATAACAATTTCACACAGGAAACAGCTATGACCATGATTACGCCAAGCTCGGAATTAACCCTCACTAAAGGGAACAAAAGCTGGGTAC

>peGFP-HA

AGCGTGAGCTATGAGAAAGCGCCACGCTTCCCGAAGGGAGAAAGGCGGACAGGTATCCGGTAAGCGGCAGGGTCGGAACAGGAGAGCGCACGAGGGAGCTTCCAGGGGGAAACGCCTGGTATCTTTATAGTCCTGTCGGGTTTCGCCACCTCTGACTTGAGCGTCGATTTTTGTGATGCTCGTCAGGGGGGCGGAGCCTATGGAAAAACGCCAGCAACGCGGCCTTTTTACGGTTCCTGGCCTTTTGCTGGCCTTTTGCTCACATGTTCTTTCCTGCGTTATCCCCTGATTCTGTGGATAACCGTATTACCGCCTTTGAGTGAGCTGATACCGCTCGCCGCAGCCGAACGACCGAGCGCAGCGAGTCAGTGAGCGAGGAAGCGGAAGAGCGCCCAATACGCAAACCGCCTCTCCCCGCGCGTTGGCCGATTCATTAATGCAGCTGGCACGACAGGTTTCCCGACTGGAAAGCGGGCAGTGAGCGCAACGCAATTAATGTGAGTTAGCTCACTCATTAGGCACCCCAGGCTTTACACTTTATGCTTCCGGCTCGTATGTTGTGTGGAATTGTGAGCGGATAACAATTTCACACAGGAAACAGCTATGACCATGATTACGCCAAGCTCGGAATTAACCCTCACTAAAGGGAACAAAAGCTGGGTACCGGGCCCCCCCTCGAGGTCGACGGTATCGATAAGCTTTCTAGAGTCGATGTCTTTGTGATGCGCGCGACATTTTTGTAGGTTATTGATAAAATGAACGGATACGTTGCCCGACATTATCATTAAATCCTTGGCGTAGAATTTGTCGGGTCCATTGTCCGTGTGCGCTAGCATGCCCGTAACGGACCTCGTACTTTTGGCTTCAAAGGTTTTGCGCACAGACAAAATGTGCCACACTTGCAGCTCTGCATGTGTGCGCGTTACCACAAATCCCAACGGCGCAGTGTACTTGTTGTATGCAAATAAATCTCGATAAAGGCGCGGCGCGCGAATGCAGCTGATCACGTACGCTCCTCGTGTTCCGTTCAAGGACGGTGTTATCGACCTCAGATTAATGTTTATCGGCCGACTGTTTTCGTATCCGCTCACCAAACGCGTTTTTGCATTAACATTGTATGTCGGCGGATGTTCTATATCTAATTTGAATAAATAAACGATAACCGCGTTGGTTTTAGAGGGCATAATAAAAGAAATATTGTTATCGTGTTCGCCATTAGGGCAGTATAAATTGACGTTCATGTTGGATATTGTTTCAGTTGCAAGTTGACACTGGCGGCGACAAGATCGTGAACAACCAAGTGACTATGGGATCCGTGAGCAAGGGCGAGGAGCTGTTCACCGGGGTGGTGCCCATCCTGGTCGAGCTGGACGGCGACGTAAACGGCCACAAGTTCAGCGTGTCCGGCGAGGGCGAGGGCGATGCCACCTACGGCAAGCTGACCCTGAAGTTCATCTGCACCACCGGCAAGCTGCCCGTGCCCTGGCCCACCCTCGTGACCACCCTGACCTACGGCGTGCAGTGCTTCAGCCGCTACCCCGACCACATGAAGCAGCACGACTTCTTCAAGTCCGCCATGCCCGAAGGCTACGTCCAGGAGCGCACCATCTTCTTCAAGGACGACGGCAACTACAAGACCCGCGCCGAGGTGAAGTTCGAGGGCGACACCCTGGTGAACCGCATCGAGCTGAAGGGCATCGACTTCAAGGAGGACGGCAACATCCTGGGGCACAAGCTGGAGTACAACTACAACAGCCACAACGTCTATATCATGGCCGACAAGCAGAAGAACGGCATCAAGGTGAACTTCAAGATCCGCCACAACATCGAGGACGGCAGCGTGCAGCTCGCCGACCACTACCAGCAGAACACCCCCATCGGCGACGGCCCCGTGCTGCTGCCCGACAACCACTACCTGAGCACCCAGTCCGCCCTGAGCAAAGACCCCAACGAGAAGCGCGATCACATGGTCCTGCTGGAGTTCGTGACCGCCGCCGGGATCACTCTCGGCATGGACGAGCTGTACAAGTCCTACCCATACGACGTCCCAGACTACGCTGCTAGTCCCGAATTTAATTAATTATACATATATTTTGAATTTAATTAATTATACATATATTTTATATTATTTTTGTCTTTTATTATCGAGGGGCCGTTGTTGGTGTGGGGTTTTGCATAGAAATAACAATGGGAGTTGGCGACGTTGCTGCGCCAACACCACCTCCTCCTCCTCCTTTCATCATGTATCTGTAGATAAAATAAAATATTAAACCTAAAAACAAGACCGCGCCTATCAACAAAATGATAGGCATTAACTTGCCGCTGACGCTGTCACTAACGTTGGACGATTTGCCGACTAAACCTTCATCGCCCAGTAACCAATCTAGAGCGGCCGCCACCGCGGTGGAGCTCCAATTCGCCCTATAGTGAGTCGTATTACAATTCACTGGCCGTCGTTTTACAACGTCGTGACTGGGAAAACCCTGGCGTTACCCAACTTAATCGCCTTGCAGCACATCCCCCTTTCGCCAGCTGGCGTAATAGCGAAGAGGCCCGCACCGATCGCCCTTCCCAACAGTTGCGCAGCCTGAATGGCGAATGGCGCGACGCGCCCTGTAGCGGCGCATTAAGCGCGGCGGGTGTGGTGGTTACGCGCAGCGTGACCGCTACACTTGCCAGCGCCCTAGCGCCCGCTCCTTTCGCTTTCTTCCCTTCCTTTCTCGCCACGTTCGCCGGCTTTCCCCGTCAAGCTCTAAATCGGGGGCTCCCTTTAGGGTTCCGATTTAGTGCTTTACGGCACCTCGACCCCAAAAAACTTGATTAGGGTGATGGTTCACGTAGTGGGCCATCGCCCTGATAGACGGTTTTTCGCCCTTTGACGTTGGAGTCCACGTTCTTTAATAGTGGACTCTTGTTCCAAACTGGAACAACACTCAACCCTATCTCGGTCTATTCTTTTGATTTATAAGGGATTTTGCCGATTTCGGCCTATTGGTTAAAAAATGAGCTGATTTAACAAAAATTTAACGCGAATTTTAACAAAATATTAACGTTTACAATTTCCCAGGTGGCACTTTTCGGGGAAATGTGCGCGGAACCCCTATTTGTTTATTTTTCTAAATACATTCAAATATGTATCCGCTCATGAGACAATAACCCTGATAAATGCTTCAATAATATTGAAAAAGGAAGAGTATGAGTATTCAACATTTCCGTGTCGCCCTTATTCCCTTTTTTGCGGCATTTTGCCTTCCTGTTTTTGCTCACCCAGAAACGCTGGTGAAAGTAAAAGATGCTGAAGATCAGTTGGGTGCACGAGTGGGTTACATCGAACTGGATCTCAACAGCGGTAAGATCCTTGAGAGTTTTCGCCCCGAAGAACGTTTTCCAATGATGAGCACTTTTAAAGTTCTGCTATGTGGCGCGGTATTATCCCGTATTGACGCCGGGCAAGAGCAACTCGGTCGCCGCATACACTATTCTCAGAATGACTTGGTTGAGTACTCACCAGTCACAGAAAAGCATCTTACGGATGGCATGACAGTAAGAGAATTATGCAGTGCTGCCATAACCATGAGTGATAACACTGCGGCCAACTTACTTCTGACAACGATCGGAGGACCGAAGGAGCTAACCGCTTTTTTGCACAACATGGGGGATCATGTAACTCGCCTTGATCGTTGGGAACCGGAGCTGAATGAAGCCATACCAAACGACGAGCGTGACACCACGATGCCTGTAGCAATGGCAACAACGTTGCGCAAACTATTAACTGGCGAACTACTTACTCTAGCTTCCCGGCAACAATTAATAGACTGGATGGAGGCGGATAAAGTTGCAGGACCACTTCTGCGCTCGGCCCTTCCGGCTGGCTGGTTTATTGCTGATAAATCTGGAGCCGGTGAGCGTGGGTCTCGCGGTATCATTGCAGCACTGGGGCCAGATGGTAAGCCCTCCCGTATCGTAGTTATCTACACGACGGGGAGTCAGGCAACTATGGATGAACGAAATAGACAGATCGCTGAGATAGGTGCCTCACTGATTAAGCATTGGTAACTGTCAGACCAAGTTTACTCATATATACTTTAGATTGATTTAAAACTTCATTTTTAATTTAAAAGGATCTAGGTGAAGATCCTTTTTGATAATCTCATGACCAAAATCCCTTAACGTGAGTTTTCGTTCCACTGAGCGTCAGACCCCGTAGAAAAGATCAAAGGATCTTCTTGAGATCCTTTTTTTCTGCGCGTAATCTGCTGCTTGCAAACAAAAAAACCACCGCTACCAGCGGTGGTTTGTTTGCCGGATCAAGAGCTACCAACTCTTTTTCCGAAGGTAACTGGCTTCAGCAGAGCGCAGATACCAAATACTGTCCTTCTAGTGTAGCCGTAGTTAGGCCACCACTTCAAGAACTCTGTAGCACCGCCTACATACCTCGCTCTGCTAATCCTGTTACCAGTGGCTGCTGCCAGTGGCGATAAGTCGTGTCTTACCGGGTTGGACTCAAGACGATAGTTACCGGATAAGGCGCAGCGGTCGGGCTGAACGGGGGGTTCGTGCACACAGCCCAGCTTGGAGCGAACGACCTACACCGAACTGAGATACCTAC

>pIE1-hr5-GFP

CGGGCCCCCCCTCGAGGGGGATCCGGCGCGTAAAACACAATCAAGTATGAGTCATAAGCTGATGTCATGTTTTGCACACGGCTCATAACCGAACTGGCTTTACGAGTAGAATTCTACTTGTAACGCACGATCAGTGGATGATGGTCATTTGTTTTTCAAATCGAGATGATGTCATGTTTTGCACACGGGCTCATAAACTGCTTTACGAGTAGAATTCTACGTGTAACGCACGATCGAGATGAGTCATTTGTTTTGCAATATGATATCATACAATATGACTCATTTGTTTTTCAAAACCGAACTTGATTTACGGGTAGAATTCTACTCGTAAAGCACAATCAAAAAGATGATGTCATTTGTTTTTCAAAACTGAACTCTCGGCTTTACGAGTAGAATTCTACGTGTAAAACACAATCAAGAAATGATGTCATTTGTTATAAAAATAAAAGCTGATGTCATGTTTTGCACATGGCTCATAACTAAACTCGCTTTACGGGTAGAATTCTACGCGCCGGATCCACTAGCTAGTTCTAGAGTCGCGATGTCTTTGTGATGCGCCGACATTTTTGTAGGTTATTGATAAAATGAACGGATACAGTTGCCCGACATTATCATTAAATCCTTGGCGTAGAATTTGTCGGGTCCATTGTCCGTGTGCGCTAGCATGCCCGCTAACGGACCTCGTACTTTTGGCTTCAAAGGTTTTGCGCACAGACAAAATGTGCCACACTTGCAGCTCTGCATGTGTGCGCGTTACCACAAATCCCAACGGCGCAGTGTACTTGTTGTATGCAAATAAATCTCGATAAAGGCGCGGCGCGCGAATGCAGCTGATCACGTACGCTCCTCGTGTTCCGTTCAAGGACGGTGTTATCGACCTCAGATTAATGTTTATCGGCCGACTGTTTTCGTATCCGCTCACCAAACGCGTTTTTGCATTAACATTGTATGTCGGCGGATGTTCTATATCTAATTTGAATAAATAAACGATAACCGCGTTGGTTTTAGAGGGCATAATAAAAGAAATATTGTTATCGTGTTCGCCATTAGGGCAGTATAAATTGACGTTCATGTTGGATATTGTTTCAGTTGCAAGTTGACACTGGCGGCGACAAGAGATCTGCCGGGCTGCAGGAATTCGATATCAAGCTTATCGATACCGTCGACCTCGACTAGCGCTACCGGTCGCCACCATGGTGAGCAAGGGCGAGGAGCTGTTCACCGGGGTGGTGCCCATCCTGGTCGAGCTGGACGGCGACGTAAACGGCCACAAGTTCAGCGTGTCCGGCGAGGGCGAGGGCGATGCCACCTACGGCAAGCTGACCCTGAAGTTCATCTGCACCACCGGCAAGCTGCCCGTGCCCTGGCCCACCCTCGTGACCACCCTGACCTACGGCGTGCAGTGCTTCAGCCGCTACCCCGACCACATGAAGCAGCACGACTTCTTCAAGTCCGCCATGCCCGAAGGCTACGTCCAGGAGCGCACCATCTTCTTCAAGGACGACGGCAACTACAAGACCCGCGCCGAGGTGAAGTTCGAGGGCGACACCCTGGTGAACCGCATCGAGCTGAAGGGCATCGACTTCAAGGAGGACGGCAACATCCTGGGGCACAAGCTGGAGTACAACTACAACAGCCACAACGTCTATATCATGGCCGACAAGCAGAAGAACGGCATCAAGGTGAACTTCAAGATCCGCCACAACATCGAGGACGGCAGCGTGCAGCTCGCCGACCACTACCAGCAGAACACCCCCATCGGCGACGGCCCCGTGCTGCTGCCCGACAACCACTACCTGAGCACCCAGTCCGCCCTGAGCAAAGACCCCAACGAGAAGCGCGATCACATGGTCCTGCTGGAGTTCGTGACCGCCGCCGGGATCACTCTCGGCATGGACGAGCTGTACAAGTCCGGCCGGACTCAGATCTCGAGCTCAAGCTTCGAATTCTGCAGTCGACGGTACCGCGGGCCCTGCACTCAACAAAAATGTAATATTAAACACAATTAAATAAATGTTAAAATTTATTGCCTAATATTATTTTGTCATTGCTTGTCATTTATTAATTTGGATGATGTCATTTGTTTTTAAAATTGAACTGGCTTTACGAGTAGAATTCGAGCTCCAATTCGCCCTATAGTGAGTCGTATTACAATTCACTGGCCGTCGTTTTACAACGTCGTGACTGGGAAAACCCTGGCGTTACCCAACTTAATCGCCTTGCAGCACATCCCCCTTTCGCCAGCTGGCGTAATAGCGAAGAGGCCCGCACCGATCGCCCTTCCCAACAGTTGCGCAGCCTGAATGGCGAATGGCGCGACGCGCCCTGTAGCGGCGCATTAAGCGCGGCGGGTGTGGTGGTTACGCGCAGCGTGACCGCTACACTTGCCAGCGCCCTAGCGCCCGCTCCTTTCGCTTTCTTCCCTTCCTTTCTCGCCACGTTCGCCGGCTTTCCCCGTCAAGCTCTAAATCGGGGGCTCCCTTTAGGGTTCCGATTTAGTGCTTTACGGCACCTCGACCCCAAAAAACTTGATTAGGGTGATGGTTCACGTAGTGGGCCATCGCCCTGATAGACGGTTTTTCGCCCTTTGACGTTGGAGTCCACGTTCTTTAATAGTGGACTCTTGTTCCAAACTGGAACAACACTCAACCCTATCTCGGTCTATTCTTTTGATTTATAAGGGATTTTGCCGATTTCGGCCTATTGGTTAAAAAATGAGCTGATTTAACAAAAATTTAACGCGAATTTTAACAAAATATTAACGTTTACAATTTCCCAGGTGGCACTTTTCGGGGAAATGTGCGCGGAACCCCTATTTGTTTATTTTTCTAAATACATTCAAATATGTATCCGCTCATGAGACAATAACCCTGATAAATGCTTCAATAATATTGAAAAAGGAAGAGTATGAGTATTCAACATTTCCGTGTCGCCCTTATTCCCTTTTTTGCGGCATTTTGCCTTCCTGTTTTTGCTCACCCAGAAACGCTGGTGAAAGTAAAAGATGCTGAAGATCAGTTGGGTGCACGAGTGGGTTACATCGAACTGGATCTCAACAGCGGTAAGATCCTTGAGAGTTTTCGCCCCGAAGAACGTTTTCCAATGATGAGCACTTTTAAAGTTCTGCTATGTGGCGCGGTATTATCCCGTATTGACGCCGGGCAAGAGCAACTCGGTCGCCGCATACACTATTCTCAGAATGACTTGGTTGAGTACTCACCAGTCACAGAAAAGCATCTTACGGATGGCATGACAGTAAGAGAATTATGCAGTGCTGCCATAACCATGAGTGATAACACTGCGGCCAACTTACTTCTGACAACGATCGGAGGACCGAAGGAGCTAACCGCTTTTTTGCACAACATGGGGGATCATGTAACTCGCCTTGATCGTTGGGAACCGGAGCTGAATGAAGCCATACCAAACGACGAGCGTGACACCACGATGCCTGTAGCAATGGCAACAACGTTGCGCAAACTATTAACTGGCGAACTACTTACTCTAGCTTCCCGGCAACAATTAATAGACTGGATGGAGGCGGATAAAGTTGCAGGACCACTTCTGCGCTCGGCCCTTCCGGCTGGCTGGTTTATTGCTGATAAATCTGGAGCCGGTGAGCGTGGGTCTCGCGGTATCATTGCAGCACTGGGGCCAGATGGTAAGCCCTCCCGTATCGTAGTTATCTACACGACGGGGAGTCAGGCAACTATGGATGAACGAAATAGACAGATCGCTGAGATAGGTGCCTCACTGATTAAGCATTGGTAACTGTCAGACCAAGTTTACTCATATATACTTTAGATTGATTTAAAACTTCATTTTTAATTTAAAAGGATCTAGGTGAAGATCCTTTTTGATAATCTCATGACCAAAATCCCTTAACGTGAGTTTTCGTTCCACTGAGCGTCAGACCCCGTAGAAAAGATCAAAGGATCTTCTTGAGATCCTTTTTTTCTGCGCGTAATCTGCTGCTTGCAAACAAAAAAACCACCGCTACCAGCGGTGGTTTGTTTGCCGGATCAAGAGCTACCAACTCTTTTTCCGAAGGTAACTGGCTTCAGCAGAGCGCAGATACCAAATACTGTCCTTCTAGTGTAGCCGTAGTTAGGCCACCACTTCAAGAACTCTGTAGCACCGCCTACATACCTCGCTCTGCTAATCCTGTTACCAGTGGCTGCTGCCAGTGGCGATAAGTCGTGTCTTACCGGGTTGGACTCAAGACGATAGTTACCGGATAAGGCGCAGCGGTCGGGCTGAACGGGGGGTTCGTGCACACAGCCCAGCTTGGAGCGAACGACCTACACCGAACTGAGATACCTACAGCGTGAGCTATGAGAAAGCGCCACGCTTCCCGAAGGGAGAAAGGCGGACAGGTATCCGGTAAGCGGCAGGGTCGGAACAGGAGAGCGCACGAGGGAGCTTCCAGGGGGAAACGCCTGGTATCTTTATAGTCCTGTCGGGTTTCGCCACCTCTGACTTGAGCGTCGATTTTTGTGATGCTCGTCAGGGGGGCGGAGCCTATGGAAAAACGCCAGCAACGCGGCCTTTTTACGGTTCCTGGCCTTTTGCTGGCCTTTTGCTCACATGTTCTTTCCTGCGTTATCCCCTGATTCTGTGGATAACCGTATTACCGCCTTTGAGTGAGCTGATACCGCTCGCCGCAGCCGAACGACCGAGCGCAGCGAGTCAGTGAGCGAGGAAGCGGAAGAGCGCCCAATACGCAAACCGCCTCTCCCCGCGCGTTGGCCGATTCATTAATGCAGCTGGCACGACAGGTTTCCCGACTGGAAAGCGGGCAGTGAGCGCAACGCAATTAATGTGAGTTAGCTCACTCATTAGGCACCCCAGGCTTTACACTTTATGCTTCCGGCTCGTATGTTGTGTGGAATTGTGAGCGGATAACAATTTCACACAGGAAACAGCTATGACCATGATTACGCCAAGCTCGGAATTAACCCTCACTAAAGGGAACAAAAGCTGGGTAC

>pIE1prm/hr5/PA

CGGGCCCCCCCTCGAGGGGGATCCGGCGCGTAAAACACAATCAAGTATGAGTCATAAGCTGATGTCATGTTTTGCACACGGCTCATAACCGAACTGGCTTTACGAGTAGAATTCTACTTGTAACGCACGATCAGTGGATGATGGTCATTTGTTTTTCAAATCGAGATGATGTCATGTTTTGCACACGGGCTCATAAACTGCTTTACGAGTAGAATTCTACGTGTAACGCACGATCGAGATGAGTCATTTGTTTTGCAATATGATATCATACAATATGACTCATTTGTTTTTCAAAACCGAACTTGATTTACGGGTAGAATTCTACTCGTAAAGCACAATCAAAAAGATGATGTCATTTGTTTTTCAAAACTGAACTCTCGGCTTTACGAGTAGAATTCTACGTGTAAAACACAATCAAGAAATGATGTCATTTGTTATAAAAATAAAAGCTGATGTCATGTTTTGCACATGGCTCATAACTAAACTCGCTTTACGGGTAGAATTCTACGCGCCGGATCCACTAGCTAGTTCTAGAGTCGCGATGTCTTTGTGATGCGCCGACATTTTTGTAGGTTATTGATAAAATGAACGGATACAGTTGCCCGACATTATCATTAAATCCTTGGCGTAGAATTTGTCGGGTCCATTGTCCGTGTGCGCTAGCATGCCCGCTAACGGACCTCGTACTTTTGGCTTCAAAGGTTTTGCGCACAGACAAAATGTGCCACACTTGCAGCTCTGCATGTGTGCGCGTTACCACAAATCCCAACGGCGCAGTGTACTTGTTGTATGCAAATAAATCTCGATAAAGGCGCGGCGCGCGAATGCAGCTGATCACGTACGCTCCTCGTGTTCCGTTCAAGGACGGTGTTATCGACCTCAGATTAATGTTTATCGGCCGACTGTTTTCGTATCCGCTCACCAAACGCGTTTTTGCATTAACATTGTATGTCGGCGGATGTTCTATATCTAATTTGAATAAATAAACGATAACCGCGTTGGTTTTAGAGGGCATAATAAAAGAAATATTGTTATCGTGTTCGCCATTAGGGCAGTATAAATTGACGTTCATGTTGGATATTGTTTCAGTTGCAAGTTGACACTGGCGGCGACAAGAGATCTGCCGGGCTGCAGGAATTCGATATCAAGCTTATCGATACCGTCGACCTCGACTAGTGCACTCAACAAAAATGTAATATTAAACACAATTAAATAAATGTTAAAATTTATTGCCTAATATTATTTTGTCATTGCTTGTCATTTATTAATTTGGATGATGTCATTTGTTTTTAAAATTGAACTGGCTTTACGAGTAGAATTCGAGCTCCAATTCGCCCTATAGTGAGTCGTATTACAATTCACTGGCCGTCGTTTTACAACGTCGTGACTGGGAAAACCCTGGCGTTACCCAACTTAATCGCCTTGCAGCACATCCCCCTTTCGCCAGCTGGCGTAATAGCGAAGAGGCCCGCACCGATCGCCCTTCCCAACAGTTGCGCAGCCTGAATGGCGAATGGCGCGACGCGCCCTGTAGCGGCGCATTAAGCGCGGCGGGTGTGGTGGTTACGCGCAGCGTGACCGCTACACTTGCCAGCGCCCTAGCGCCCGCTCCTTTCGCTTTCTTCCCTTCCTTTCTCGCCACGTTCGCCGGCTTTCCCCGTCAAGCTCTAAATCGGGGGCTCCCTTTAGGGTTCCGATTTAGTGCTTTACGGCACCTCGACCCCAAAAAACTTGATTAGGGTGATGGTTCACGTAGTGGGCCATCGCCCTGATAGACGGTTTTTCGCCCTTTGACGTTGGAGTCCACGTTCTTTAATAGTGGACTCTTGTTCCAAACTGGAACAACACTCAACCCTATCTCGGTCTATTCTTTTGATTTATAAGGGATTTTGCCGATTTCGGCCTATTGGTTAAAAAATGAGCTGATTTAACAAAAATTTAACGCGAATTTTAACAAAATATTAACGTTTACAATTTCCCAGGTGGCACTTTTCGGGGAAATGTGCGCGGAACCCCTATTTGTTTATTTTTCTAAATACATTCAAATATGTATCCGCTCATGAGACAATAACCCTGATAAATGCTTCAATAATATTGAAAAAGGAAGAGTATGAGTATTCAACATTTCCGTGTCGCCCTTATTCCCTTTTTTGCGGCATTTTGCCTTCCTGTTTTTGCTCACCCAGAAACGCTGGTGAAAGTAAAAGATGCTGAAGATCAGTTGGGTGCACGAGTGGGTTACATCGAACTGGATCTCAACAGCGGTAAGATCCTTGAGAGTTTTCGCCCCGAAGAACGTTTTCCAATGATGAGCACTTTTAAAGTTCTGCTATGTGGCGCGGTATTATCCCGTATTGACGCCGGGCAAGAGCAACTCGGTCGCCGCATACACTATTCTCAGAATGACTTGGTTGAGTACTCACCAGTCACAGAAAAGCATCTTACGGATGGCATGACAGTAAGAGAATTATGCAGTGCTGCCATAACCATGAGTGATAACACTGCGGCCAACTTACTTCTGACAACGATCGGAGGACCGAAGGAGCTAACCGCTTTTTTGCACAACATGGGGGATCATGTAACTCGCCTTGATCGTTGGGAACCGGAGCTGAATGAAGCCATACCAAACGACGAGCGTGACACCACGATGCCTGTAGCAATGGCAACAACGTTGCGCAAACTATTAACTGGCGAACTACTTACTCTAGCTTCCCGGCAACAATTAATAGACTGGATGGAGGCGGATAAAGTTGCAGGACCACTTCTGCGCTCGGCCCTTCCGGCTGGCTGGTTTATTGCTGATAAATCTGGAGCCGGTGAGCGTGGGTCTCGCGGTATCATTGCAGCACTGGGGCCAGATGGTAAGCCCTCCCGTATCGTAGTTATCTACACGACGGGGAGTCAGGCAACTATGGATGAACGAAATAGACAGATCGCTGAGATAGGTGCCTCACTGATTAAGCATTGGTAACTGTCAGACCAAGTTTACTCATATATACTTTAGATTGATTTAAAACTTCATTTTTAATTTAAAAGGATCTAGGTGAAGATCCTTTTTGATAATCTCATGACCAAAATCCCTTAACGTGAGTTTTCGTTCCACTGAGCGTCAGACCCCGTAGAAAAGATCAAAGGATCTTCTTGAGATCCTTTTTTTCTGCGCGTAATCTGCTGCTTGCAAACAAAAAAACCACCGCTACCAGCGGTGGTTTGTTTGCCGGATCAAGAGCTACCAACTCTTTTTCCGAAGGTAACTGGCTTCAGCAGAGCGCAGATACCAAATACTGTCCTTCTAGTGTAGCCGTAGTTAGGCCACCACTTCAAGAACTCTGTAGCACCGCCTACATACCTCGCTCTGCTAATCCTGTTACCAGTGGCTGCTGCCAGTGGCGATAAGTCGTGTCTTACCGGGTTGGACTCAAGACGATAGTTACCGGATAAGGCGCAGCGGTCGGGCTGAACGGGGGGTTCGTGCACACAGCCCAGCTTGGAGCGAACGACCTACACCGAACTGAGATACCTACAGCGTGAGCTATGAGAAAGCGCCACGCTTCCCGAAGGGAGAAAGGCGGACAGGTATCCGGTAAGCGGCAGGGTCGGAACAGGAGAGCGCACGAGGGAGCTTCCAGGGGGAAACGCCTGGTATCTTTATAGTCCTGTCGGGTTTCGCCACCTCTGACTTGAGCGTCGATTTTTGTGATGCTCGTCAGGGGGGCGGAGCCTATGGAAAAACGCCAGCAACGCGGCCTTTTTACGGTTCCTGGCCTTTTGCTGGCCTTTTGCTCACATGTTCTTTCCTGCGTTATCCCCTGATTCTGTGGATAACCGTATTACCGCCTTTGAGTGAGCTGATACCGCTCGCCGCAGCCGAACGACCGAGCGCAGCGAGTCAGTGAGCGAGGAAGCGGAAGAGCGCCCAATACGCAAACCGCCTCTCCCCGCGCGTTGGCCGATTCATTAATGCAGCTGGCACGACAGGTTTCCCGACTGGAAAGCGGGCAGTGAGCGCAACGCAATTAATGTGAGTTAGCTCACTCATTAGGCACCCCAGGCTTTACACTTTATGCTTCCGGCTCGTATGTTGTGTGGAATTGTGAGCGGATAACAATTTCACACAGGAAACAGCTATGACCATGATTACGCCAAGCTCGGAATTAACCCTCACTAAAGGGAACAAAAGCTGGGTAC

>pCHIKV/ONNVnsP3

CTAAATTGTAAGCGTTAATATTTTGTTAAAATTCGCGTTAAATTTTTGTTAAATCAGCTCATTTTTTAACCAATAGGCCGAAATCGGCAAAATCCCTTATAAATCAAAAGAATAGACCGAGATAGGGTTGAGTGTTGTTCCAGTTTGGAACAAGAGTCCACTATTAAAGAACGTGGACTCCAACGTCAAAGGGCGAAAAACCGTCTATCAGGGCGATGGCCCACTACGTGAACCATCACCCTAATCAAGTTTTTTGGGGTCGAGGTGCCGTAAAGCACTAAATCGGAACCCTAAAGGGAGCCCCCGATTTAGAGCTTGACGGGGAAAGCCGGCGAACGTGGCGAGAAAGGAAGGGAAGAAAGCGAAAGGAGCGGGCGCTAGGGCGCTGGCAAGTGTAGCGGTCACGCTGCGCGTAACCACCACACCCGCCGCGCTTAATGCGCCGCTACAGGGCGCGTCCCATTCGCCATTCAGGCTGCGCAACTGTTGGGAAGGGCGATCGGTGCGGGCCTCTTCGCTATTACGCCAGCTGGCGAAAGGGGGATGTGCTGCAAGGCGATTAAGTTGGGTAACGCCAGGGTTTTCCCAGTCACGACGTTGTAAAACGACGGCCAGTGAGCGCGCGTAATACGACTCACTATAGGGCGAATTGGGTACCGGGCCCCCCCTCGAGTAATACGACTCACTATAATGGCTGCGTGAGACACACGTAGCCTACCAGTTTCTTACTGCTCTACTCTGCTTAGCAAGAGACTTGAGAACCCATCATGGATCCCGTGTACGTGGACATAGACGCCGACAGCGCCTTTTTAAAGGCCCTGCAGCGTGCGTACCCCATGTTTGAGGTGGAACCAAGGCAGGTCACACCGAATGACCATGCCAATGCTAGAGCATTCTCGCATCTAGCTATAAAACTAATAGAGCAGGAAATTGATCCCGACTCAACCATCCTGGACATAGGCAGCGCGCCAGCAAGGAGGATGATGTCGGATAGGAAGTACCACTGCGTTTGCCCTATGCGCAGCGCAGAAGACCCTGAGAGACTCGCCAACTACGCGAGAAAACTAGCATCTGCCGCAGGAAAAGTCTTGGACAGAAACATCTCCGAAAAAATTGGAGATCTACAAGCAGTAATGGCTGTACCAGACGCAGAAACGCCCACATTCTGCTTGCACACTGACGTCTCATGTAGACAAAGGGCGGACGTCGCTATATACCAGGATGTCTACGCCGTGCATGCACCAACATCGCTGTACCACCAGGCGATTAAAGGAGTCCGTGTAGCATACTGGATAGGGTTTGATACAACCCCGTTCATGTATAATGCCATGGCAGGTGCATACCCCTCGTACTCGACAAACTGGGCAGATGAGCAGGTGCTGAAGGCAAAGAACATAGGATTATGTTCAACAGACCTGACGGAAGGTAGACGAGGTAAATTGTCTATCATGAGAGGAAAAAAGATGAAGCCATGTGACCGCGTACTGTTCTCAGTCGGGTCAACGCTTTACCCGGAGAGCCGTAAGCTTCTTAAGAGTTGGCACTTACCTTCAGTGTTCCATCTAAAAGGGAAGCTCAGCTTCACGTGCCGCTGTGATACAGTGGTTTCGTGTGAAGGCTATGTCGTTAAGAGAATAACGATTAGCCCGGGCCTCTACGGTAAAACCACAGGGTACGCAGTAACCCACCATGCAGACGGATTCCTAATGTGCAAAACAACCGATACGGTAGATGGCGAGAGAGTGTCATTTTCGGTATGCACGTACGTACCCGCAACCATTTGTGATCAAATGACAGGTATTCTTGCCACGGAGGTTACACCGGAGGATGCACAGAAGCTGCTGGTGGGACTGAACCAGAGGATAGTGGTCAATGGCAGAACGCAGAGGAACACGAACACAATGAAGAATTACTTGCTTCCTGTAGTTGCCCAAGCCTTCAGTAAGTGGGCAAAGGAATGCCGGAAAGATATGGAAGATGAAAAACTTTTGGGCATCAGAGAAAGGACACTGACATGCTGCTGCCTTTGGGCGTTCAAGAAGCAGAAGACACACACGGTCTACAAGAGGCCTGACACTCAGTCAATTCAGAAAGTCCCAGCCGAATTTGACAGCTTTGTGGTACCAAGTCTGTGGTCATCTGGACTGTCGATCCCGCTACGGACCAGAATCAAGTGGCTGCTAAGCAAAGTGCCAAAGACTGATTTGATCCCTTACAGCGGTGACGCCAAAGAAGCCCGCGACGCTGAAAAAGAAGCAGAAGAAGAACGAGAAGCGGAGCTAACTCGCGAGGCACTACCACCACTACAGGCGGCACAGGACGACGTCCAGGTCGAAATTGACGTGGAACAGCTCGAAGACAGAGCTGGGGCAGGAATAATTGAAACTCCAAGAGGAGCTATCAAAGTCACTGCCCAACCAACAGACCACGTCGTGGGAGAGTACTTGGTACTTTCCCCGCAGACCGTGTTACGAAGCCAGAAGCTCAGCCTGATCCACGCATTGGCGGAACAAGTGAAGACATGCACACACAGCGGACGGGCAGGAAGGTACGCGGTCGAAGCATATGACGGCAGAATCCTTGTGCCCTCAGGCTATGCAATATCACCTGAAGACTTCCAGAGCCTGAGCGAAAGTGCGACGATGGTGTACAACGAAAGGGAGTTCGTAAATAGGAAATTACACCATATCGCGTTGCACGGACCAGCCCTGAACACTGACGAGGAGTCGTACGAGCTGGTAAGGGCAGAAAGGACAGAGCATGAGTACGTCTATGATGTGGACCAAAGAAGGTGCTGCAAGAAAGAGGAGGCAGCCGGGCTGGTACTGGTCGGCGACTTGACCAACCCGCCCTACCATGAGTTCGCATATGAAGGGCTGAGAATCCGCCCCGCCTGCCCATACAAGACCGCAGTAATAGGGGTCTTTGGAGTGCCAGGATCCGGCAAATCAGCAATCATTAAGAACCTAGTTACCAGGCAAGACCTAGTGACCAGTGGAAAGAAAGAAAACTGCCAAGAAATCTCCACCGACGTGATGCGACAGAGGAACCTGGAGATATCTGCACGCACGGTCGACTCACTGCTCTTGAACGGATGCAATAGACCAGTCGACGTGTTGTACGTCGACGAAGCTTTTGCGTGCCATTCTGGCACGCTACTTGCTCTGATAGCCTTGGTGAGACCGAGGCAGAAAGTCGTGCTATGCGGTGATCCGAAACAGTGCGGCTTCTTCAATATGATGCAGATGAAAGTTAACTACAACCATAACATCTGCACCCAAGTGTACCATAAAAGTATTTCCAGGCGGTGTACACTGCCTGTGACTGCCATTGTGTCCTCGTTGCATTACGAAGGCAAAATGCGCACAACAAATGAGTACAACAAGCCAATTGTAGTGGATACTACAGGCTCGACAAAACCCGACCCCGGAGACCTTGTGCTAACATGTTTCAGAGGGTGGGTTAAGCAACTGCAAATTGACTATCGTGGACACGAGGTCATGACAGCAGCTGCATCTCAGGGGCTAACCAGAAAAGGGGTCTATGCCGTCAGGCAAAAAGTTAATGAAAACCCCCTTTACGCATCAACATCAGAGCACGTGAACGTGCTACTGACGCGTACGGAAGGCAAACTAGTATGGAAGACACTTTCTGGAGACCCATGGATAAAGACACTGCAGAACCCGCCGAAAGGAAATTTTAAAGCAACAATTAAGGAATGGGAAGTGGAACATGCTTCAATAATGGCGGGTATCTGTAACCACCAAGTGACCTTTGACACGTTCCAGAATAAAGCCAATGTCTGCTGGGCGAAGAGCTTAGTCCCCATCCTAGAAACAGCAGGGATAAAATTAAACGACAGGCAGTGGTCCCAGATAATCCAGGCTTTTAAAGAAGACAGAGCATACTCACCCGAGGTGGCCCTGAATGAGATATGCACGCGCATGTACGGGGTAGACCTGGACAGCGGACTGTTCTCTAAACCACTGGTGTCCGTGCATTATGCGGATAATCACTGGGACAACAGGCCGGGAGGGAAGATGTTCGGATTCAACCCCGAAGCGGCGTCCATACTGGAGAGGAAATACCCGTTTACAAAAGGGAAGTGGAATACCAACAAGCAAATCTGTGTGACTACTAGGAGGATTGAAGATTTTAACCCGAACACCAACATTATACCTGCCAACAGGAGATTACCGCATTCATTGGTGGCCGAACATCGCCCGGTAAAAGGGGAGAGGATGGAATGGTTGGTCAACAAAATAAATGGCCACCATGTGCTCCTGGTCAGCGGCTACAACCTCGTTCTGCCCACTAAGAGAGTCACCTGGGTGGCGCCGCTGGGCATTCGGGGAGCTGACTACACATACAACCTAGAGTTAGGCCTACCAGCAACGCTCGGTAGATATGACCTAGTGATTATAAACATCCACACACCCTTTCGCATACATCATTACCAACAGTGCGTGGATCACGCAATGAAGCTGCAGATGCTCGGAGGAGACTCCCTGAGACTGCTCAAGCCGGGTGGTTCATTACTGATCAGGGCATACGGCTACGCAGACAGAACAAGCGAACGAGTAGTCTGCGTATTGGGACGCAAGTTTCGATCATCCAGAGCGTTGAAACCGCCGTGCGTCACTAGCAACACCGAGATGTTTTTCTTGTTCAGCAACTTTGATAACGGCAGAAGGAACTTTACGACGCACGTAATGAACAACCAGCTGAATGCTGCTTTTGTTGGTCAGGCCACCCGAGCAGGGTGCGCTCCGTCATACCGTGTGAAACGGATGGACATCGCGAAAAACACTGAGGAATGCGTGGTAAACGCCGCCAATCCACGCGGAGTACCAGGCGATGGAGTATGTAAAGCCGTGTATAGAAAATGGCCAGAATCATTCAGAAACAGTGCAACGCCAGTGGGTACTGCAAAGACAATCATGTGCGGTCAATACCCCGTCATCCACGCGGTAGGCCCAAACTTCTCAAACTATTCTGAGGCTGAAGGGGATAGGGAATTGGCTTCAGTGTATAGAGAAGTGGCGAAAGAAGTGTCTAGGCTAGGAGTGAGCAGTGTAGCCATCCCTTTGCTTTCAACCGGTGTGTACTCAGGAGGCAAAGATAGACTGCTGCAATCACTAAACCATCTTTTCACAGCGATGGATTCGACAGATGCAGATGTTGTCATCTATTGCAGGGATAAGGAATGGGAGAAGAAGATCACTGAAGCCATATCATTAAGATCCCAGGTAGAACTACTAGATGATCACATCTCAGTGGATTGCGACATTGTACGCGTTCATCCAGACAGCAGCTTGGCAGGCCGAAAGGGGTACAGCACAGTAGAGGGAGCACTCTACTCGTACCTAGAGGGAACAAGATTCCACCAAACTGCTGTAGATATGGCAGAAATATATACCATGTGGCCAAAACAAACTGAAGCCAATGAACAGGTCTGCCTATATGCTCTGGGGGAGAGCATAGAGTCAGTCAGGCAAAAATGTCCTGTAGACGACGCCGACGCCTCATTCCCTCCGAAAACAGTCCCGTGCCTATGCCGTTATGCCATGACGCCTGAACGAGTTGCACGCCTACGCATGAATCATACTACTAGCATCATAGTGTGCTCGTCTTTTCCACTGCCGAAGTACAAAATCGAGGGCGTGCAAAAAGTAAAATGTTCAAAAGCACTCTTGTTTGATCACAACGTACCGTCTCGAGTGAGCCCGAGAACGTACAGGCCTGCGGACGAAATCATACAGACACCTCAAATACCAACTGAAGCGTGCCAGGACGCACAATTCGTGCAGTCAATAACTGATGAAGCAGTGCCAGTTCCGTCAGACTTAGAGGCTTGTGACGCAACTATGGACTGGCCCTCTATCGACATCGTACCAACAAGACAAAGAAGCGACTCATTTGACAGCGAGTATAGTTCCAGAAGTAACATACAGCTGGTGACAGCGGACGTGCATGCACCAATGTACGCAAATTCGCTGGCGTCCAGCGGAGGTTCAGTGCTGTCGCTGTCCAGTGAACAAGCTCAGAACGGCATAATGATACTACCTGATTCAGAAGACACAGATAGTATAAGCAGAGTAAGCACACCGATCGCCCCACCCAGGAGACGTTTGGGAAGGACTATAAATGTGACTTGTGACGAGCGGGAAGGGAAAATACTCCCTATGGCCAGCGACAGGCTCTTCACTGCTAAGCCATACACTGTCGCACTGGGCGTATCAACAGCAGACATAACTGCGTACCCCATCCAGGCACCGCTAGGATCGACACAACCGCCTGCCCTCGAACAGATCACTTTCGGAGATTTCGCCGAAGGTGAAATAGACAACCTCCTGACAGGGGCATTGACATTTGGAGACTTCGAGCCAGGTGAAGTGGAAGAGCTGACGGATAGCGAGTGGTCAACATGCTCGGACACAGATGAAGAGTTACGACTAGATAGGGCAGGTGGGTACATATTCTCATCTGACACCGGCCCCGGCCACCTGCAACAGAGGTCTGTCCGTCAGACAGTACTGCCGGTAAATACCTTGGAGGAAGTTCAGGAGGAGAAATGTTACCCACCTAAGTTGGATGAAGTGAAAGAGCAGTTGTTACTTAAGAAACTCCAGGAAAGTGCGTCCATGGCTAACAGAAGCAGGTACCAATCCCGCAAAGTAGAGAACATGAAAGCAACAATAGTCCAAAGGCTGAAGGGTGGTTGCAAACTTTATTTAATGTCGGAGACCCCGAAAGTTCCTACCTACCGAACTACATATCCGGCACCAGTGTACTCACCCCCAATCAATATCCGACTGTCCAACCCCGAGTCTGCTGTGGCAGCGTGCAATGAGTTCCTAGCAAGGAACTATCCGACAGTTGCGTCGTACCAAATCACCGATGAGTACGATGCATACCTAGACATGGTGGACGGGTCGGAAAGTTGCCTTGACCGGGCGACGTTCAACCCATCAAAGCTTAGAAGTTATCCAAAACAGCACTCCTACCATGCACCCACAATCAGAAGTGCCGTACCTTCCCCGTTCCAGAACACGCTGCAGAACGTACTGGCTGCTGCCACGAAAAGAAATTGCAACGTCACACAGATGAGAGAACTGCCTACTTTGGATTCAGCGGTATTTAATGTTGAGTGCTTTAAAAAATTTGCGTGCAATCAAGAATACTGGAAGGAATTTGCCGCCAGCCCTATTAGGATAACGACTGAGAACTTGACAACTTATGTCACAAAACTAAAAGGACCAAAAGCAGCAGCACTGTTTGCCAAGACACATAACCTGCTACCACTGCAGGAGGTGCCGATGGACAGGTTTACTGTAGACATGAAAAGGGACGTGAAGGTGACTCCGGGGACGAAGCACACTGAGGAAAGACCTAAAGTGCAGGTCATACAGGCAGCCGAACCTTTGGCAACAGCATATCTGTGTGGGATCCACAGAGAGTTGGTCAGAAGGCTGAATGCAGTCCTTCTACCTAATGTACACACGCTGTTTGACATGTCTGCCGAGGACTTTGACGCCATTATTGCCGCGCACTTCAAGCCGGGGGACGCCGTATTGGAAACCGATATAGCCTCCTTTGACAAGAGCCAAGACGACTCATTGGCGCTCACTGCTCTAATGTTGCTAGAGGATTTGGGGGTGGATCATCCCCTGTTGGACTTGATAGAGGCTGCCTTCGGGGAGATCTCCAGCTGCCACCTACCGACGGGCACCCGTTTTAAGTTCGGCGCCATGATGAAGTCTGGTATGTTCCTAACCCTGTTCGTCAACACACTGCTAAACATCACCATAGCCAGCCGAGTGCTGGAGGACCGCTTGACAAGGTCTGCGTGCGCGGCCTTCATCGGCGACGACAATATAATACATGGGGTTGTCTCTGACGAACTGATGGCAGCAAGGTGTGCTACATGGATGAACATGGAAGTGAAGATCATAGATGCGGTCGTGTCTCAGAAAGCCCCGTACTTCTGCGGAGGGTTTATACTGTATGACACAGTAGCAGGCACGGCCTGCAGAGTGGCAGACCCGCTAAAGCGGCTGTTCAAGCTGGGCAAACCGCTGGCAGCGGGAGATGAACAAGACGACGACAGAAGACGTGCACTGGCTGACGAAGTGGTTAGATGGCAACGAACAGGACTAACTGATGAGCTAGAAAAAGCGGTACACTCCAGGTATGAAGTGCAGGGCATATCTGTCGTGGTAATGTCTATGGCCACCTTTGCAAGCTCTAGATCTAACTTTGAGAAGCTCAGAGGACCCGTCGTAACCCTGTACGGTGGTCCTAAATAGGTACGCACTACAGCTACCTATTTCGTCAGAAACCAATCGCAGCTACTTGCATACCTACCAGCTACAATGGAGTTCATCCCGACGCAAACTTTCTATAACAGAAGGTACCAACCCCGACCCTGGGCCCCACGCCCTACAATTCAAGTAATTAGACCTAGACCACGTCCACAGAGGCAGGCTGGGCAACTCGCCCAGCTGATCTCCGCAGTCAACAAATTGACCATGCGCGCGGTACCTCAACAGAAGCCTCGCAGAAATCGGAAAAACAAGAAGCAAAGGCAGAAGAAGCAGGCGCCGCAAAACGACCCAAAGCAAAAGAAGCAACCACCACAAAAGAAGCCGGCTCAAAAGAAGAAGAAACCAGGCCGTAGGGAGAGAATGTGCATGAAAATTGAAAATGATTGCATCTTCGAAGTCAAGCATGAAGGCAAAGTGATGGGCTACGCATGCCTGGTGGGGGATAAAGTAATGAAACCAGCACATGTGAAGGGAACTATCGACAATGCCGATCTGGCTAAACTGGCCTTTAAGCGGTCGTCTAAATACGATCTTGAATGTGCACAGATACCGGTGCACATGAAGTCTGATGCCTCGAAGTTTACCCACGAGAAACCCGAGGGGTACTATAACTGGCATCACGGAGCAGTGCAGTATTCAGGAGGCCGGTTCACTATCCCGACGGGTGCAGGCAAGCCGGGAGACAGCGGCAGACCGATCTTCGACAACAAAGGACGGGTGGTGGCCATCGTCCTAGGAGGGGCCAACGAAGGTGCCCGCACGGCCCTCTCCGTGGTGACGTGGAACAAAGACATCGTCACAAAAATTACCCCTGAGGGAGCCGAAGAGTGGAGCCTCGCCCTCCCGGTCTTGTGCCTGTTGGCAAACACTACATTCCCCTGCTCTCAGCCGCCTTGCACACCCTGCTGCTACGAAAAGGAACCGGAAAGCACCTTGCGCATGCTTGAGGACAACGTGATGAGACCCGGATACTACCAGCTACTAAAAGCATCGCTGACTTGCTCTCCCCACCGCCAAAGACGCAGTACTAAGGACAATTTTAATGTCTATAAAGCCACAAGACCATATCTAGCTCATTGTCCTGACTGCGGAGAAGGGCATTCGTGCCACAGCCCTATCGCATTGGAGCGCATCAGAAATGAAGCAACGGACGGAACGCTGAAAATCCAGGTCTCTTTGCAGATCGGGATAAAGACAGATGACAGCCACGATTGGACCAAGCTGCGCTATATGGATAGCCATACGCCAGCGGACGCGGAGCGAGCCGGATTGCTTGTAAGGACTTCAGCACCGTGCACGATCACCGGGACCATGGGACACTTTATTCTCGCCCGATGCCCGAAAGGAGAGACGCTGACAGTGGGATTTACGGACAGCAGAAAGATCAGCCACACATGCACACACCCGTTCCATCATGAACCACCTGTGATAGGTAGGGAGAGGTTCCACTCTCGACCACAACATGGTAAAGAGTTACCTTGCAGCACGTACGTGCAGAGCACCGCTGCCACTGCTGAGGAGATAGAGGTGCATATGCCCCCAGATACTCCTGACCGCACGCTGATGACGCAGCAGTCTGGCAACGTGAAGATCACAGTTAATGGGCAGACGGTGCGGTACAAGTGCAACTGCGGTGGCTCAAACGAGGGACTGACAACCACAGACAAAGTGATCAATAACTGCAAAATTGATCAGTGCCATGCTGCAGTCACTAATCACAAGAAGTGGCAATACAACTCCCCTTTAGTCCCGCGCAACGCTGAACTCGGGGACCGTAAAGGAAAGATCCACATCCCATTCCCATTGGCAAACGTGACTTGCAGAGTGCCAAAAGCAAGAAACCCTACAGTAACTTACGGAAAAAACCAAGTCACCATGCTGCTGTATCCTGACCATCCGACACTCTTGTCTTACCGTAACATGGGACAGGAACCAAATTACCACGAGGAGTGGGTGACACACAAGAAGGAGGTTACCTTGACCGTGCCTACTGAGGGTCTGGAGGTCACTTGGGGCAACAACGAACCATACAAGTACTGGCCGCAGATGTCTACGAACGGTACTGCTCATGGTCACCCACATGAGATAATCTTGTACTATTATGAGCTGTACCCCACTATGACTGTAGTCATTGTGTCGGTGGCCTCGTTCGTGCTTCTGTCGATGGTGGGCACAGCAGTGGGAATGTGTGTGTGCGCACGGCGCAGATGCATTACACCATATGAATTAACACCAGGAGCCACTGTTCCCTTCCTGCTCAGCCTGCTATGCTGCGTCAGAACGACCAAGGCGGCCACATATTACGAGGCTGCGGCATATCTATGGAACGAACAGCAGCCCCTGTTCTGGTTGCAGGCTCTTATCCCGCTGGCCGCCTTGATCGTCCTGTGCAACTGTCTGAAACTCTTGCCATGCTGCTGTAAGACCCTGGCTTTTTTAGCCGTAATGAGCATCGGTGCCCACACTGTGAGCGCGTACGAACACGTAACAGTGATCCCGAACACGGTGGGAGTACCGTATAAGACTCTTGTCAACAGACCGGGTTACAGCCCCATGGTGTTGGAGATGGAGCTACAATCAGTCACCTTGGAACCAACACTGTCACTTGACTACATCACGTGCGAGTACAAAACTGTCATCCCCTCCCCGTACGTGAAGTGCTGTGGTACAGCAGAGTGCAAGGACAAGAGCCTACCAGACTACAGCTGCAAGGTCTTTACTGGAGTCTACCCATTTATGTGGGGCGGCGCCTACTGCTTTTGCGACGCCGAAAATACGCAATTGAGCGAGGCACATGTAGAGAAATCTGAATCTTGCAAAACAGAGTTTGCATCGGCCTACAGAGCCCACACCGCATCGGCGTCGGCGAAGCTCCGCGTCCTTTACCAAGGAAACAACATTACCGTAGCTGCCTACGCTAACGGTGACCATGCCGTCACAGTAAAGGACGCCAAGTTTGTCGTGGGCCCAATGTCCTCCGCCTGGACACCTTTTGACAACAAAATCGTGGTGTACAAAGGCGACGTCTACAACATGGACTACCCACCTTTTGGCGCAGGAAGACCAGGACAATTTGGTGACATTCAAAGTCGTACACCGGAAAGTAAAGACGTTTATGCCAACACTCAGTTGGTACTACAGAGGCCAGCAGCAGGCACGGTACATGTACCATACTCTCAGGCACCATCTGGCTTCAAGTATTGGCTGAAGGAACGAGGAGCATCGCTACAGCACACGGCACCGTTCGGTTGCCAGATTGCGACAAACCCGGTAAGAGCTGTAAATTGCGCTGTGGGGAACATACCAATTTCCATCGACATACCGGATGCGGCCTTTACTAGGGTTGTCGATGCACCCTCTGTAACGGACATGTCATGCGAAGTACCAGCCTGCACTCACTCCTCCGACTTTGGGGGCGTCGCCATCATCAAATACACAGCTAGCAAGAAAGGTAAATGTGCAGTACATTCGATGACCAACGCCGTTACCATTCGAGAAGCCGACGTAGAAGTAGAGGGGAACTCCCAGCTGCAAATATCCTTCTCAACAGCCCTGGCAAGCGCCGAGTTTCGCGTGCAAGTGTGCTCCACACAAGTACACTGCGCAGCCGCATGCCACCCTCCAAAGGACCACATAGTCAATTACCCAGCATCACACACCACCCTTGGGGTCCAGGATATATCCACAACGGCAATGTCTTGGGTGCAGAAGATTACGGGAGGAGTAGGATTAATTGTTGCTGTTGCTGCCTTAATTTTAATTGTGGTGCTATGCGTGTCGTTTAGCAGGCACTAAACCGATGATAAGGCACGAAATAACTAAATAGCAAAAGTAGAAAGTACATAACCAGGTATATGTGCCCCTTAAGAGGCACAATATATATAGCTAAGCACTATTAGATCAAAGGGCTATACAACCCCTGAATAGTAACAAAACACAAAAACCAATAAAAATCATAAAAAGAAAAATCTCATAAACAGGTATAAGTGTCCCCTAAGAGACACATTGTATGTAGGTAGTAAGTATAGATCAAAGGGCTATATTAACCCCTGAATAGTAACAAAACACAAAAACAATAAAAACTACAAAATAGAAAATCTATAAACAAAAGTAGTTCAAAGGGCTACAAAACCCCTGAATAGTAACAAAACATAAAATGTAATAAAAATTAAGTGTGTACCCAAAAGAGGTACAGTAAGAATCAGTGAATATCACAATTGGCAACGAGAAGAGACGTAGGTATTTAAGCTTCCTAAAAGCAGCCGAACTCACTTTGAGACGTAGGCATAGCATACCGAACTCTTCCACTATTCTCCGAACCCACAGGGACGTAGGAGATGTTATTTTGTTTTTAATATTTCAAAAAAAAAAAAAAAAAAAAAAAAAGCGGCCGCCACCGCGGTGGAGCTCCAGCTTTTGTTCCCTTTAGTGAGGGTTAATTGCGCGCTTGGCGTAATCATGGTCATAGCTGTTTCCTGTGTGAAATTGTTATCCGCTCACAATTCCACACAACATACGAGCCGGAAGCATAAAGTGTAAAGCCTGGGGTGCCTAATGAGTGAGCTAACTCACATTAATTGCGTTGCGCTCACTGCCCGCTTTCCAGTCGGGAAACCTGTCGTGCCAGCTGCATTAATGAATCGGCCAACGCGCGGGGAGAGGCGGTTTGCGTATTGGGCGCTCTTCCGCTTCCTCGCTCACTGACTCGCTGCGCTCGGTCGTTCGGCTGCGGCGAGCGGTATCAGCTCACTCAAAGGCGGTAATACGGTTATCCACAGAATCAGGGGATAACGCAGGAAAGAACATGTGAGCAAAAGGCCAGCAAAAGGCCAGGAACCGTAAAAAGGCCGCGTTGCTGGCGTTTTTCCATAGGCTCCGCCCCCCTGACGAGCATCACAAAAATCGACGCTCAAGTCAGAGGTGGCGAAACCCGACAGGACTATAAAGATACCAGGCGTTTCCCCCTGGAAGCTCCCTCGTGCGCTCTCCTGTTCCGACCCTGCCGCTTACCGGATACCTGTCCGCCTTTCTCCCTTCGGGAAGCGTGGCGCTTTCTCATAGCTCACGCTGTAGGTATCTCAGTTCGGTGTAGGTCGTTCGCTCCAAGCTGGGCTGTGTGCACGAACCCCCCGTTCAGCCCGACCGCTGCGCCTTATCCGGTAACTATCGTCTTGAGTCCAACCCGGTAAGACACGACTTATCGCCACTGGCAGCAGCCACTGGTAACAGGATTAGCAGAGCGAGGTATGTAGGCGGTGCTACAGAGTTCTTGAAGTGGTGGCCTAACTACGGCTACACTAGAAGGACAGTATTTGGTATCTGCGCTCTGCTGAAGCCAGTTACCTTCGGAAAAAGAGTTGGTAGCTCTTGATCCGGCAAACAAACCACCGCTGGTAGCGGTGGTTTTTTTGTTTGCAAGCAGCAGATTACGCGCAGAAAAAAAGGATCTCAAGAAGATCCTTTGATCTTTTCTACGGGGTCTGACGCTCAGTGGAACGAAAACTCACGTTAAGGGATTTTGGTCATGAGATTATCAAAAAGGATCTTCACCTAGATCCTTTTAAATTAAAAATGAAGTTTTAAATCAATCTAAAGTATATATGAGTAAACTTGGTCTGACAGTTACCAATGCTTAATCAGTGAGGCACCTATCTCAGCGATCTGTCTATTTCGTTCATCCATAGTTGCCTGACTCCCCGTCGTGTAGATAACTACGATACGGGAGGGCTTACCATCTGGCCCCAGTGCTGCAATGATACCGCGAGACCCACGCTCACCGGCTCCAGATTTATCAGCAATAAACCAGCCAGCCGGAAGGGCCGAGCGCAGAAGTGGTCCTGCAACTTTATCCGCCTCCATCCAGTCTATTAATTGTTGCCGGGAAGCTAGAGTAAGTAGTTCGCCAGTTAATAGTTTGCGCAACGTTGTTGCCATTGCTACAGGCATCGTGGTGTCACGCTCGTCGTTTGGTATGGCTTCATTCAGCTCCGGTTCCCAACGATCAAGGCGAGTTACATGATCCCCCATGTTGTGCAAAAAAGCGGTTAGCTCCTTCGGTCCTCCGATCGTTGTCAGAAGTAAGTTGGCCGCAGTGTTATCACTCATGGTTATGGCAGCACTGCATAATTCTCTTACTGTCATGCCATCCGTAAGATGCTTTTCTGTGACTGGTGAGTACTCAACCAAGTCATTCTGAGAATAGTGTATGCGGCGACCGAGTTGCTCTTGCCCGGCGTCAATACGGGATAATACCGCGCCACATAGCAGAACTTTAAAAGTGCTCATCATTGGAAAACGTTCTTCGGGGCGAAAACTCTCAAGGATCTTACCGCTGTTGAGATCCAGTTCGATGTAACCCACTCGTGCACCCAACTGATCTTCAGCATCTTTTACTTTCACCAGCGTTTCTGGGTGAGCAAAAACAGGAAGGCAAAATGCCGCAAAAAAGGGAATAAGGGCGACACGGAAATGTTGAATACTCATACTCTTCCTTTTTCAATATTATTGAAGCATTTATCAGGGTTATTGTCTCATGAGCGGATACATATTTGAATGTATTTAGAAAAATAAACAAATAGGGGTTCCGCGCACATTTCCCCGAAAAGTGCCAC

>pCHIKV.b

CTAAATTGTAAGCGTTAATATTTTGTTAAAATTCGCGTTAAATTTTTGTTAAATCAGCTCATTTTTTAACCAATAGGCCGAAATCGGCAAAATCCCTTATAAATCAAAAGAATAGACCGAGATAGGGTTGAGTGTTGTTCCAGTTTGGAACAAGAGTCCACTATTAAAGAACGTGGACTCCAACGTCAAAGGGCGAAAAACCGTCTATCAGGGCGATGGCCCACTACGTGAACCATCACCCTAATCAAGTTTTTTGGGGTCGAGGTGCCGTAAAGCACTAAATCGGAACCCTAAAGGGAGCCCCCGATTTAGAGCTTGACGGGGAAAGCCGGCGAACGTGGCGAGAAAGGAAGGGAAGAAAGCGAAAGGAGCGGGCGCTAGGGCGCTGGCAAGTGTAGCGGTCACGCTGCGCGTAACCACCACACCCGCCGCGCTTAATGCGCCGCTACAGGGCGCGTCCCATTCGCCATTCAGGCTGCGCAACTGTTGGGAAGGGCGATCGGTGCGGGCCTCTTCGCTATTACGCCAGCTGGCGAAAGGGGGATGTGCTGCAAGGCGATTAAGTTGGGTAACGCCAGGGTTTTCCCAGTCACGACGTTGTAAAACGACGGCCAGTGAGCGCGCGTAATACGACTCACTATAGGGCGAATTGGGTACCGGGCCCCCCCTCGAGTAATACGACTCACTATAATGGCTGCGTGAGACACACGTAGCCTACCAGTTTCTTACTGCTCTACTCTGCTTAGCAAGAGACTTGAGAACCCATCATGGATCCCGTGTACGTGGACATAGACGCCGACAGCGCCTTTTTAAAGGCCCTGCAGCGTGCGTACCCCATGTTTGAGGTGGAACCAAGGCAGGTCACACCGAATGACCATGCCAATGCTAGAGCATTCTCGCATCTAGCTATAAAACTAATAGAGCAGGAAATTGATCCCGACTCAACCATCCTGGACATAGGCAGCGCGCCAGCAAGGAGGATGATGTCGGATAGGAAGTACCACTGCGTTTGCCCTATGCGCAGCGCAGAAGACCCTGAGAGACTCGCCAACTACGCGAGAAAACTAGCATCTGCCGCAGGAAAAGTCTTGGACAGAAACATCTCCGAAAAAATTGGAGATCTACAAGCAGTAATGGCTGTACCAGACGCAGAAACGCCCACATTCTGCTTGCACACTGACGTCTCATGTAGACAAAGGGCGGACGTCGCTATATACCAGGATGTCTACGCCGTGCATGCACCAACATCGCTGTACCACCAGGCGATTAAAGGAGTCCGTGTAGCATACTGGATAGGGTTTGATACAACCCCGTTCATGTATAATGCCATGGCAGGTGCATACCCCTCGTACTCGACAAACTGGGCAGATGAGCAGGTGCTGAAGGCAAAGAACATAGGATTATGTTCAACAGACCTGACGGAAGGTAGACGAGGTAAATTGTCTATCATGAGAGGAAAAAAGATGAAGCCATGTGACCGCGTACTGTTCTCAGTCGGGTCAACGCTTTACCCGGAGAGCCGTAAGCTTCTTAAGAGTTGGCACTTACCTTCAGTGTTCCATCTAAAAGGGAAGCTCAGCTTCACGTGCCGCTGTGATACAGTGGTTTCGTGTGAAGGCTATGTCGTTAAGAGAATAACGATTAGCCCGGGCCTCTACGGTAAAACCACAGGGTACGCAGTAACCCACCATGCAGACGGATTCCTAATGTGCAAAACAACCGATACGGTAGATGGCGAGAGAGTGTCATTTTCGGTATGCACGTACGTACCCGCAACCATTTGTGATCAAATGACAGGTATTCTTGCCACGGAGGTTACACCGGAGGATGCACAGAAGCTGCTGGTGGGACTGAACCAGAGGATAGTGGTCAATGGCAGAACGCAGAGGAACACGAACACAATGAAGAATTACTTGCTTCCTGTAGTTGCCCAAGCCTTCAGTAAGTGGGCAAAGGAATGCCGGAAAGATATGGAAGATGAAAAACTTTTGGGCATCAGAGAAAGGACACTGACATGCTGCTGCCTTTGGGCGTTCAAGAAGCAGAAGACACACACGGTCTACAAGAGGCCTGACACTCAGTCAATTCAGAAAGTCCCAGCCGAATTTGACAGCTTTGTGGTACCAAGTCTGTGGTCATCTGGACTGTCGATCCCGCTACGGACCAGAATCAAGTGGCTGCTAAGCAAAGTGCCAAAGACTGATTTGATCCCTTACAGCGGTGACGCCAAAGAAGCCCGCGACGCTGAAAAAGAAGCAGAAGAAGAACGAGAAGCGGAGCTAACTCGCGAGGCACTACCACCACTACAGGCGGCACAGGACGACGTCCAGGTCGAAATTGACGTGGAACAGCTCGAAGACAGAGCTGGGGCAGGAATAATTGAAACTCCAAGAGGAGCTATCAAAGTCACTGCCCAACCAACAGACCACGTCGTGGGAGAGTACTTGGTACTTTCCCCGCAGACCGTGTTACGAAGCCAGAAGCTCAGCCTGATCCACGCATTGGCGGAACAAGTGAAGACATGCACACACAGCGGACGGGCAGGAAGGTACGCGGTCGAAGCATATGACGGCAGAATCCTTGTGCCCTCAGGCTATGCAATATCACCTGAAGACTTCCAGAGCCTGAGCGAAAGTGCGACGATGGTGTACAACGAAAGGGAGTTCGTAAATAGGAAATTACACCATATCGCGTTGCACGGACCAGCCCTGAACACTGACGAGGAGTCGTACGAGCTGGTAAGGGCAGAAAGGACAGAGCATGAGTACGTCTATGATGTGGACCAAAGAAGGTGCTGCAAGAAAGAGGAGGCAGCCGGGCTGGTACTGGTCGGCGACTTGACCAACCCGCCCTACCATGAGTTCGCATATGAAGGGCTGAGAATCCGCCCCGCCTGCCCATACAAGACCGCAGTAATAGGGGTCTTTGGAGTGCCAGGATCCGGCAAATCAGCAATCATTAAGAACCTAGTTACCAGGCAAGACCTAGTGACCAGTGGAAAGAAAGAAAACTGCCAAGAAATCTCCACCGACGTGATGCGACAGAGGAACCTGGAGATATCTGCACGCACGGTCGACTCACTGCTCTTGAACGGATGCAATAGACCAGTCGACGTGTTGTACGTCGACGAAGCTTTTGCGTGCCATTCTGGCACGCTACTTGCTCTGATAGCCTTGGTGAGACCGAGGCAGAAAGTCGTGCTATGCGGTGATCCGAAACAGTGCGGCTTCTTCAATATGATGCAGATGAAAGTTAACTACAACCATAACATCTGCACCCAAGTGTACCATAAAAGTATTTCCAGGCGGTGTACACTGCCTGTGACTGCCATTGTGTCCTCGTTGCATTACGAAGGCAAAATGCGCACAACAAATGAGTACAACAAGCCAATTGTAGTGGATACTACAGGCTCGACAAAACCCGACCCCGGAGACCTTGTGCTAACATGTTTCAGAGGGTGGGTTAAGCAACTGCAAATTGACTATCGTGGACACGAGGTCATGACAGCAGCTGCATCTCAGGGGCTAACCAGAAAAGGGGTCTATGCCGTCAGGCAAAAAGTTAATGAAAACCCCCTTTACGCATCAACATCAGAGCACGTGAACGTGCTACTGACGCGTACGGAAGGCAAACTAGTATGGAAGACACTTTCTGGAGACCCATGGATAAAGACACTGCAGAACCCGCCGAAAGGAAATTTTAAAGCAACAATTAAGGAATGGGAAGTGGAACATGCTTCAATAATGGCGGGTATCTGTAACCACCAAGTGACCTTTGACACGTTCCAGAATAAAGCCAATGTCTGCTGGGCGAAGAGCTTAGTCCCCATCCTAGAAACAGCAGGGATAAAATTAAACGACAGGCAGTGGTCCCAGATAATCCAGGCTTTTAAAGAAGACAGAGCATACTCACCCGAGGTGGCCCTGAATGAGATATGCACGCGCATGTACGGGGTAGACCTGGACAGCGGACTGTTCTCTAAACCACTGGTGTCCGTGCATTATGCGGATAATCACTGGGACAACAGGCCGGGAGGGAAGATGTTCGGATTCAACCCCGAAGCGGCGTCCATACTGGAGAGGAAATACCCGTTTACAAAAGGGAAGTGGAATACCAACAAGCAAATCTGTGTGACTACTAGGAGGATTGAAGATTTTAACCCGAACACCAACATTATACCTGCCAACAGGAGATTACCGCATTCATTGGTGGCCGAACATCGCCCGGTAAAAGGGGAGAGGATGGAATGGTTGGTCAACAAAATAAATGGCCACCATGTGCTCCTGGTCAGCGGCTACAACCTCGTTCTGCCCACTAAGAGAGTCACCTGGGTGGCGCCGCTGGGCATTCGGGGAGCTGACTACACATACAACCTAGAGTTAGGCCTACCAGCAACGCTCGGTAGATATGACCTAGTGATTATAAACATCCACACACCCTTTCGCATACATCATTACCAACAGTGCGTGGATCACGCAATGAAGCTGCAGATGCTCGGAGGAGACTCCCTGAGACTGCTCAAGCCGGGTGGTTCATTACTGATCAGGGCATACGGCTACGCAGACAGAACAAGCGAACGAGTAGTCTGCGTATTGGGACGCAAGTTTCGATCATCCAGAGCGTTGAAACCGCCGTGCGTCACTAGCAACACCGAGATGTTTTTCTTGTTCAGCAACTTTGATAACGGCAGAAGGAACTTTACGACGCACGTAATGAACAACCAGCTGAATGCTGCTTTTGTTGGTCAGGCCACCCGAGCAGGGTGCGCACCGTCGTACCGGGTTAAACGCATGGACATCGCAAAGAACGATGAAGAGTGTGTAGTCAACGCCGCCAACCCTCGTGGGCTACCAGGCGATGGCGTCTGTAAAGCAGTATACAAAAAATGGCCGGAGTCCTTCAAGAACAGTGCAACACCAGTGGGAACCGCAAAGACAGTCATGTGCGGTACATACCCGGTAATCCATGCAGTAGGACCTAATTTCTCAAATTACTCTGAGTCCGAAGGAGACCGGGAATTGGCAGCTGCTTACCGAGAAGTCGCTAAGGAGGTGACTAGACTAGGAGTAAACAGCGTAGCTATACCGCTCCTTTCCACCGGTGTGTACTCTGGAGGGAAAGACAGGCTGACTCAGTCACTAAACCACCTTTTTACAGCATTAGACTCAACTGATGCAGATGTGGTTATCTACTGCCGCGACAAGGAGTGGGAGAAGAAAATAGCTGAGGCCATACAAATGAGGACCCAAGTGGAATTACTAGACGAACACATCTCTGTAGACTGCGATATCATCCGAGTGCACCCTGACAGCAGTTTGGCAGGTAGAAAAGGGTACAGCACTACAGAAGGTTCACTGTACTCCTACTTGGAAGGGACACGGTTCCATCAGACGGCAGTGGACATGGCAGAAGTATACACCATGTGGCCAAAGCAGACGGAGGCTAATGAACAAGTTTGCTTGTACGCATTGGGGGAAAGTATAGAATCAATCAGGCAAAAGTGCCCAGTGGATGACGCAGATGCATCGTCGCCCCCAAAAACCGTCCCGTGCCTCTGCCGTTATGCCATGACACCCGAACGAGTCACCAGGCTTCGTATGAACCATGTCACAAGCATAATAGTATGCTCATCATTCCCCCTTCCAAAGTATAAAATAGAAGGAGTGCAGAAAGTCAAGTGTTCTAAAGTGATGCTGTTCGACCATAACGTGCCATCACGCGTTAGTCCAAGGGAATATAAATCGCCTCAGGAGACCGCACAAGAAGTAAGTTCGACCACGTCACTGACGCACAGCCAATTCGACCTTAGCGTTGACGGTGAGGAACTGCCCGCTCCGTCTGACTTGGAAGCTGACGCTCCGATTCCGGAACCAACACCAGACGACAGAGCGGTACTTACTTTGCCTCCCACGATTGATAATTTTTCGGCTGTGTCAGACTGGGTAATGAATACCGCGCCAGTCGCACCACCCAGAAGAAGACGTGGGAAAAACTTGAATGTCACCTGCGACGAGAGAGAAGGGAACGTACTTCCCATGGCTAGCGTTCGGTTCTTCAGAGCGGATCTGCACTCCATCGTACAGGAAACGGCAGAGATACGCGATACGGCCGCGTCCCTCCAGGCGCCCCTGAGTGTCGCTACAGAACCGAATCAACTGCCGATCTCATTTGGAGCACCAAACGAGACTTTCCCCATAACGTTCGGGGATTTTGATGAAGGGGAGATTGAAAGCTTGTCCTCTGAGTTACTGACCTTTGGGGACTTCTCGCCGGGCGAAGTGGATGACCTGACAGACAGCGACTGGTCCACGTGTTCAGACACGGACGACGAATTATGACTAGATAGGGCAGGTGGGTACATATTCTCATCTGACACCGGCCCCGGCCACCTGCAACAGAGGTCTGTCCGTCAGACAGTACTGCCGGTAAATACCTTGGAGGAAGTTCAGGAGGAGAAATGTTACCCACCTAAGTTGGATGAAGTGAAAGAGCAGTTGTTACTTAAGAAACTCCAGGAAAGTGCGTCCATGGCTAACAGAAGCAGGTACCAATCCCGCAAAGTAGAGAACATGAAAGCAACAATAGTCCAAAGGCTGAAGGGTGGTTGCAAACTTTATTTAATGTCGGAGACCCCGAAAGTTCCTACCTACCGAACTACATATCCGGCACCAGTGTACTCACCCCCAATCAATATCCGACTGTCCAACCCCGAGTCTGCTGTGGCAGCGTGCAATGAGTTCCTAGCAAGGAACTATCCGACAGTTGCGTCGTACCAAATCACCGATGAGTACGATGCATACCTAGACATGGTGGACGGGTCGGAAAGTTGCCTTGACCGGGCGACGTTCAACCCATCAAAGCTTAGAAGTTATCCAAAACAGCACTCCTACCATGCACCCACAATCAGAAGTGCCGTACCTTCCCCGTTCCAGAACACGCTGCAGAACGTACTGGCTGCTGCCACGAAAAGAAATTGCAACGTCACACAGATGAGAGAACTGCCTACTTTGGATTCAGCGGTATTTAATGTTGAGTGCTTTAAAAAATTTGCGTGCAATCAAGAATACTGGAAGGAATTTGCCGCCAGCCCTATTAGGATAACGACTGAGAACTTGACAACTTATGTCACAAAACTAAAAGGACCAAAAGCAGCAGCACTGTTTGCCAAGACACATAACCTGCTACCACTGCAGGAGGTGCCGATGGACAGGTTTACTGTAGACATGAAAAGGGACGTGAAGGTGACTCCGGGGACGAAGCACACTGAGGAAAGACCTAAAGTGCAGGTCATACAGGCAGCCGAACCTTTGGCAACAGCATATCTGTGTGGGATCCACAGAGAGTTGGTCAGAAGGCTGAATGCAGTCCTTCTACCTAATGTACACACGCTGTTTGACATGTCTGCCGAGGACTTTGACGCCATTATTGCCGCGCACTTCAAGCCGGGGGACGCCGTATTGGAAACCGATATAGCCTCCTTTGACAAGAGCCAAGACGACTCATTGGCGCTCACTGCTCTAATGTTGCTAGAGGATTTGGGGGTGGATCATCCCCTGTTGGACTTGATAGAGGCTGCCTTCGGGGAGATCTCCAGCTGCCACCTACCGACGGGCACCCGTTTTAAGTTCGGCGCCATGATGAAGTCTGGTATGTTCCTAACCCTGTTCGTCAACACACTGCTAAACATCACCATAGCCAGCCGAGTGCTGGAGGACCGCTTGACAAGGTCTGCGTGCGCGGCCTTCATCGGCGACGACAATATAATACATGGGGTTGTCTCTGACGAACTGATGGCAGCAAGGTGTGCTACATGGATGAACATGGAAGTGAAGATCATAGATGCGGTCGTGTCTCAGAAAGCCCCGTACTTCTGCGGAGGGTTTATACTGTATGACACAGTAGCAGGCACGGCCTGCAGAGTGGCAGACCCGCTAAAGCGGCTGTTCAAGCTGGGCAAACCGCTGGCAGCGGGAGATGAACAAGACGACGACAGAAGACGTGCACTGGCTGACGAAGTGGTTAGATGGCAACGAACAGGACTAACTGATGAGCTAGAAAAAGCGGTACACTCCAGGTATGAAGTGCAGGGCATATCTGTCGTGGTAATGTCTATGGCCACCTTTGCAAGCTCTAGATCTAACTTTGAGAAGCTCAGAGGACCCGTCGTAACCCTGTACGGTGGTCCTAAATAGGTACGCACTACAGCTACCTATTTCGTCAGAAACCAATCGCAGCTACTTGCATACCTACCAGCTACAATGGAGTTCATCCCGACGCAAACTTTCTATAACAGAAGGTACCAACCCCGACCCTGGGCCCCACGCCCTACAATTCAAGTAATTAGACCTAGACCACGTCCACAGAGGCAGGCTGGGCAACTCGCCCAGCTGATCTCCGCAGTCAACAAATTGACCATGCGCGCGGTACCTCAACAGAAGCCTCGCAGAAATCGGAAAAACAAGAAGCAAAGGCAGAAGAAGCAGGCGCCGCAAAACGACCCAAAGCAAAAGAAGCAACCACCACAAAAGAAGCCGGCTCAAAAGAAGAAGAAACCAGGCCGTAGGGAGAGAATGTGCATGAAAATTGAAAATGATTGCATCTTCGAAGTCAAGCATGAAGGCAAAGTGATGGGCTACGCATGCCTGGTGGGGGATAAAGTAATGAAACCAGCACATGTGAAGGGAACTATCGACAATGCCGATCTGGCTAAACTGGCCTTTAAGCGGTCGTCTAAATACGATCTTGAATGTGCACAGATACCGGTGCACATGAAGTCTGATGCCTCGAAGTTTACCCACGAGAAACCCGAGGGGTACTATAACTGGCATCACGGAGCAGTGCAGTATTCAGGAGGCCGGTTCACTATCCCGACGGGTGCAGGCAAGCCGGGAGACAGCGGCAGACCGATCTTCGACAACAAAGGACGGGTGGTGGCCATCGTCCTAGGAGGGGCCAACGAAGGTGCCCGCACGGCCCTCTCCGTGGTGACGTGGAACAAAGACATCGTCACAAAAATTACCCCTGAGGGAGCCGAAGAGTGGAGCCTCGCCCTCCCGGTCTTGTGCCTGTTGGCAAACACTACATTCCCCTGCTCTCAGCCGCCTTGCACACCCTGCTGCTACGAAAAGGAACCGGAAAGCACCTTGCGCATGCTTGAGGACAACGTGATGAGACCCGGATACTACCAGCTACTAAAAGCATCGCTGACTTGCTCTCCCCACCGCCAAAGACGCAGTACTAAGGACAATTTTAATGTCTATAAAGCCACAAGACCATATCTAGCTCATTGTCCTGACTGCGGAGAAGGGCATTCGTGCCACAGCCCTATCGCATTGGAGCGCATCAGAAATGAAGCAACGGACGGAACGCTGAAAATCCAGGTCTCTTTGCAGATCGGGATAAAGACAGATGACAGCCACGATTGGACCAAGCTGCGCTATATGGATAGCCATACGCCAGCGGACGCGGAGCGAGCCGGATTGCTTGTAAGGACTTCAGCACCGTGCACGATCACCGGGACCATGGGACACTTTATTCTCGCCCGATGCCCGAAAGGAGAGACGCTGACAGTGGGATTTACGGACAGCAGAAAGATCAGCCACACATGCACACACCCGTTCCATCATGAACCACCTGTGATAGGTAGGGAGAGGTTCCACTCTCGACCACAACATGGTAAAGAGTTACCTTGCAGCACGTACGTGCAGAGCACCGCTGCCACTGCTGAGGAGATAGAGGTGCATATGCCCCCAGATACTCCTGACCGCACGCTGATGACGCAGCAGTCTGGCAACGTGAAGATCACAGTTAATGGGCAGACGGTGCGGTACAAGTGCAACTGCGGTGGCTCAAACGAGGGACTGACAACCACAGACAAAGTGATCAATAACTGCAAAATTGATCAGTGCCATGCTGCAGTCACTAATCACAAGAAGTGGCAATACAACTCCCCTTTAGTCCCGCGCAACGCTGAACTCGGGGACCGTAAAGGAAAGATCCACATCCCATTCCCATTGGCAAACGTGACTTGCAGAGTGCCAAAAGCAAGAAACCCTACAGTAACTTACGGAAAAAACCAAGTCACCATGCTGCTGTATCCTGACCATCCGACACTCTTGTCTTACCGTAACATGGGACAGGAACCAAATTACCACGAGGAGTGGGTGACACACAAGAAGGAGGTTACCTTGACCGTGCCTACTGAGGGTCTGGAGGTCACTTGGGGCAACAACGAACCATACAAGTACTGGCCGCAGATGTCTACGAACGGTACTGCTCATGGTCACCCACATGAGATAATCTTGTACTATTATGAGCTGTACCCCACTATGACTGTAGTCATTGTGTCGGTGGCCTCGTTCGTGCTTCTGTCGATGGTGGGCACAGCAGTGGGAATGTGTGTGTGCGCACGGCGCAGATGCATTACACCATATGAATTAACACCAGGAGCCACTGTTCCCTTCCTGCTCAGCCTGCTATGCTGCGTCAGAACGACCAAGGCGGCCACATATTACGAGGCTGCGGCATATCTATGGAACGAACAGCAGCCCCTGTTCTGGTTGCAGGCTCTTATCCCGCTGGCCGCCTTGATCGTCCTGTGCAACTGTCTGAAACTCTTGCCATGCTGCTGTAAGACCCTGGCTTTTTTAGCCGTAATGAGCATCGGTGCCCACACTGTGAGCGCGTACGAACACGTAACAGTGATCCCGAACACGGTGGGAGTACCGTATAAGACTCTTGTCAACAGACCGGGTTACAGCCCCATGGTGTTGGAGATGGAGCTACAATCAGTCACCTTGGAACCAACACTGTCACTTGACTACATCACGTGCGAGTACAAAACTGTCATCCCCTCCCCGTACGTGAAGTGCTGTGGTACAGCAGAGTGCAAGGACAAGAGCCTACCAGACTACAGCTGCAAGGTCTTTACTGGAGTCTACCCATTTATGTGGGGCGGCGCCTACTGCTTTTGCGACGCCGAAAATACGCAATTGAGCGAGGCACATGTAGAGAAATCTGAATCTTGCAAAACAGAGTTTGCATCGGCCTACAGAGCCCACACCGCATCGGCGTCGGCGAAGCTCCGCGTCCTTTACCAAGGAAACAACATTACCGTAGCTGCCTACGCTAACGGTGACCATGCCGTCACAGTAAAGGACGCCAAGTTTGTCGTGGGCCCAATGTCCTCCGCCTGGACACCTTTTGACAACAAAATCGTGGTGTACAAAGGCGACGTCTACAACATGGACTACCCACCTTTTGGCGCAGGAAGACCAGGACAATTTGGTGACATTCAAAGTCGTACACCGGAAAGTAAAGACGTTTATGCCAACACTCAGTTGGTACTACAGAGGCCAGCAGCAGGCACGGTACATGTACCATACTCTCAGGCACCATCTGGCTTCAAGTATTGGCTGAAGGAACGAGGAGCATCGCTACAGCACACGGCACCGTTCGGTTGCCAGATTGCGACAAACCCGGTAAGAGCTGTAAATTGCGCTGTGGGGAACATACCAATTTCCATCGACATACCGGATGCGGCCTTTACTAGGGTTGTCGATGCACCCTCTGTAACGGACATGTCATGCGAAGTACCAGCCTGCACTCACTCCTCCGACTTTGGGGGCGTCGCCATCATCAAATACACAGCTAGCAAGAAAGGTAAATGTGCAGTACATTCGATGACCAACGCCGTTACCATTCGAGAAGCCGACGTAGAAGTAGAGGGGAACTCCCAGCTGCAAATATCCTTCTCAACAGCCCTGGCAAGCGCCGAGTTTCGCGTGCAAGTGTGCTCCACACAAGTACACTGCGCAGCCGCATGCCACCCTCCAAAGGACCACATAGTCAATTACCCAGCATCACACACCACCCTTGGGGTCCAGGATATATCCACAACGGCAATGTCTTGGGTGCAGAAGATTACGGGAGGAGTAGGATTAATTGTTGCTGTTGCTGCCTTAATTTTAATTGTGGTGCTATGCGTGTCGTTTAGCAGGCACTAAACCGATGATAAGGCACGAAATAACTAAATAGCAAAAGTAGAAAGTACATAACCAGGTATATGTGCCCCTTAAGAGGCACAATATATATAGCTAAGCACTATTAGATCAAAGGGCTATACAACCCCTGAATAGTAACAAAACACAAAAACCAATAAAAATCATAAAAAGAAAAATCTCATAAACAGGTATAAGTGTCCCCTAAGAGACACATTGTATGTAGGTAGTAAGTATAGATCAAAGGGCTATATTAACCCCTGAATAGTAACAAAACACAAAAACAATAAAAACTACAAAATAGAAAATCTATAAACAAAAGTAGTTCAAAGGGCTACAAAACCCCTGAATAGTAACAAAACATAAAATGTAATAAAAATTAAGTGTGTACCCAAAAGAGGTACAGTAAGAATCAGTGAATATCACAATTGGCAACGAGAAGAGACGTAGGTATTTAAGCTTCCTAAAAGCAGCCGAACTCACTTTGAGACGTAGGCATAGCATACCGAACTCTTCCACTATTCTCCGAACCCACAGGGACGTAGGAGATGTTATTTTGTTTTTAATATTTCAAAAAAAAAAAAAAAAAAAAAAAAAGCGGCCGCCACCGCGGTGGAGCTCCAGCTTTTGTTCCCTTTAGTGAGGGTTAATTGCGCGCTTGGCGTAATCATGGTCATAGCTGTTTCCTGTGTGAAATTGTTATCCGCTCACAATTCCACACAACATACGAGCCGGAAGCATAAAGTGTAAAGCCTGGGGTGCCTAATGAGTGAGCTAACTCACATTAATTGCGTTGCGCTCACTGCCCGCTTTCCAGTCGGGAAACCTGTCGTGCCAGCTGCATTAATGAATCGGCCAACGCGCGGGGAGAGGCGGTTTGCGTATTGGGCGCTCTTCCGCTTCCTCGCTCACTGACTCGCTGCGCTCGGTCGTTCGGCTGCGGCGAGCGGTATCAGCTCACTCAAAGGCGGTAATACGGTTATCCACAGAATCAGGGGATAACGCAGGAAAGAACATGTGAGCAAAAGGCCAGCAAAAGGCCAGGAACCGTAAAAAGGCCGCGTTGCTGGCGTTTTTCCATAGGCTCCGCCCCCCTGACGAGCATCACAAAAATCGACGCTCAAGTCAGAGGTGGCGAAACCCGACAGGACTATAAAGATACCAGGCGTTTCCCCCTGGAAGCTCCCTCGTGCGCTCTCCTGTTCCGACCCTGCCGCTTACCGGATACCTGTCCGCCTTTCTCCCTTCGGGAAGCGTGGCGCTTTCTCATAGCTCACGCTGTAGGTATCTCAGTTCGGTGTAGGTCGTTCGCTCCAAGCTGGGCTGTGTGCACGAACCCCCCGTTCAGCCCGACCGCTGCGCCTTATCCGGTAACTATCGTCTTGAGTCCAACCCGGTAAGACACGACTTATCGCCACTGGCAGCAGCCACTGGTAACAGGATTAGCAGAGCGAGGTATGTAGGCGGTGCTACAGAGTTCTTGAAGTGGTGGCCTAACTACGGCTACACTAGAAGGACAGTATTTGGTATCTGCGCTCTGCTGAAGCCAGTTACCTTCGGAAAAAGAGTTGGTAGCTCTTGATCCGGCAAACAAACCACCGCTGGTAGCGGTGGTTTTTTTGTTTGCAAGCAGCAGATTACGCGCAGAAAAAAAGGATCTCAAGAAGATCCTTTGATCTTTTCTACGGGGTCTGACGCTCAGTGGAACGAAAACTCACGTTAAGGGATTTTGGTCATGAGATTATCAAAAAGGATCTTCACCTAGATCCTTTTAAATTAAAAATGAAGTTTTAAATCAATCTAAAGTATATATGAGTAAACTTGGTCTGACAGTTACCAATGCTTAATCAGTGAGGCACCTATCTCAGCGATCTGTCTATTTCGTTCATCCATAGTTGCCTGACTCCCCGTCGTGTAGATAACTACGATACGGGAGGGCTTACCATCTGGCCCCAGTGCTGCAATGATACCGCGAGACCCACGCTCACCGGCTCCAGATTTATCAGCAATAAACCAGCCAGCCGGAAGGGCCGAGCGCAGAAGTGGTCCTGCAACTTTATCCGCCTCCATCCAGTCTATTAATTGTTGCCGGGAAGCTAGAGTAAGTAGTTCGCCAGTTAATAGTTTGCGCAACGTTGTTGCCATTGCTACAGGCATCGTGGTGTCACGCTCGTCGTTTGGTATGGCTTCATTCAGCTCCGGTTCCCAACGATCAAGGCGAGTTACATGATCCCCCATGTTGTGCAAAAAAGCGGTTAGCTCCTTCGGTCCTCCGATCGTTGTCAGAAGTAAGTTGGCCGCAGTGTTATCACTCATGGTTATGGCAGCACTGCATAATTCTCTTACTGTCATGCCATCCGTAAGATGCTTTTCTGTGACTGGTGAGTACTCAACCAAGTCATTCTGAGAATAGTGTATGCGGCGACCGAGTTGCTCTTGCCCGGCGTCAATACGGGATAATACCGCGCCACATAGCAGAACTTTAAAAGTGCTCATCATTGGAAAACGTTCTTCGGGGCGAAAACTCTCAAGGATCTTACCGCTGTTGAGATCCAGTTCGATGTAACCCACTCGTGCACCCAACTGATCTTCAGCATCTTTTACTTTCACCAGCGTTTCTGGGTGAGCAAAAACAGGAAGGCAAAATGCCGCAAAAAAGGGAATAAGGGCGACACGGAAATGTTGAATACTCATACTCTTCCTTTTTCAATATTATTGAAGCATTTATCAGGGTTATTGTCTCATGAGCGGATACATATTTGAATGTATTTAGAAAAATAAACAAATAGGGGTTCCGCGCACATTTCCCCGAAAAGTGCCAC

>pONN.AP3

TCGCGCGTTTCGGTGATGACGGTGAAAACCTCTGACACATGCAGCTCCCGGAGACGGTCACAGCTTGTCTGTAAGCGGATGCCGGGAGCAGACAAGCCCGTCAGGGCGCGTCAGCGGGTGTTGGCGGGTGTCGGGGCTGGCTTAACTATGCGGCATCAGAGCAGATTGTACTGAGAGTGCACCATATGCGGTGTGAAATACCGCACAGATGCGTAAGGAGAAAATACCGCATCAGGCGCCATTCGCCATTCAGGCTGCGCAACTGTTGGGAAGGGCGATCGGTGCGGGCCTCTTCGCTATTACGCCAGCTGGCGAAAGGGGGATGTGCTGCAAGGCGATTAAGTTGGGTAACGCCAGGGTTTTCCCAGTCACGACGTTGTAAAACGACGGCCAGTGCCAAGCTTCTAGGCTAGCATTAATACGACTCACTATAATGGCTGCGTGACACACACACGCAGCTTACGGGTTTCATACTGCTCTACTCTGCATTGCAAGAGATTAAAGTACCCATCATGGATTCAGTGTATGTAGACATAGATGCTGACAGCGCGTTTCTGAAGGCGTTGCAGCGAGCATACCCCATGTTTGAGGTGGAACCAAAGCAGGTCACGCCAAATGACCATGCAAACGCTAGAGCATTTTCGCATCTAGCAATAAAACTGATAGAGCAGGAAATTGATCCAGACTCAACCATTCTAGACATTGGTAGTGCACCAGCTAGGAGGATGATGTCTGATAGAAAATACCACTGCGTCTGCCCGATGCGCAGCGCAGAAGACCCTGAGAGGCTCGCGAATTACGCGAGAAAACTTGCATCAGCCGCTGGAAAGGTGACAGATAAAAACATCTCCGGAAAAATTAATGATCTACAGGCTGTGATGGCCGTACCGAATATGGAAACATCCACATTCTGCCTACACACTGATGCTACATGCAAACAAAGAGGAGACGTCGCCATTTATCAAGACGTCTACGCCGTCCATGCACCTACCTCGCTGTACCACCAGGCGATCAAAGGAGTCCGCGTGGCATATTGGATAGGGTTCGATACGACACCTTTCATGTACAATGCAATGGCTGGCGCATACCCATCATATTCAACAAACTGGGCTGATGAGCAGGTACTGAAGGCTAAGAATATAGGGCTGTGTTCAACAGACCTATCTGAGGGTAGACGAGGCAAATTATCTATCATGAGAGGCAAAAAATTGAAGCCATGCGACCGAGTGTTATTCTCGGTCGGCTCAACACTCTACCCTGAAAGTCGCAAACTTTTACAAAGCTGGCATTTACCATCGGTATTCCACCTGAAGGGTAAACTCAGCTTCACCTGCCGCTGTGACACGATTGTCTCATGCGAAGGATACGTCGTCAAGAGAGTGACGATGAGTCCAGGCATCTACGGAAAGACATCGGGGTATGCTGTAACTCATCATGCCGACGGCTTCCTGATGTGCAAGACGACAGATACAGTAGACGGTGAAAGGGTATCCTTCTCCGTGTGTACTTACGTACCAGCTACTATCTGCGATCAGATGACTGGAATCCTTGCCACTGAGGTAACCCCAGAAGACGCACAGAAACTACTGGTTGGGCTAAACCAACGGATAGTGGTCAATGGCAGGACGCAACGCAATACAAACACCATGAAAAATTACTTGCTCCCAATAGTTGCTCAGGCCTTCAGCAAGTGGGCCAAAGAATGTCGAAAGGACATGGAGGACGAAAAACTCTTGGGTGTCCGAGAGAGGACCTTAACATGCTGTTGCCTATGGGCATTCAGAAAGCATAAGACGCATACGGTGTACAAAAGACCGGATACCCAGTCAATCCAAAAGGTCCCTGCTGAATTTGACAGCTTCGTGATACCAAGCCTGTGGTCGTCAGGTTTATCAATCCCGCTGAGAACCAGAATCAAGTGGCTTTTGAGCAAAGCTCCAAAACACGAGCAACTACCACACAGCGGAAACGCAGAGGAAGCAGCTCAGGCTGAAATGGATGCAGCGGAAGAACGGGAGGCTGAGCTAACCCGAGAAGCTATGCCACCATTGCAAGCGACACAGGATGACGTTCAGGTAGAAATTGATGTAGAGCAACTTGAAGACCGAGCAGGAGCGGGCATAGTCGAAACACCAAGGGGAGCAATTAAAGTCACAGCCCAACCGTCAGACCGTGTTGTCGGAGAGTACTTAGTACTGACACCGCAGGCGGTCCTGCGCAGCCAAAAACTCAGTCTGATTCATGCGCTTGCGGAGCAGGTAAAAACGTGCACACATAGTGGGCGAGCAGGCAGGTACGCGGTTGAAGCATACGACGGGCGTGTTCTAGTGCCCTCGGGCTACGCGATACCCCAGGAAGATTTCCAGAGCTTAAGCGAAAGCGCCACTATGGTATTTAATGAGCGAGAGTTTGTGAACCGGAAGTTGCACCACATCGCCATGCACGGCCCAGCGCTGAACACCGATGAAGAGTCATACGAACTGGTAAGGGTTGAGAAAACAGAACACGAGTACGTCTATGACGTCGATCAGAAGAAATGCTGCAAGAGAGAGGAAGCAACAGGACTAGTGCTAGTAGGCGACTTAACTAGCCCACCATATCATGAGTTCGCCTACGAAGGACTAAAAATCCGCCCAGCATGTCCATACAAAACGGCGGTTATAGGTGTCTTCGGAGTACCGGGTTCCGGCAAGTCGGCTATAATCAAAAACCTGGTAACCAGGCAAGACTTGGTGACTAGTGGAAAAAAAGAAAACTGCCAAGAAATCTCCAATGATGTAATGCGGCAAAGGAAATTGGAGATATCTGCACGTACAGTCGACTCACTACTCCTGAATGGATGTAACAAGCCAGTGGAAGTACTGTACGTGGATGAGGCATTTGCTTGTCATTCGGGAACCCTGTTGGCACTGATAGCCATGGTTAGACCGCGTCAGAAGGTCGTACTTTGTGGCGACCCAAAGCAGTGCGGATTCTTCAATATGATGCAAATGAAGGTTAACTATAATCACAATATCTGCACACAGGTGTATCATAAAAGCATATCAAGGCGGTGTACACTGCCTGTAACAGCCATCGTGTCCTCGTTGCATTACGAGAGCAAGATGCGCACTACAAATGAGTACAACCAGCCAATCGTAGTGGATACTACGGGCATAACAAAACCAGAACCCGGGGACTTAGTGTTAACGTGTTTCCGGGGATGGGTTAAGCAGTTGCAAATAGACTACCGTGGAAACGAAGTCATGACAGCAGCTGCCTCTCAGGGGCTGACTAGAAAAGGTGTTTATGCAGTAAGGCAGAAAGTCAACGAAAACCCTCTGTATGCATCAACTTCAGAGCACGTTAACGTGTTATTGACACGCACAGAGGGCAAGTTGATATGGAAAACACTCTCGGGCGATCCATGGATAAAGATACTGCAGAACCCCCCAAAAGGGAACTTTAAGGCAACAATCAAGGAGTGGGAAGCGGAACACGCCTCCATTATGGCAGGAATATGCAATCACCAGATGGCTTTTGACACATTTCAGAACAAAGCTAATGTATGCTGGGCTAAATGCCTGGTCCCTATTCTTGACACCGCTGGAATCAAACTAAGTGACAGGCAGTGGTCTCAGATAGTGCAAGCTTTTAAAGAAGATAGGGCCTACTCTCCAGAAGTTGCACTGAATGAAATATGTACCCGCATATATGGGGTAGATCTGGACAGCGGACTATTTTCAAAGCCGCTGATATCCGTCTACTATGCAGACAACCACTGGGACAATAGACCAGGAGGAAAAATGTTCGGGTTCAACCCTGAGGTGGCACTTATGCTTGAAAAGAAATATCCCTTTACAAAAGGTAAGTGGAACATCAACAAGCAGATATGTATAACTACCAGAAAGGTTGACGAATTTAACCCCGAAACCAACATAATACCGGCCAACCGAAGACTGCCGCACTCACTCGTGGCTGAACACCATACAGTGAGAGGGGAAAGAATGGAATGGCTGGTAAACAAAATCAACGGTCACCACATGTTGTTGGTTAGTGGTTATAATCTTATATTACCAACAAAAAGAGTCACCTGGGTAGCACCGTTAGGCACCAGAGGTGCAGACTACACATATAACCTGGAACTTGGTCTACCAGCCACACTAGGCAGATATGACCTGGTAGTTATCAATATCCATACTCCATTCCGCATACATCATTACCAGCAGTGTGTAGATCACGCAATGAAGCTCCAGATGCTAGGGGGGGACTCTCTACGGCTGTTAAAGCCAGGAGGTTCACTTCTGATTAGAGCTTACGGGTACGCCGACCGAACCAGTGAAAGGGTCATTAGCGTATTGGGACGCAAGTTCAGATCGTCCAGGGCTCTAAAACCTCAGTGCATCACGAGCAATACAGAAATGTTCTTCCTATTTAGCCGATTCGACAACGGAAGAAGGAACTTCACCACACATGTTATGAACAACCAGCTGAATGCAGTGTATGCAGGACTGGCCACTAGAGCGGGCTGTGCTCCGTCATACCGTGTGAAACGGATGGACATCGCGAAAAACACTGAGGAATGCGTGGTAAACGCCGCCAATCCACGCGGAGTACCAGGCGATGGAGTATGTAAAGCCGTGTATAGAAAATGGCCAGAATCATTCAGAAACAGTGCAACGCCAGTGGGTACTGCAAAGACAATCATGTGCGGTCAATACCCCGTCATCCACGCGGTAGGCCCAAACTTCTCAAACTATTCTGAGGCTGAAGGGGATAGGGAATTGGCTTCAGTGTATAGAGAAGTGGCGAAAGAAGTGTCTAGGCTAGGAGTGAGCAGTGTAGCCATCCCTTTGCTTTCAACCGGTGTGTACTCAGGAGGCAAAGATAGACTGCTGCAATCACTAAACCATCTTTTCACAGCGATGGATTCGACAGATGCAGATGTTGTCATCTATTGCAGGGATAAGGAATGGGAGAAGAAGATCACTGAAGCCATATCATTAAGATCCCAGGTAGAACTACTAGATGATCACATCTCAGTGGATTGCGACATTGTACGCGTTCATCCAGACAGCAGCTTGGCAGGCCGAAAGGGGTACAGCACAGTAGAGGGAGCACTCTACTCGTACCTAGAGGGAACAAGATTCCACCAAACTGCTGTAGATATGGCAGAAATATATACCATGTGGCCAAAACAAACTGAAGCCAATGAACAGGTCTGCCTATATGCTCTGGGGGAGAGCATAGAGTCAGTCAGGCAAAAATGTCCTGTAGACGACGCCGACGCCTCATTCCCTCCGAAAACAGTCCCGTGCCTATGCCGTTATGCCATGACGCCTGAACGAGTTGCACGCCTACGCATGAATCATACTACTAGCATCATAGTGTGCTCGTCTTTTCCACTGCCGAAGTACAAAATCGAGGGCGTGCAAAAAGTAAAATGTTCAAAAGCACTCTTGTTTGATCACAACGTACCGTCTCGAGTGAGCCCGAGAACGTACAGGCCTGCGGACGAAATCATACAGACACCTCAAATACCAACTGAAGCGTGCCAGGACGCACAATTCGTGCAGTCAATAACTGATGAAGCAGTGCCAGTTCCGTCAGACTTAGAGGCTTGTGACGCAACTATGGACTGGCCCTCTATCGACATCGTACCAACAAGACAAAGAAGCGACTCATTTGACAGCGAGTATAGTTCCAGAAGTAACATACAGCTGGTGACAGCGGACGTGCATGCACCAATGTACGCAAATTCGCTGGCGTCCAGCGGAGGTTCAGTGCTGTCGCTGTCCAGTGAACAAGCTCAGAACGGCATAATGATACTACCTGATTCAGAAGACACAGATAGTATAAGCAGAGTAAGCACACCGATCGCCCCACCCAGGAGACGTTTGGGAAGGACTATAAATGTGACTTGTGACGAGCGGGAAGGGAAAATACTCCCTATGGCCAGCGACAGGCTCTTCACTGCTAAGCCATACACTGTCGCACTGGGCGTATCAACAGCAGACATAACTGCGTACCCCATCCAGGCACCGCTAGGATCGACACAACCGCCTGCCCTCGAACAGATCACTTTCGGAGATTTCGCCGAAGGTGAAATAGACAACCTCCTGACAGGGGCATTGACATTTGGAGACTTCGAGCCAGGTGAAGTGGAAGAGCTGACGGATAGCGAGTGGTCAACATGCTCGGACACAGATGAAGAGTTACGACTAGACAGAGCAGGGGGTTACATATTCTCCTCTGACACTGGTCAAGGTCATCTACAGCAAAAATCAGTACGTCAAACGACGCTACCGGTAAACATTGTTGAAGAGGTCCACGAAGAGAAATGCTACCCACCTAAATTGGATGAGATCAAAGAGCAACTCTTACTTAAGAGACTCCAGGAGAGTGCTTCCACGGCTAACCGGAGTAGGTACCAATCTAGAAAAGTGGAAAACATGAAAGCCACGATTATCCACAGACTGAAAGAGGGTTGCAGGCTCTACTTGGCGTCAGATACACCGAGGGTCCCATCTTACCGAATCACATACCCGGCGCCGGTCTACTCGCCTTCAATCAGTATCAAATTGAATAACCCAGAGACCGCAGTAGCAGTGTGTAATGAGTTTTTGGCCAGAAACTATCCAACTGTGGCATCCTACCAAGTTACTGACGAGTACGACGCGTACTTGGACATGGTAGACGGGTCCGAAAGCTGCCTAGACAGAGCTACATTTAACCCGTCTAAACTCAGGAGTTACCCAAAACAACACTCTTACCACGCACCCACCATCAGAAGTGCAGTGCCATCACCATTCCAAAATACGTTGCAGAATGTCTTGGCAGCGGCCACAAAAAGAAACTGCAACGTAACGCAGATGAGGGAATTGCCCACTATGGACTCCGCAGTGTTTAACGTGGAGTGTTTTAAGAAGTACGCATGCAACCAAGAGTACTGGAGAGAGTTCGCCTCAAGCCCTATAAGGGTAACGACAGAGAATCTGACAATGTATGTGACAAAACTAAAGGGGCCTAAAGCGGCGGCACTCTTCGCAAAAACACACAACTTGCTGCCGCTACAAGAGGTACCAATGGACAGGTTTACAATGGACATGAAACGTGATGTAAAAGTGACACCAGGTACAAAGCACACCGAGGAAAGGCCGAAAGTACAGGTCATACAGGCAGCAGAACCGCTGGCAACAGCATATCTGTGTGGCATACACAGAGAGTTGGTAAGAAGACTAAATGCAGTTCTGCTACCAAATGTCCACACACTGTTCGATATGTCAGCCGAAGATTTCGATGCAATCATATCTACACATTTCAAACCGGGCGATGCTGTACTAGAAACCGATATAGCCTCATTTGACAAGAGTCAAGACGATTCGCTTGCGCTGACCGCCATGATGCTGCTAGAAGACCTTGGGGTAGACCAACCTATCCTGGACCTGATAGAAGCAGCATTCGGCGAAATATCCAGTTGCCATCTACCGACGGGCACGCGGTTTAAGTTCGGCGCAATGATGAAATCAGGCATGTTTCTAACCCTGTTTGTCAATACCCTCCTGAACATCACCATTGCTAGTCGGGTGCTAGAGGAGCGATTGACTACTTCAGCCTGTGCAGCATTCATTGGGGACGACAACATAATACATGGAGTTGTCTCTGACGCACTAATGGCTGCACGTTGTGCTACGTGGATGAACATGGAAGTGAAAATCATCGATGCAGTAGTGTCAGAGAAGGCGCCATACTTCTGCGGGGGATTTATCTTACACGACACGGTGACAGGCACGTCGTGCAGAGTAGCAGACCCTTTAAAGAGACTGTTCAAGTTAGGCAAACCTCTGGCAGCTGGAGACGAACAGGATGAGGACAGAAGACGTGCTCTGGCAGATGAGGTTACTAGATGGCAAAGAACCGGCTTAATCACAGAATTAGAAAAAGCAGTATACTCCAGGTATGAAGTACAAGGAATAACAGCCGTAATAACATCAATGGCTACCTTTGCGAGTAGCAAAGAAAACTTTAAAAAACTAAGAGGGCCCGTCGTAACCTTGTACGGTGGACCTAAATAGGTACGCACTACAGCTACCTACTTTGAGACAAACATCGCTAATAGCCATGGAGTTCATACCAGCACAAACTTATTATAACAGAAGATACCAGCCTAGACCTTGGACCCAACGCCCTACTATCCAGGTGATCAGGCCAAAACCACGCCGGAGCAGGCCTGCAGGACAACTCGCACAACTGATATCCGCAGTCAGCAGACTAGCACTGCGTACAGTTCCTCAGAAACCACGCCGGACCCGAAAAACTAAGAAGCAAAAACAAGTGAAGCAAGAACAACAGAGCACTAGGAACCAGAAGAAAAAGGCGCCGAAACAAAAGCAGACTCAAAAGAAAAAGAGACCAGGACGCAGGGAAAGGATGTGCATGAAGATTGAAAATGACTGCATCTTCGAAGTCAAACATGAAGGAAAAATAACGGGGTATGCATGCCTAGTAGGTGATAAGGTAATGAAACCAGCACACGTGAAAGGGACTATTGACAATGCAGACCTAGCGAAGTTGGCGTTCAAAAGATCATCCAAGTATGATCTAGAATGCGCACAGATACCAGTGCACATGAAATCGGACGCCTCAAAGTTCACCCATGAAAAACCAGAAGGCTACTATAACTGGCATCACGGAGCGGTACAGTATTCTGGAGGGAGGTTTACAATCCCTACAGGCGCGGGAAAGCCTGGGGACAGCGGAAGACCAATCTTTGACAACAAGGGGCGCGTCGTGGCTATTGTCCTAGGCGGGGCAAACGAAGGAACCAGGACAGCACTATCTGTAGTGACTTGGAATAAAGACATAGTCACGAAAATCACACCGGAGGGGTCAGTTGAATGGAGCCTTGCCCTCCCTGTCATGTGCCTGTTGGCAAACACAACCTTCCCATGCTCCCAACCGCCTTGCGCGCCGTGCTGCTACGAAAAGAAACCGGAAGAAACCTTGAGGATGCTGGAGGACAACGTCATGCAACCAGGGTACTACCAGTTACTCGATTCAGCATTGGCCTGCTCACAACATCGTCAAAGACGCAATGCAAGAGAAAACTTCAATGTCTACAAAGTCACTAGGCCGTACTTAGCCCACTGCCCTGACTGCGGGGAGGGACACTCATGTCACAGCCCAATAGCATTAGAACGGATCAGAAGTGAGGCAACAGATGGTACCTTGAAAATCCAGGTATCTCTGCAAATCGGAATAAAGACAGACGACAGCCACGATTGGACGAAGCTGCGGTATATGGATAGCCATACACCTGTGGATGCAGACCGATCTGGTTTGTTTGTCAGAACGTCAGCACCGTGCACCATCACGGGAACGATGGGACATTTTATACTAGCACGCTGTCCGAAAGGAGAGACGCTGACGGTAGGATTTGTAGACAGTAGAAGGATCAGTCACACATGCATGCACCCGTTCCACCACGAGCCGCCGCTGATAGGGAGAGAGAAGTTTCACTCCCGCCCACAGCATGGCAAAGAACTACCTTGCAGTACGTACGTCCATACCACAGCGGCCACTACTGAGGAAATAGAAGTGCATATGCCGCCAGATACCCCTGACTACACGCTAATGACACAGCAAGCGGGAAACGTTAAGATCACAGTTGACGGCCAGACAGTACGATACAAGTGCAAATGTGACGGCTCCAATGAAGGATTAATAACCACTGACAAAGTCATAAATAACTGCAAAGTAGACCAATGCCACACAGCAGTTACAAACCACAAGAAATGGCAATACAACTCACCGTTGACCCCGCGGAACTCCGAACAAGGAGATAGGAAAGGTAAAATCCACATCCCATTTCCACTGGTAAACACAACCTGCAGGGTACCAAAAGCAAGAAATCCGACCATCACATACGGTAAAAACAGAGTCACTCTGCTGTTGTATCCAGACCATCCAACACTCCTTTCGTATCGCTCCATGGGAAGGATCCCGGATTACCATGAAGAGTGGATAACAAGTAAGAAGGAAATAAGTATCACAGTACCAGCAGAAGGCTTAGAGGTCACATGGGGTAATAATGACCCATACAAATATTGGCCCCAACTGTCTACAAATGGTACCGCGCACGGGCACCCACATGAAATAATCCTCTATTACTATGAGCTGTACCCAACTACCACAATTGCTGTACTGGCTGCTGCATCTATCGTAGTGGCATCTTTGGTAAGTCTATCATTAGGCATGTGCATATGCGCGAGACGCAGGTGCATCACGCCGTATGAGCTAACTCCGGGAGCTACCATCCCGTTCCTCCTAGGTGTACTATGCTGTGTCAAGACTGCAAAAGCAGCATCGTACTACGAAGCTGCAACATACCTCTGGAATGAGCAACAACCATTATTTTGGTTACAGCTTTTAATCCCTCTGTCAGCTGCAATTGTTGCGTGTAATTGCCTAAAACTTTTACCATGCTGCTGCAAAACATTGACTTTTTTAGCCGTTATGAGCATCGGTGCCCGCACTGTGTCCGCGTACGAGCACGCAACAGTGATCCCGAACACGGTGGGAGTACCGTATAAGACTCTTGTTAGCAGACCAGGGTATAGCCCTATGGTCTTAGAAATGGAGCTACAGTCGGTCACTCTGGAACCAACATTGTTCTTGGACTACATCACGTGTGAGTATAAAACAATCACACCGTCCCCGTACGTAAAATGCTGTGGTACAGCTGAATGTAAGGCCAAGAACCTACCAGATTATAACTGCAAAGTATTCACAGGCGTCTACCCATTCATGTGGGGAGGAGCATATTGCTTCTGTGACGCAGAGAACACACAACTCAGTGAGGCACACGTTGAGAAATCAGAATCATGCAAAACTGAGTTTGCATCAGCCTACAGAGCCCACACAGCTTCAGTATCAGCTAAACTACGTGTCTTTTACCAAGGGAATAATATCACTGTGTCTGCATACGCTAATGGTGATCACGCAGTCACGGTAAAGGACGCGAAGTTTGTCATCGGTCCACTATCGTCCGCCTGGTCACCATTTGATAATAAGATCGTGGTGTACAAAGGCGAAGTCTACAACATGGACTATCCACCTTTTGGCGCAGGGAGGCCAGGACAGTTCGGTGACATCCAGAGCCGCACGCCAGACAGCAAGGATGTCTATGCGAATACGCAGTTAATACTGCAAAGACCAGCGGCAGGAGCAATACACGTGCCTTACTCCCAGGCACCCTCGGGCTTTAAGTACTGGCTCAAAGAAAAAGGGGCATCATTGCAGCATACGGCACCATTTGGCTGTCAGATAGCAACAAACCCGGTAAGAGCAGTGAACTGTGCAGTGGGCAACATACCAGTCTCCATTGACATTCCAGATGCAGCTTTCACCAGGGTCACTGACGCTCCTTCCGTCACAGACATGTCCTGTGAAGTGGCTTCGTGTACCCATTCATCTGATTTTGGAGGTGCCGCAGTTGTAAAGTACACAGCTAGTAAAAAGGGGAAATGCGCTGTGCACTCTTTAACAAACGCGGTCACTATCCGCGAACCTAACGTAGATGTCGAGGGAACAGCACAACTACAGATTGCCTTCTCGACCGCACTAGCTAGTGCTGAATTTAAGGTGCAGATCTGCTCCACACAGGTACACTGCTCAGCGACATGCCATCCTCCTAAAGATCACATAGTCAACTACCCGTCACCGCACACCACACTGGGAGTGCAGGACATCTCAACGACAGCTATGTCTTGGGTCCAGAAGATTACAGGAGGAGTGGGACTCGTGGTTGCTATAGCTGCTTTGATCTTAATCATAGTTCTCTGCGTATCGTTTAGCAGACACTAAAGCATACATGCCCCAAAGTAACATACTATACAGGTATACGTGCTCCCTGAGCAGCACAATATATGTATTATCTATAAAAGAAAAACAAAAACATAAAAATTATAAAATACAAAAGTATAAAAACAGGTATCAGTACCCCCTTAGAGGTACATCACTTAACCAGGTATTAGTGCCCCCTTAGAGGCACATCATATAACCAGGTATAGGTGCCCCCTTAGAGGCACACTAACAATAGGTATAAGTGCCCCCTTAGTGGCACACTAACCACCACAATTGGTAATATGAAGAGACGTAGGTATGAAGCTTCATAAAAGCTGCCGAACTTACTTTAAGATGTAGGCGTACCGAACTCTTCGACAATTCTCCGAACCCGCAGGGACGTAGGAGAAGTTATTWTGTTTTTAATATTTCAAAAAAAAAAAAAAAAAAAAAAAAAGCGGCCGCCGCGGTGTCAAAAACCGCGTGGACGTGGTTAACATCCCTGCTGGGAGGATCAGCCGTAATTATTATAATTGGCTTGGTGCTGGCTACTATTGTGGCCATGTACGTGCTGACCAACCAGAAACATAATTGAATACAGCAGCAATTGGCAAGCTGCTTACATAGAACTCGCGGCGATTGGCATGCCGCCTTAAAATTTTTATTTTATTTTTTCTTTTCTTTTCCGAATCGGATTTTGTTTTTAATATTTCAAAAAAAAAAAAAAAAAAAAAAAAACGCGGAATTCGTAATCATGGTCATAGCTGTTTCCTGTGTGAAATTGTTATCCGCTCACAATTCCACACAACATACGAGCCGGAAGCATAAAGTGTAAAGCCTGGGGTGCCTAATGAGTGAGCTAACTCACATTAATTGCGTTGCGCTCACTGCCCGCTTTCCAGTCGGGAAACCTGTCGTGCCAGCTGCATTAATGAATCGGCCAACGCGCGGGGAGAGGCGGTTTGCGTATTGGGCGCTCTTCCGCTTCCTCGCTCACTGACTCGCTGCGCTCGGTCGTTCGGCTGCGGCGAGCGGTATCAGCTCACTCAAAGGCGGTAATACGGTTATCCACAGAATCAGGGGATAACGCAGGAAAGAACATGTGAGCAAAAGGCCAGCAAAAGGCCAGGAACCGTAAAAAGGCCGCGTTGCTGGCGTTTTTCCATAGGCTCCGCCCCCCTGACGAGCATCACAAAAATCGACGCTCAAGTCAGAGGTGGCGAAACCCGACAGGACTATAAAGATACCAGGCGTTTCCCCCTGGAAGCTCCCTCGTGCGCTCTCCTGTTCCGACCCTGCCGCTTACCGGATACCTGTCCGCCTTTCTCCCTTCGGGAAGCGTGGCGCTTTCTCAAAGCTCACGCTGTAGGTATCTCAGTTCGGTGTAGGTCGTTCGCTCCAAGCTGGGCTGTGTGCACGAACCCCCCGTTCAGCCCGACCGCTGCGCCTTATCCGGTAACTATCGTCTTGAGTCCAACCCGGTAAGACACGACTTATCGCCACTGGCAGCAGCCACTGGTAACAGGATTAGCAGAGCGAGGTATGTAGGCGGTGCTACAGAGTTCTTGAAGTGGTGGCCTAACTACGGCTACACTAGAAGAACAGTATTTGGTATCTGCGCTCTGCTGAAGCCAGTTACCTTCGGAAAAAGAGTTGGTAGCTCTTGATCCGGCAAACAAACCACCGCTGGTAGCGGTGGTTTTTTTGTTTGCAAGCAGCAGATTACGCGCAGAAAAAAAGGATCTCAAGAAGATCCTTTGATCTTTTCTACGGGGTCTGACGCTCAGTGGAACGAAAACTCACGTTAAGGGATTTTGGTCATGAGATTATCAAAAAGGATCTTCACCTAGATCCTTTTAAATTAAAAATGAAGTTTTAAATCAATCTAAAGTATATATGAGTAAACTTGGTCTGACAGTTACCAATGCTTAATCAGTGAGGCACCTATCTCAGCGATCTGTCTATTTCGTTCATCCATAGTTGCCTGACTCCCCGTCGTGTAGATAACTACGATACGGGAGGGCTTACCATCTGGCCCCAGTGCTGCAATGATACCGCGAGACCCACGCTCACCGGCTCCAGATTTATCAGCAATAAACCAGCCAGCCGGAAGGGCCGAGCGCAGAAGTGGTCCTGCAACTTTATCCGCCTCCATCCAGTCTATTAATTGTTGCCGGGAAGCTAGAGTAAGTAGTTCGCCAGTTAATAGTTTGCGCAACGTTGTTGCCATTGCTACAGGCATCGTGGTGTCACGCTCGTCGTTTGGTATGGCTTCATTCAGCTCCGGTTCCCAACGATCAAGGCGAGTTACATGATCCCCCATGTTGTGCAAAAAAGCGGTTAGCTCCTTCGGTCCTCCGATCGTTGTCAGAAGTAAGTTGGCCGCAGTGTTATCACTCATGGTTATGGCAGCACTGCATAATTCTCTTACTGTCATGCCATCCGTAAGATGCTTTTCTGTGACTGGTGAGTACTCAACCAAGTCATTCTGAGAATAGTGTATGCGGCGACCGAGTTGCTCTTGCCCGGCGTCAATACGGGATAATACCGCGCCACATAGCAGAACTTTAAAAGTGCTCATCATTGGAAAACGTTCTTCGGGGCGAAAACTCTCAAGGATCTTACCGCTGTTGAGATCCAGTTCGATGTAACCCACTCGTGCACCCAACTGATCTTCAGCATCTTTTACTTTCACCAGCGTTTCTGGGTGAGCAAAAACAGGAAGGCAAAATGCCGCAAAAAAGGGAATAAGGGCGACACGGAAATGTTGAATACTCATACTCTTCCTTTTTCAATATTATTGAAGCATTTATCAGGGTTATTGTCTCATGAGCGGATACATATTTGAATGTATTTAGAAAAATAAACAAATAGGGGTTCCGCGCACATTTCCCCGAAAAGTGCCACCTGACGTCTAAGAAACCATTATTATCATGACATTAACCTATAAAAATAGGCGTATCACGAGGCCCTTTCGTC
