# Supplementary material for: Identification of mosquito proteins that differentially interact with alphavirus nonstructural protein 3, a determinant of vector specificity
Source: PLoS Negl Trop Dis. 2023 Jan 25;17(1):e0011028. doi: 10.1371/journal.pntd.0011028 (PMC9876241; doi:10.1371/journal.pntd.0011028)
Supplement: S4 Fig — These are representative and there was no visible difference in the distribution of the different nsP3 constructs. Nuclei were stained with Hoechst 33342. (DOCX) [file pntd.0011028.s004.docx]

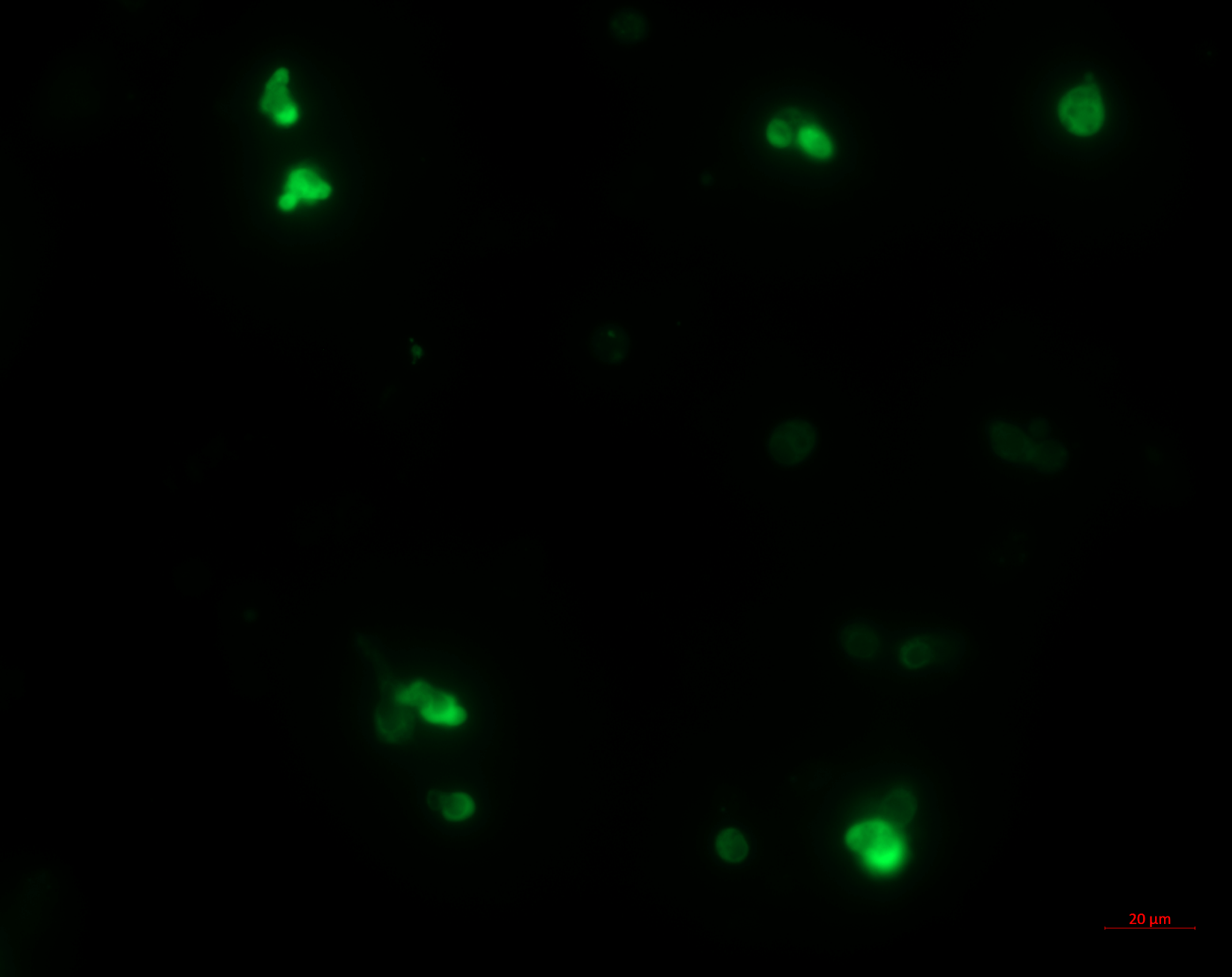

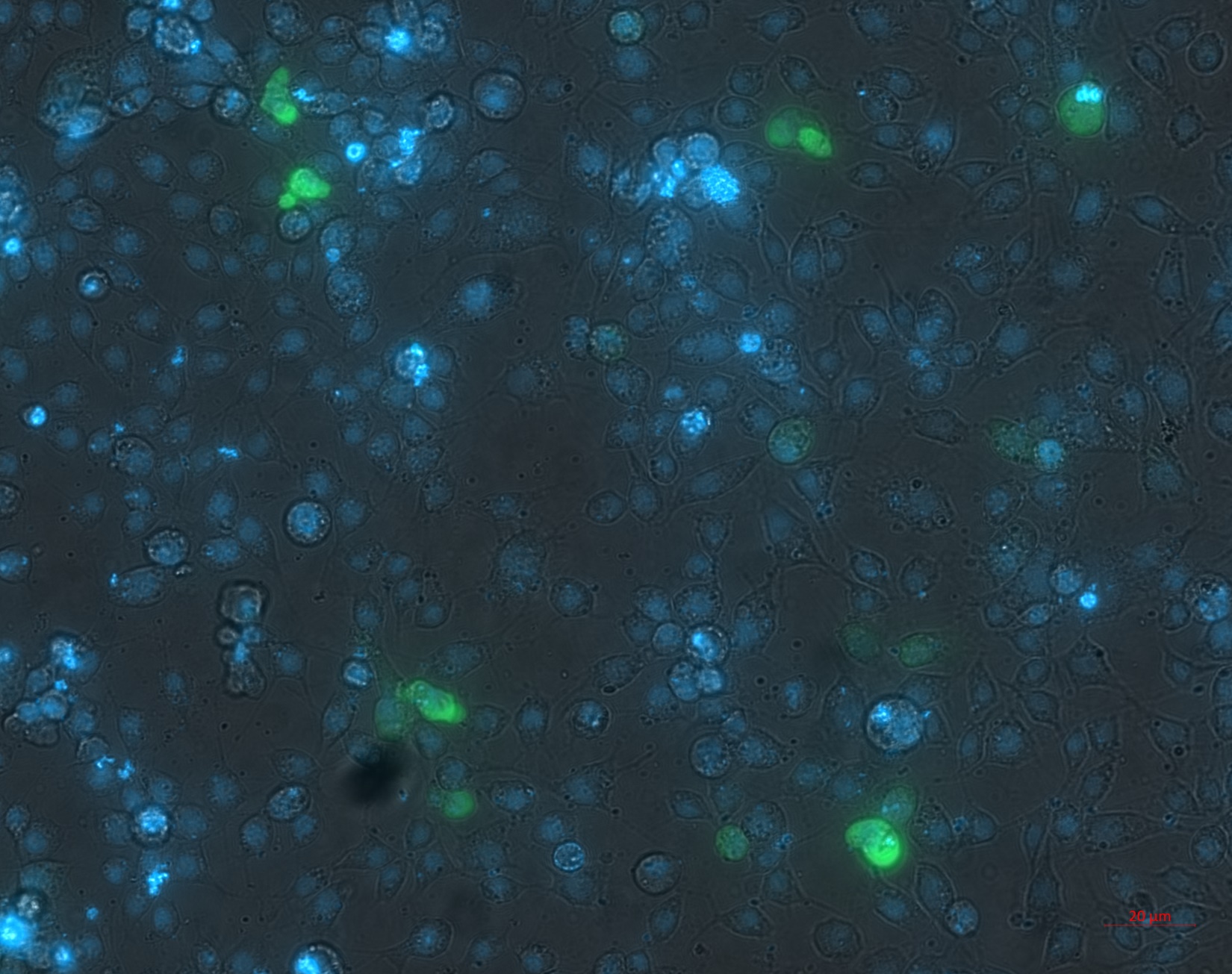


Supplemental Figure S4. Microscopy of live Sua 4.0 cells expressing GFP-HA-ONNVnsP3 opal, 24 hours post transfection. These are representative and there was no visible difference in the distribution of the different nsP3 constructs. Nuclei were stained with Hoechst 33342.
