# Supplementary material for: Identification of mosquito proteins that differentially interact with alphavirus nonstructural protein 3, a determinant of vector specificity
Source: PLoS Negl Trop Dis. 2023 Jan 25;17(1):e0011028. doi: 10.1371/journal.pntd.0011028 (PMC9876241; doi:10.1371/journal.pntd.0011028)
Supplement: S1 Table — (DOCX) [file pntd.0011028.s007.docx]

S1 Table. Table of primers used.

| **Primers for Cloning nsP3 into pIE1** | **COLOR CODE:** extra bases for restriction enzyme cleavage, restriction enzyme site, start or stop codon, HA Tag | | | |
| --- | --- | --- | --- | --- |
| **Name** | **Sequence** | **T_m-initial_** | **T_m-final_** | **Length** |
| **NMB.04** ClaI nsP3 ONN 4512 (+) | ctta atcgat g GCT CCG TCA TAC CGT GTG AAA C | 58.2°C | 62.9°C | 33 nt |
| Description: This forward primer added a ClaI site to the 5’ end of ONNV’s nsP3 (PCRed from pONN.AP3). It also added the start codon (since nsP3 lacks one; partially overlaps ClaI site), but no epitope tag. | | | | |
| **NMB.11** ClaI HA nsP3 ONN 4512 (+) | cata atcgat g tac cca tac GAC GTC CCA GAC TAC GCT CCG TCA TAC CGT GTG AAA C | 58.2°C | 69.2°C | 57 nt |
| Description: This forward primer added a ClaI site to the 5’ end of ONNV’s nsP3 (PCRed from pONN.AP3). It also added the start codon (since nsP3 lacks one; partially overlaps ClaI site) and an HA tag. The final ala in the HA tag (YPYDVPDYA) is the same as the first ala in nsP3 (APSY…) (seq here MYPYDVPDYAPSY…). The HA tag includes an AatII site (important for adding GFP). | | | | |
| **NMB.15** SalI Opal nsp3 ONN 6201(-) | cct gtcgac TCA TAA CTC TTC ATC TGT GTC CGA GC | 56.4°C | 64.3°C | 35 nt |
| Description: This reverse primer added a SalI site to the 3’ end of ONNV’s nsP3 (PCRed from pONN.AP3). It also added the opal stop codon in place of an arginine (…DEELX~~LDRAGG~~). | | | | |
| **NMB.06** SalI Arg nsP3 ONN 6243 (-) | cct gtcgac G ACC AGT GTC AGA GGA GAA TAT CTA ACC C | 54.8°C | 65.5°C | 38 nt |
| Description: This is a reverse primer that added a SalI site to the 3’ end of ONNV’s nsP3 (PCRed from pONN.AP3). It retained the existing arginine (no opal stop), but added an amber stop where the virus cleaves nsP3 from nsP4. | | | | |
| **NMB.08** ClaI nsP3 CHIK 4767 (+) | ctta atcgat g GCA CCG TCG TAC CGG G | 59.1°C | 64.2°C | 27 nt |
| Description: This forward primer added a ClaI site to the 5’ end of CHIKV’s nsP3 (PCRed from pCHIK.b). It also added the start codon (since nsP3 lacks one; partially overlaps ClaI site), but no epitope tag. | | | | |
| **NMB.12** ClaI HA nsP3 CHIK 4767 (+) | ctta atcgat g tac cca tac GAC GTC CCA GAC TAC GCA CCG TCG TAC CGG G | 61.7°C | 70.5°C | 51 nt |
| Description: This forward primer added a ClaI site to the 5’ end of CHIKV’s nsP3 (PCRed from pCHIK.b). It also added the start codon (since nsP3 lacks one; partially overlaps ClaI site) and an HA tag. The final ala in the HA tag (YPYDVPDYA) is the same as the first ala in nsP3 (APSY…) (seq here MYPYDVPDYAPSY…) The HA tag includes an AatII site (important for adding GFP). | | | | |
| **NMB.09** SalI Opal nsP3 CHIK 6358 (-) | cct gtcgac CTA CCC ACC TGC CCT ATC TAG TC | 57.3°C | 65.9°C | 32 nt |
| Description: This reverse primer added a SalI site to the 3’ end of CHIKV’s nsP3 (PCRed from pCHIK.b). It retained the existing Opal stop (…DDELXLDRAGG) and added an additional stop codon (amber) at after DRAGG­­­­, where the virus normally cleaves nsP3 from nsP4. | | | | |
| **NMB.10** SalI Arg nsP3 CHIK 6358 (-) | cct gtcgac CTA CCC ACC TGC CCT ATC TAG TCG TAA TTC G | 59.0°C | 67.2°C | 40 nt |
| Description: This reverse primer added a SalI site to the 3’ end of CHIKV’s nsP3 (PCRed from pCHIK.b). It swapped the existing Opal stop to an arginine (…DDELRLDRAGG) and added a stop codon (amber) at after LDRAGG (since the nsP3 lacks a stop codon), where the virus normally cleaves nsP3 from nsP4. | | | | |
| **NMB.13** ClaI pGFP-HA 1307(+) | cata atcgat G GGA TCC GTG AGC AAG GG | 59.7°C | 62.3°C | 28 nt |
| Description: primer for amplifying eGFP-HA from pGFP-HA – ClaI fwd | | | | |
| **NMB.14** pGFP-HA 2058(-) | GCG TAG TCT GGG ACG TCG TAT GGG | 62.8°C | 62.8°C | 24 nt |
| Description: rev primer for amplifying GFP from pGFP-HA – uses natural AatII site. | | | | |
| **Sequencing Primers** | | | | |
| **NMB.01** pIE1 seq 1099 (+) | GAC ACT GGC GGC GAC AAG | 59.4°C | 59.4°C | 18 nt |
| Description: This is the forward primer for sequencing the gene of interest in pIE1. It lays down at the 3’ end of the IE1 promoter. | | | | |
| **NMB.02** pIE1 seq 1325 (-) | GCT CGA ATT CTA CTC GTA AAG CCA G | 57.4°C | 57.4°C | 25 nt |
| Description: This is the reverse primer for sequencing the gene of interest in pIE1. It lays down at the 3’ end of the p35 polyadenylation signal. | | | | |
| **NMB.16** HA tag fwd primer | TAC CCA TAC GAC GTC CCA GA | 57.2°C | 57.2°C | 20 nt |
| Good for sequencing the HA constructs (pIE1/hr5/[gfp] HA-[ONN or CHIK] nsP3/PA) | | | | |
