# Supplementary material for: Identification of mosquito proteins that differentially interact with alphavirus nonstructural protein 3, a determinant of vector specificity
Source: PLoS Negl Trop Dis. 2023 Jan 25;17(1):e0011028. doi: 10.1371/journal.pntd.0011028 (PMC9876241; doi:10.1371/journal.pntd.0011028)
Supplement: S2 Table — (DOCX) [file pntd.0011028.s008.docx]

| Experiment shown in | Container type | area (cm^2^) | µg DNA | µL Fugene | cells plated | DNA µg/cm^2^ | Fugene µL/cm^2^ |
| --- | --- | --- | --- | --- | --- | --- | --- |
| Figure 2B | 12-well plate | 3.83 | 2 | 8 | 8 x 10^5 | 0.52 | 2.09 |
| Figure 3 and Table 1 | T25 | 25 | 13 | 52 | 5 x 10^6 | 0.52 | 2.08 |

Supplemental Table S2. Transfection details.
